# Design, Synthesis and Biological Exploration of Novel *N*-(9-Ethyl-9*H*-Carbazol-3-yl)Acetamide-Linked Benzofuran-1,2,4-Triazoles as Anti-SARS-CoV-2 Agents: Combined Wet/Dry Approach Targeting Main Protease (M^pro^), Spike Glycoprotein and RdRp

**DOI:** 10.3390/ijms252312708

**Published:** 2024-11-26

**Authors:** Ameer Fawad Zahoor, Saba Munawar, Sajjad Ahmad, Fozia Iram, Muhammad Naveed Anjum, Samreen Gul Khan, Jamila Javid, Usman Nazeer, Mashooq Ahmad Bhat

**Affiliations:** 1 Department of Chemistry, Government College University Faisalabad, Faisalabad 38000, Pakistan; fawad.zahoor@gcuf.edu.pk (A.F.Z.);; 2 Department of Chemistry, University of Engineering and Technology Lahore, Faisalabad Campus, Faisalabad 38000, Pakistan; 3 Department of Chemistry, Lahore College for Women University, Lahore 54600, Pakistan; 4 Department of Applied Chemistry, Government College University Faisalabad, Faisalabad 38000, Pakistan; 5 Department of Chemistry, University of Sialkot, Sialkot 51310, Pakistan; 6 Department of Chemistry, University of Houston, 3585 Cullen Boulevard, Houston, TX 77204, USA; 7 Department of Pharmaceutical Chemistry, College of Pharmacy, King Saud University, Riyadh 11451, Saudi Arabia

**Keywords:** SARS-CoV-2, carbazole, triazole, molecular docking, ADMET analysis

## Abstract

A novel series of substituted benzofuran-tethered triazolylcarbazoles was synthesized in good to high yields (65–89%) via *S*-alkylation of benzofuran-based triazoles with 2-bromo-*N*-(9-ethyl-9*H*-carbazol-3-yl)acetamide. The inhibitory potency of the synthesized compounds against SARS-CoV-2 was evaluated by enacting molecular docking against its three pivotal proteins, namely, M^pro^ (main protease; PDB ID: 6LU7), the spike glycoprotein (PDB ID: 6WPT), and RdRp (RNA-dependent RNA polymerase; PDB ID: 6M71). The docking results indicated strong binding affinities between SARS-CoV-2 proteins and the synthesized compounds, which were thereby expected to obstruct the function of SARS proteins. Among the synthesized derivatives, the compounds **9e**, **9h**, **9i**, and **9j** exposited the best binding scores of −8.77, −8.76, −8.87, and −8.85 Kcal/mol against M^pro^, respectively, −6.69, −6.54, −6.44, and −6.56 Kcal/mol against the spike glycoprotein, respectively, and −7.61, −8.10, −8.01, and −7.54 Kcal/mol against RdRp, respectively. Furthermore, the binding scores of **9b** (−8.83 Kcal/mol) and **9c** (−8.92 Kcal/mol) against 6LU7 are worth mentioning. Regarding the spike glycoprotein, **9b**, **9d**, and **9f** expressed high binding energies of −6.43, −6.38, and −6.41 Kcal/mol, accordingly. Correspondingly, the binding affinity of **9g** (−7.62 Kcal/mol) against RdRp is also noteworthy. Furthermore, the potent compounds were also subjected to ADMET analysis to evaluate their pharmacokinetic properties, suggesting that the compounds **9e**, **9h**, **9i**, and **9j** exhibited comparable values. These potent compounds may be selected as inhibitory agents and provide a pertinent context for further investigations.

## 1. Introduction

SARS-CoV-2 (Severe Acute Respiratory Syndrome Coronavirus-2) is classified under the family of coronaviruses known as *Coronaviridae* [1,2,3]. It has ravaged the world since its emergence in 2019 as the infectious agent of coronavirus disease 2019 (COVID-19) [4]. This potentially deadly and highly contagious virus has spread to every region, infecting and claiming millions of lives [5,6]. The rapid spread of SARS-CoV-2 has raised unprecedented challenges in global health, society, and the economy [7,8,9]. As the scientific community is eager to crusade against this pandemic, a thorough understanding of the virology, transmission dynamics, treatment methodologies, and clinical manifestations of SARS-CoV-2 is crucial to aid the development of diagnostics, effective therapeutics, drugs, and vaccines [10,11,12,13,14,15,16].

Since its emergence, the coronavirus has evolved into several strains known as alpha, beta, eta, gamma, omicron, and delta [17,18,19]. Among these, delta and omicron have been declared as “concern variants” [20,21]. There have been enormous efforts made to repress the outrage caused by this pandemic, which have involved the development of vaccines and restricted lockdowns [22,23,24]. Until now, more than 11.6 billion vaccines have been administered globally to curb the pandemic, but the COVID-19 situation still needs attention [25,26]. The scientific community has made tremendous efforts in developing novel, potent compounds against SARS-CoV-2, targeting it at various phases of its cycle [27,28,29,30]. The structure of the coronavirus is comprised of a single-stranded positive-sense RNA, which is known to be larger than any other RNA virus [31,32]. The genome is packed in an envelope consisting of a spike protein (S), membrane protein (M), and envelope protein (E) [33]. The spike glycoprotein (S) mediates the entry of the coronavirus into the host cell, making it an attractive anti-viral target. The spike glycoprotein (S) consists of a receptor-binding domain (RBD), which is crucial for receptor recognition [34]. Besides this, the RNA-dependent RNA polymerase (RdRp) is accountable for replication/transcription processes, whereas the main protease (Mpro) is pivotal in mediating this process. Both RdRp and Mpro are regarded as significant anti-viral targets [35].

From the nascent stages of SARS-CoV-2, several drugs have been employed for its treatment [36]. Some of the FDA-approved drugs for SARS-CoV-2 that have been enlisted include lopinavir/ritonavir (protease inhibitor), tocilizumab (immunosuppressive), umifenovir (abelson kinase inhibitor), ACE1 and ACE2 (monocarboxypeptidase), ribavirin (RNA polymerase inhibitor), carmofur (inhibitor of main protease), and remdesivir/favipiravir (RdRp inhibitor) [37,38,39,40,41,42,43]. Ribavirin was the first tested anti-viral vaccine employed for MERS (Middle East Respiratory Syndrome) and SARS-CoV-2 patients, and it worked as an RNA polymerase inhibitor [43]. Additionally, ivermectin, molnupiravir, nelfinavir, chloroquine, and nirmatrelvir have also been employed for treating SARS-CoV-2. Combinatorial therapy is the most frequently recruited approach for treatment, as some of these drugs work less effectively alone, i.e., chloroquine, nafamostat, ritonavir, hydroxychloroquine, and lopinavir [39,44].

The mechanism of action of these drugs varies as per the viral target. The receptor–ligand interactions are considered the basis to understanding the mechanism of action of a drug. RdRp inhibitors, i.e., remdesivir, act as RNA templates in the form of RMP (remdesivir monophosphate) during RNA synthesis and thereby block the active site [45]. The mechanism of action of favipiravir is similar to that of remdesivir as it also inhibits replication of the viral genome [46]. The drugs targeting Mpro bind to the anchor site in the pocket of the main protease and inhibit its function [47]. The spike glycoprotein undergoes conformational changes, i.e., prefusion and post-fusion, upon interaction with the ligand to aid in entry of the virus into the host cell and successful pathogenicity [48]. Kinases mediate entry of the virus into cells by fusion to the endosomal membrane [49].

**Figure 1:**
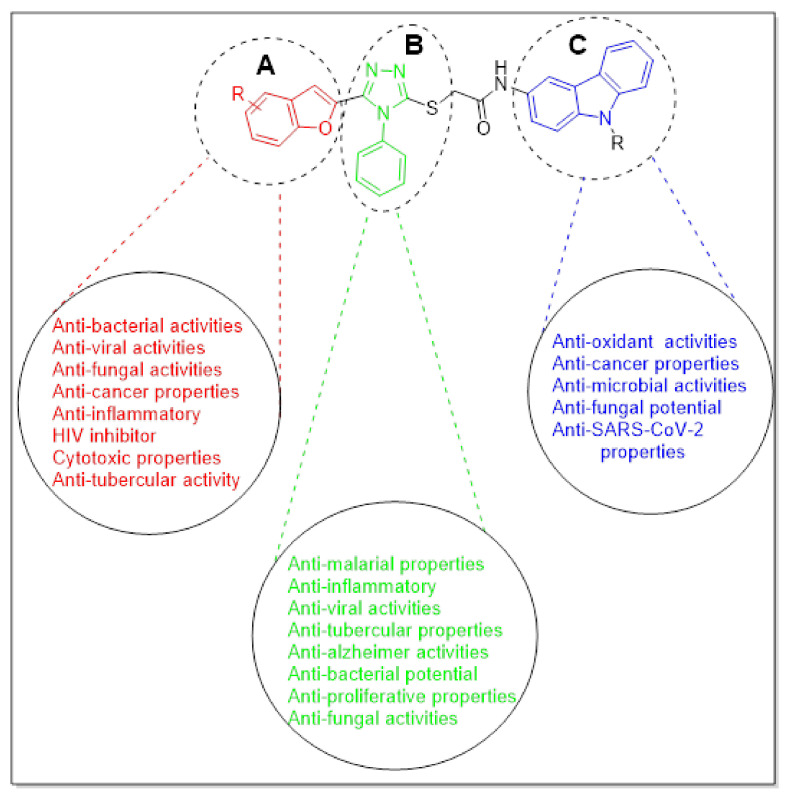
Literature survey-based biological effects of different moieties of benzofuran (**A**), triazole (**B**), and carbazole (**C**) scaffolds.

In addition to the efficacy of these drugs toward SARS-CoV-2 patients, these commonly used drugs also pose side effects, such as cardiovascular and hematopoietic systems, making patients more prone to being affected [50]. Besides medicines and vaccines, researchers have also discovered and synthesized various potent compounds against SARS-CoV-2 [51,52,53,54]. The purpose of this research paper is to present the synthesis of benzofuran-based carbazole derivatives and carry out docking studies against SARS-CoV-2 proteins to evaluate their anti-viral potential. The field of heterocyclic chemistry has shown progress in innovating feasible methodologies for the synthesis of diverse compounds [55,56]. The benzofuran-based carbazole derivatives consist of three major portions that individually possess numerous biological and medicinal attributes, as presented in Figure 1. The benzofuran scaffold has been found to express a wide array of biological activities [57,58,59]. The triazole moiety and its derivatives have articulated diversified medicinal properties [60,61,62,63,64,65]. Additionally, the carbazole skeleton acts as a key structural motif of synthetic derivatives and medicines, exhibiting a wide spectrum of therapeutic applications, as presented in Figure 1 [66,67,68,69]. These imperative scaffolds have even found profound applications against viral strains, making them ideal target for developing potential disrupters against SARS-CoV-2 [67,70,71,72].

In the search for efficient and better anti-SARS-CoV-2 drugs, researchers have triggered in silico drug designs, drug repurposing, and screening of novel libraries [73]. Conventional drug identification and development are time consuming and risky as these involve pre-clinical analyses to examine the efficacy and adverse effects of drugs [74,75]. Researchers have introduced various heterocyclic scaffolds bearing a number of biological applications [76]. However, computational screening techniques are efficient and powerful approaches for discovering potent compounds. These techniques enable the positioning of computer-generated receptors and ligands in various conformations and orientations to predict the best binding score-producing position and provide an analysis of the types of interactions unveiled between the receptor and ligand [77]. These fast-paced programs are progressively being used to screen libraries of novel compounds to ascertain potential drugs [78]. Given the above discussion, we have synthesized a novel series of benzofuran-based carbazole derivatives and performed molecular docking against selected SARS-CoV-2 proteins. The present study corresponds to the molecular docking of the synthesized derivatives and three proteins of SARS-CoV-2 (Mpro (main protease), the spike glycoprotein, and RNA-dependent RNA polymerase). The obtained results have been compared with the standard drugs employed for SARS-CoV-2 (remdesivir, lopinavir, chloroquine, favipiravir, and nirmatrelvir). These computationally proven studies may lead to the selection of potent compounds for cell line and preclinical studies to develop potentially disrupting agents against SARS-CoV-2.

## 2. Results and Discussion

### 2.1. Synthesis of Benzofuran-Based Carbazole Derivatives

**Scheme 1:**
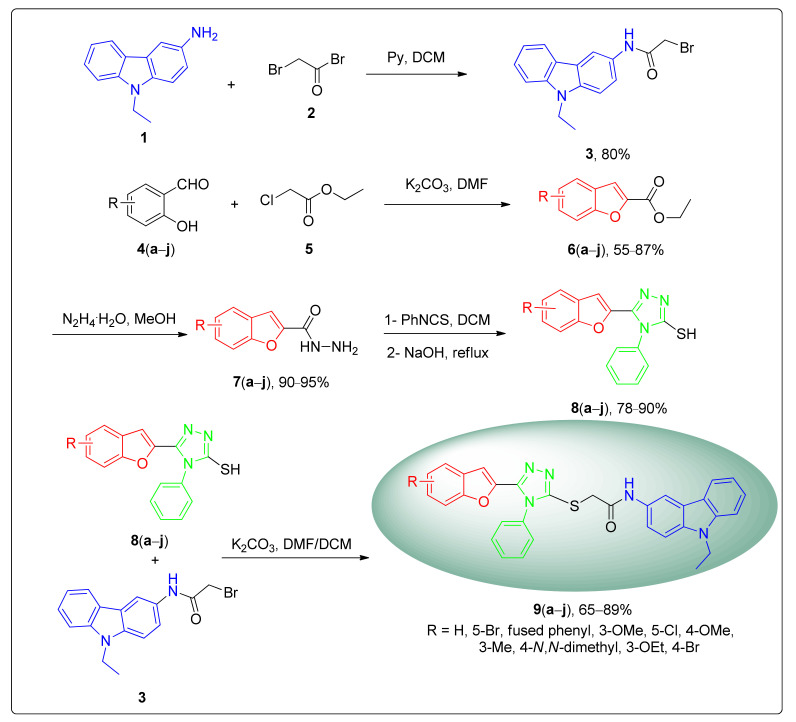
Synthesis of benzofuran-based carbazole derivatives **9**(**a**–**j**).

**Figure 2:**
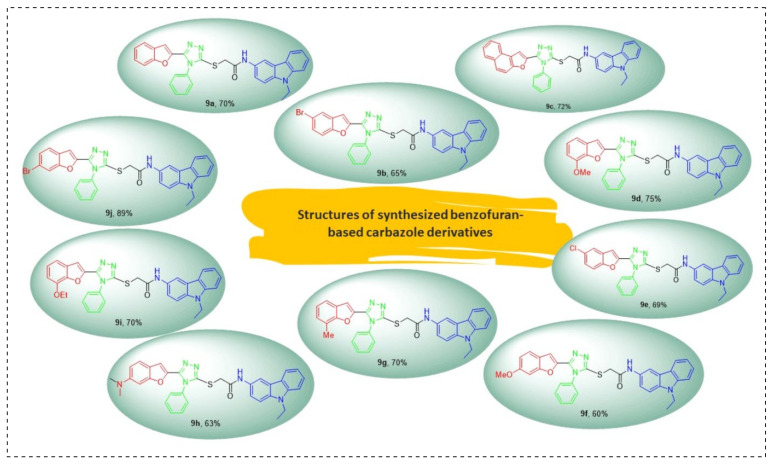
Structures of synthesized benzofuran-based carbazole derivatives **9**(**a**–**j**).

For the synthesis of benzofuran-based carbazole derivatives, carbazole acetamide **3** was synthesized from 9-ethyl carbazole-3-amine **1** and bromoacetyl bromide **2** in a solution of dichloromethane and pyridine as a first step [79,80]. In the next step, substituted salicylaldehydes **4**(**a**–**j**) were reacted with ethyl chloroacetate in the presence of potassium carbonate and DMF to synthesize substituted ethyl benzofuran-2-carboxylates **6**(**a**–**j**) in 55–87% yields [80]. Further, substituted benzofuran-2-carbohydrazides **7**(**a**–**j**) were synthesized in 90–95% yields from substituted ethyl benzofuran-2-carboxylates **6**(**a**–**j**) by overnight stirring in methanol [81]. The carbohydrazides **7**(**a**–**j**) were reacted with phenyl isothiocyanate in DCM to obtain thiosemicarbazide intermediates, which were further refluxed in a basic medium to furnish triazoles **8**(**a**–**j**) in 78–90% yields [82]. The last step involved the coupling of triazoles **8**(**a**–**j**) with carbazole acetamide **3** to give the final derivatives **9**(**a**–**j**) in good to high (65–89%) yields [79]. Scheme 1 contains the complete synthetic pathway employed for the synthesis of the target compounds. All of the synthesized derivatives are presented in Figure 2.

### 2.2. Spectral Elucidation of Synthesized Compounds

Characterization of the synthesized carbazole-based triazole analogs was performed by ^1^H and ^13^C NMR spectroscopy. The methyl and ethyl substitutions appeared at 1.03–1.37 ppm toward the upfield region in triplet form. The sharp singlet peak in the 4.00 ppm region corresponded to the methylene group present in the -S-CH_2_-C=O region. The quartet of the methylene group in the *N*-ethyl region of carbazole was noted in the >4.00 ppm region. Furthermore, the signals observed in the region of 7.00–8.5 ppm were assigned to the aromatic regions of carbazole and benzofuran rings. Lastly, all of the spectra of the synthesized derivatives contained a clear singlet of the characteristic secondary amine peak at >10.00 ppm. The protons of derivatives resonated mostly in the form of sharp singlet (s) peaks, doublets (d), doublets of doublets (dd), doublets of triplets (dt), or multiplets (m).

The carbon spectra of the synthesized derivatives consisted of a range of peaks originating from methyl, methylene, aromatic, and amine regions. The ^13^C NMR peaks observed at lower ppm values corresponded to less substituted carbons, like methyl and ethyl. The compounds containing methoxy and ethoxy substitutions were noted to have peaks in the region of 50.00–65.00 ppm. The *N*,*N*-dimethyl peak was observed at a chemical shift value of 44.00 ppm. The chemical shift value for the furanyl carbon was observed in the >150.00 ppm region. The ^13^C chemical shift values from 110.00 to 145.00 ppm corresponded to aromatic region peaks. In most cases, the signals originated at chemical shift values >160 ppm harmonized to the carbonyl region. The proton and carbon spectra (Supplementary Figures S1–S20) are given in the Supplementary File.

### 2.3. Molecular Docking Studies Toward SARS-CoV-2 Proteins

All carbazole derivatives (**9a**–**j**) were subjected to molecular docking studies along with selected FDA-approved drugs, which were taken as standards to provide a comparative study. To carry out the docking studies, three proteins of SARS-CoV-2 were selected as targets. These proteins were the main protease, which is responsible for viral reproduction, the spike glycoprotein, which is responsible for virion attachment to host cells, and RNA-dependent RNA-polymerase, which is responsible for transcription and translation processes. The docking results of the synthesized compounds were compared with FDA-approved drugs, i.e., remdesivir, lopinavir, chloroquine, favipiravir, and nirmatrelvir. For conducting the docking studies, MOE2022.02 software was utilized and the final images were extracted via DiscoveryStudio2024 v24.1.0.23298 software. The RMSD value was calculated before conducting molecular docking. The process of blind docking was employed to gain a complete perception of the anti-viral potential of the synthesized compounds.

#### 2.3.1. SARS-CoV-2 Main Protease

**Figure 3:**
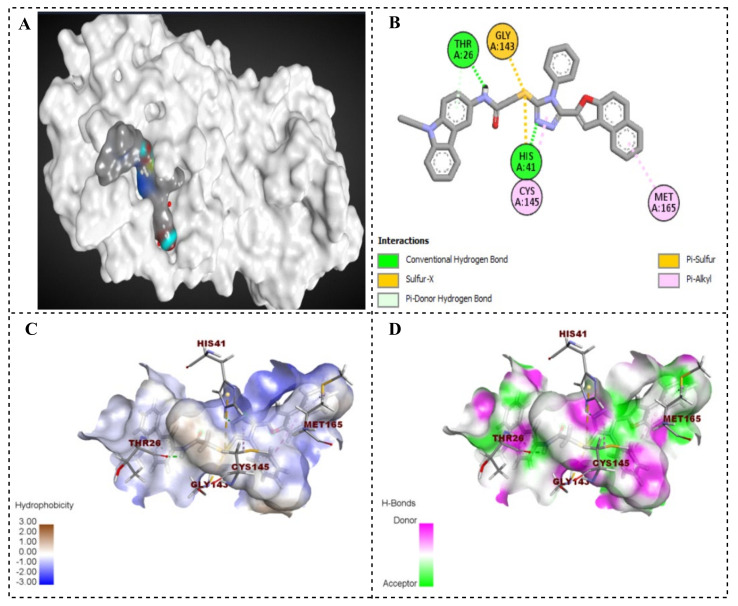
Interaction modes of compound **9c**: (**A**) Calculated surface of docked **9c** with the main protease, (**B**) 2D representation, (**C**) Hydrophobic interface, (**D**) Hydrogen bonding interface.

**Figure 4:**
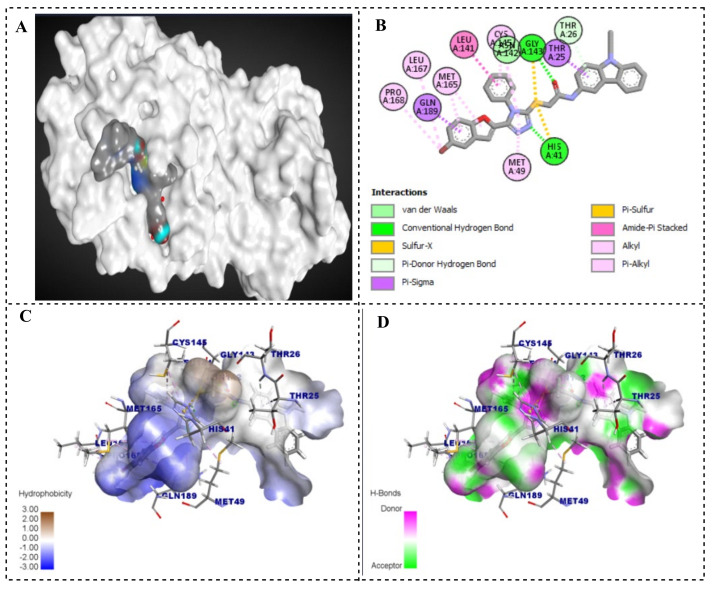
Interaction modes of compound **9b**: (**A**) Calculated surface of docked **9b** with the main protease, (**B**) 2D representation, (**C**) Hydrophobic interface, (**D**) Hydrogen bonding interface.

**Figure 5:**
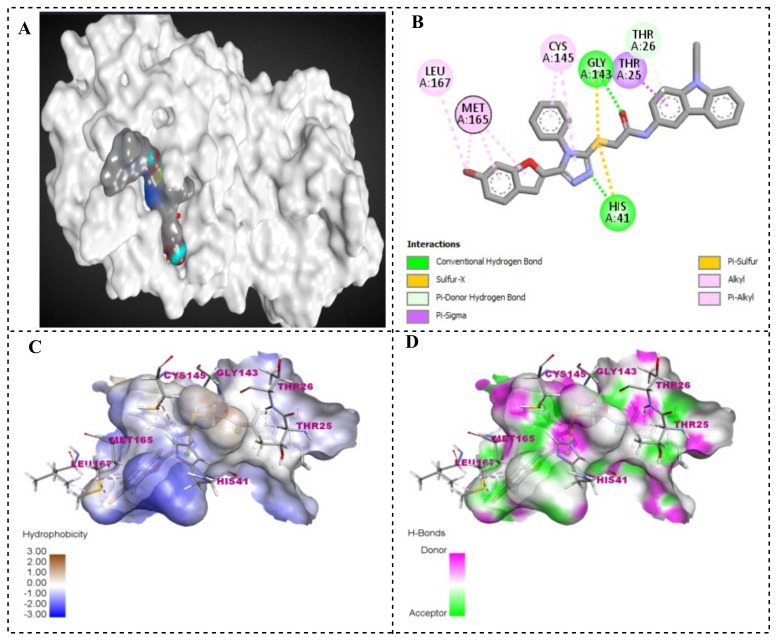
Interaction modes of compound **9j**: (**A**) Calculated surface of docked **9j** with the main protease, (**B**) 2D representation, (**C**) Hydrophobic interface, (**D**) Hydrogen bonding interface.

**Figure 6:**
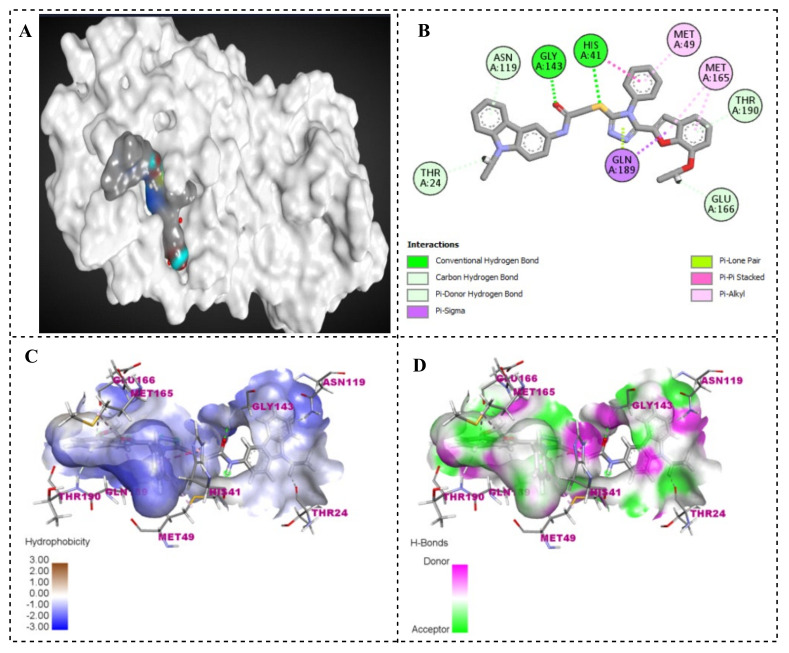
Interaction modes of compound **9i**: (**A**) Calculated surface of docked **9i** with the main protease, (**B**) 2D representation, (**C**) Hydrophobic interface, (**D**) Hydrogen bonding interface.

**Figure 7:**
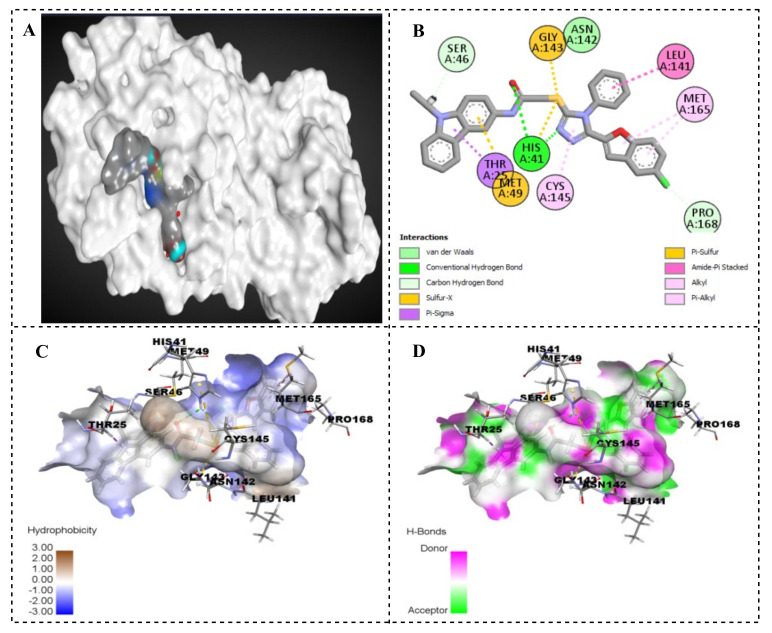
Interaction modes of compound **9e**: (**A**) Calculated surface of docked **9e** with the main protease, (**B**) 2D representation, (**C**) Hydrophobic interface, (**D**) Hydrogen bonding interface.

**Figure 8:**
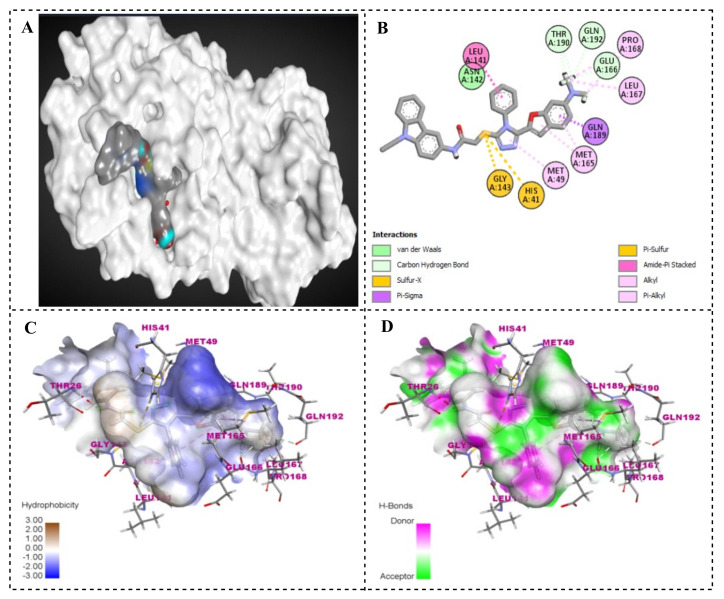
Interaction modes of compound **9h**: (**A**) Calculated surface of docked **9h** with the main protease, (**B**) 2D representation, (**C**) Hydrophobic interface, (**D**) Hydrogen bonding interface.

**Figure 9:**
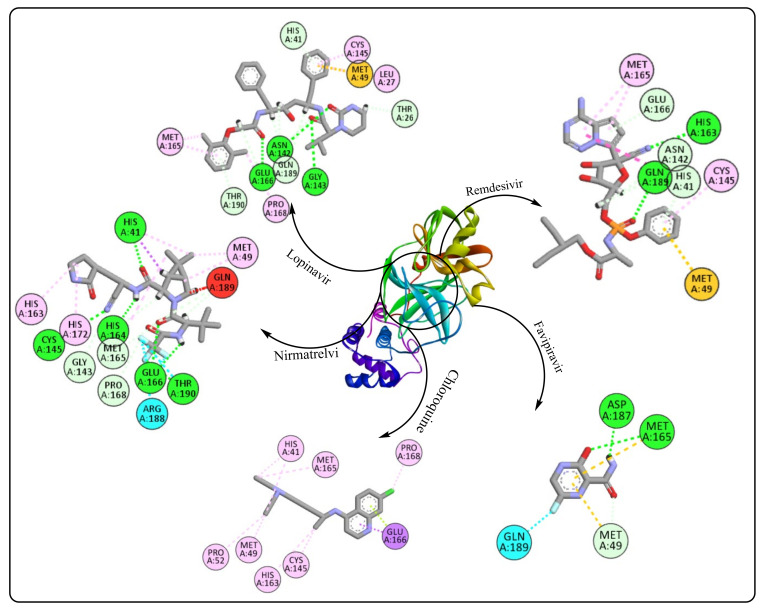
Two-dimensional diagram of standard drugs and their binding with the main protease (M^pro^).

The crystallographic structure of the main protease consists of a highly conserved pocket 1 [83]. This protein exists in the form of a complex with inhibitor N_3_. The native ligand of the protein was redocked to calculate the RMSD (root mean square deviation) value (1.2 Å) between the redocked and native ligand. The synthesized carbazole-based derivatives were docked with M^pro^ (main protease) protein using MOE2022.02 software with IFD (induced fit docking) [84]. Table 1 contains all of the interaction details of the potent compounds and standards with the main protease protein. Among the synthesized compounds, the derivatives **9b**, **9c**, **9e**, **9h**, **9i**, and **9j** exhibited high binding affinity of −8.83, −8.92, −8.77, −8.76, −8.87, and −8.85 Kcal/mol, respectively as compared with the standard drugs (Figure 3, Figure 4, Figure 5, Figure 6, Figure 7, Figure 8 and Figure 9). From the aforementioned compounds, **9c** possessed the highest binding affinity, revealing the formation of two hydrogen bonds with the residues (Figure 3). The carbonyl functionality of amino acid HIS41 was found to develop a hydrogen bond with the -N of the triazole ring at a distance of 2.15 Å. The formation of a second hydrogen bond occurred between the aliphatic -NH group of the derivatives and the carbonyl group of THR26 at a distance of 2.07 Å. HIS41 and GLY143 developed pi–sulfur and sulfur–X interactions with the -S- part of the derivatives at distances of 5.48 and 3.28 Å, respectively. The residue THR26 was shown to build a pi–donor hydrogen bond with the aromatic ring of the derivatives at a distance of 3.12 Å. Similarly, the pi–alkyl interaction was also noticed with the CYS145 and MET165 residues at distances of 5.15 and 4.66 Å, respectively. The amino acid residues involved in the development of hydrophobic interactions included THR26, HIS41, GLY143, CYS145, and MET165. The interaction analysis of the screened analog **9b** with M^pro^ revealed the similar formation of two hydrogen bonds between the carbonyl group of the derivatives and the -NH group of GLY143 and the second one being between the nitrogen atom of triazole and the carbonyl group of HIS41 at distances of 2.28 and 2.19 Å, respectively (Figure 4). The formation of sulfur–X and pi–sulfur interactions with GLY143 and HIS41 was also observed at distances of 3.19 and 5.60 Å, respectively. The amino acid residue THR26 formed a pi–donor HB with an aromatic ring of carbazole at a distance of 3.27 Å. The amino acid residue LEU141 exhibited an amide pi-stacked mapping with the phenyl group of the derivatives at a distance of 4.44 Å. Besides this, alkyl, pi–sigma, and pi–alkyl interactions were also observed. The hydrophobic and hydrogen bonding interactions indicated the involvement of amino acid residues THR26, HIS41, GLY143, CYS145, and MET165. A similar pattern of interactions was observed for **9j** (Figure 5). The interaction analysis of **9i** revealed the formation of two hydrogen bonds with the amino acids of M^pro^ (Figure 6). Among these, the formation of one hydrogen bond occurred between the sulfur group of the derivatives and HIS41 (dHb = 2.74 Å) and the other one was between the carbonyl group of the derivatives and -NH of GLY143 (dHb = 2.28 Å). The formation of carbon–hydrogen bonds and pi–donor hydrogen bonds was also noticed with amino acid residues THR24, ASN119, THR190, and GLU166. Other than hydrogen bonding interactions, other pi interactions were also exhibited, including pi–sigma, pi–lone pair, pi–pi stacked, and pi–alkyl interactions. The amino acids involved in hydrophobic interactions were THR24, ASN119, GLY143, HIS41, MET49, GLN189, THR190, MET165, and GLU166. The compounds **9e** and **9h** also exhibited strong binding affinities with the main protease i.e., −8.78 and −8.76 Kcal/mol, respectively (Figure 7 and Figure 8). Among the approved drugs, lopinavir exhibited the highest affinity toward Mpro, with a binding affinity of −8.7372 Kcal/mol. All other carbazole derivatives exhibited slightly lesser or equivalent binding energy to lopinavir. Besides this, all of the synthesized derivatives exhibited more potency than chloroquine and favipiravir. The docking results obtained for remdesivir and Mpro also revealed that all of the derivatives exhibited comparable binding energies to remdesivir. The 2D interaction details of the standard drugs are presented in Figure 9. The docking interactions of compounds **9a**, **9d**, **9f**, and **9g** are given in the (Supplementary Information Figures S21–S24).

#### 2.3.2. Spike Glycoprotein (6WPT)

**Figure 10:**
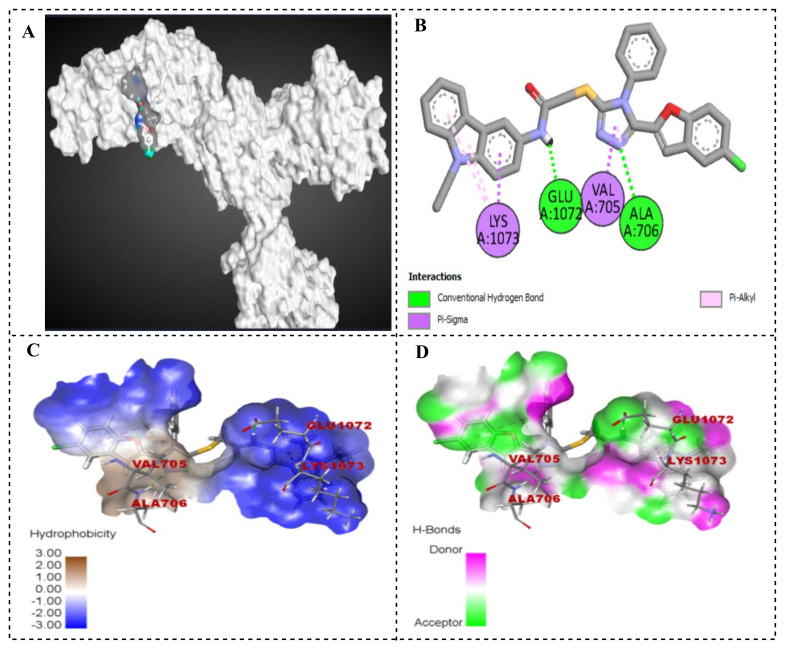
Interaction modes of compound **9e**: (**A**) Calculated surface of docked **9e** with the spike glycoprotein, (**B**) 2D representation, (**C**) Hydrophobic interface, (**D**) Hydrogen bonding interface.

**Figure 11:**
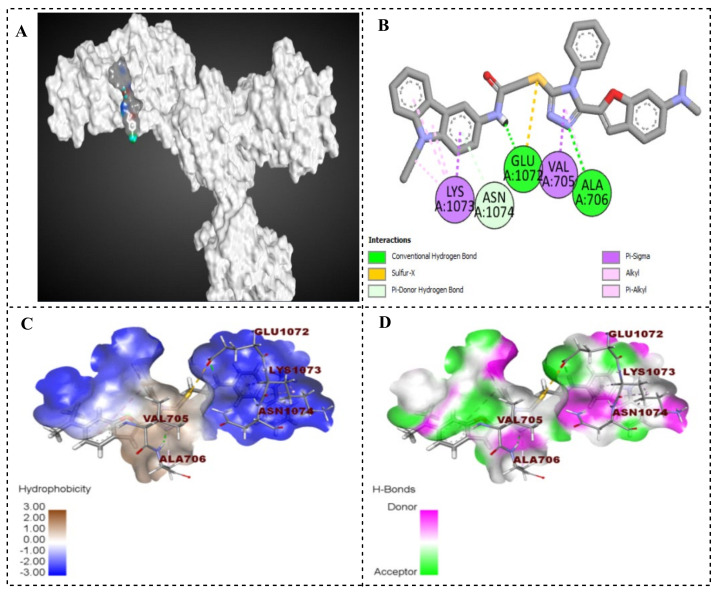
Interaction modes of compound **9h**: (**A**) Calculated surface of docked **9h** with the spike glycoprotein, (**B**) 2D representation, (**C**) Hydrophobic interface, (**D**) Hydrogen bonding interface.

**Figure 12:**
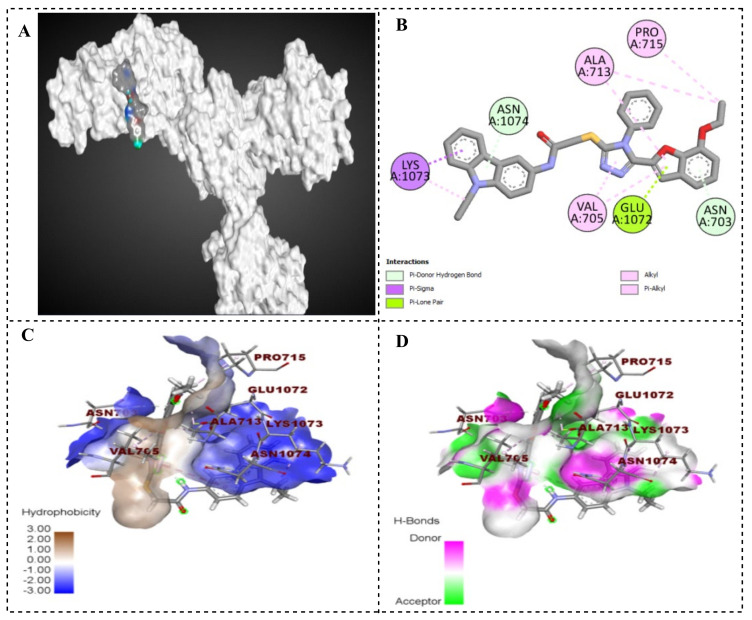
Interaction modes of compound **9i**: (**A**) Calculated surface of docked **9i** with the spike glycoprotein, (**B**) 2D representation, (**C**) Hydrophobic interface, (**D**) Hydrogen bonding interface.

**Figure 13:**
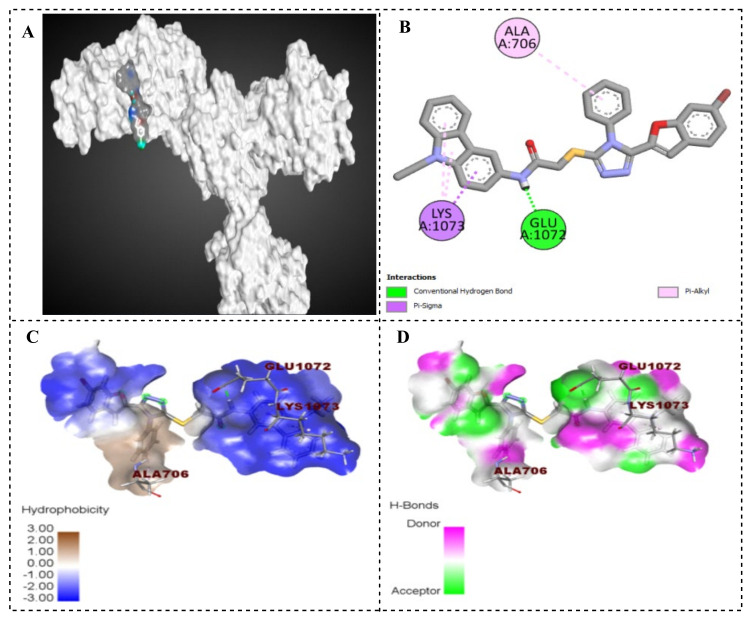
Interaction modes of compound **9j**: (**A**) Calculated surface of docked **9j** with the spike glycoprotein, (**B**) 2D representation, (**C**) Hydrophobic interface, (**D**) Hydrogen bonding interface.

**Figure 14:**
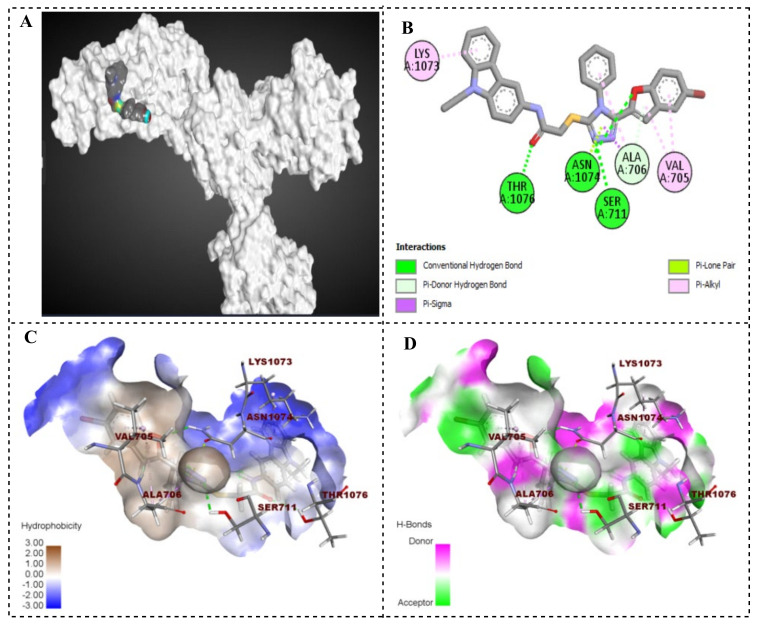
Interaction modes of compound **9b**: (**A**) Calculated surface of docked **9b** with the spike glycoprotein, (**B**) 2D representation, (**C**) Hydrophobic interface, (**D**) Hydrogen bonding interface.

**Figure 15:**
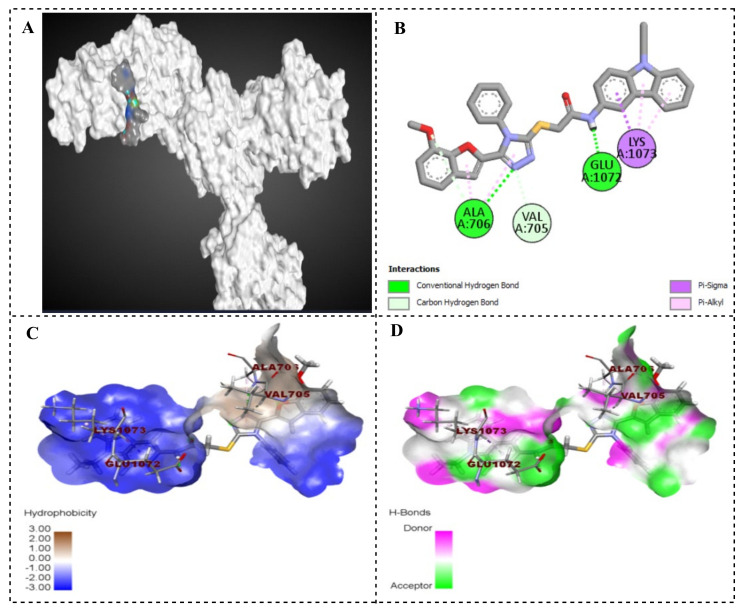
Interaction modes of compound **9d**: (**A**) Calculated surface of docked **9d** with the spike glycoprotein, (**B**) 2D representation, (**C**) Hydrophobic interface, (**D**) Hydrogen bonding interface.

**Figure 16:**
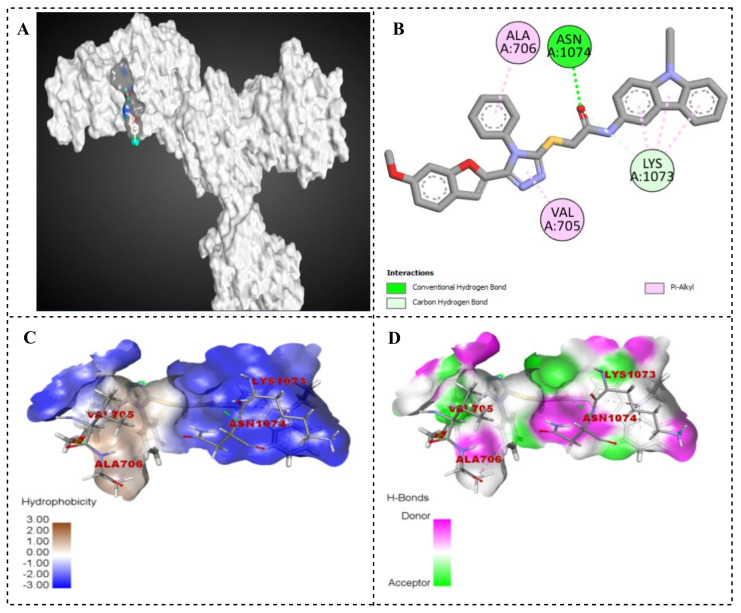
Interaction modes of compound **9f**: (**A**) Calculated surface of docked **9f** with the spike glycoprotein, (**B**) 2D representation, (**C**) Hydrophobic interface, (**D**) Hydrogen bonding interface.

**Figure 17:**
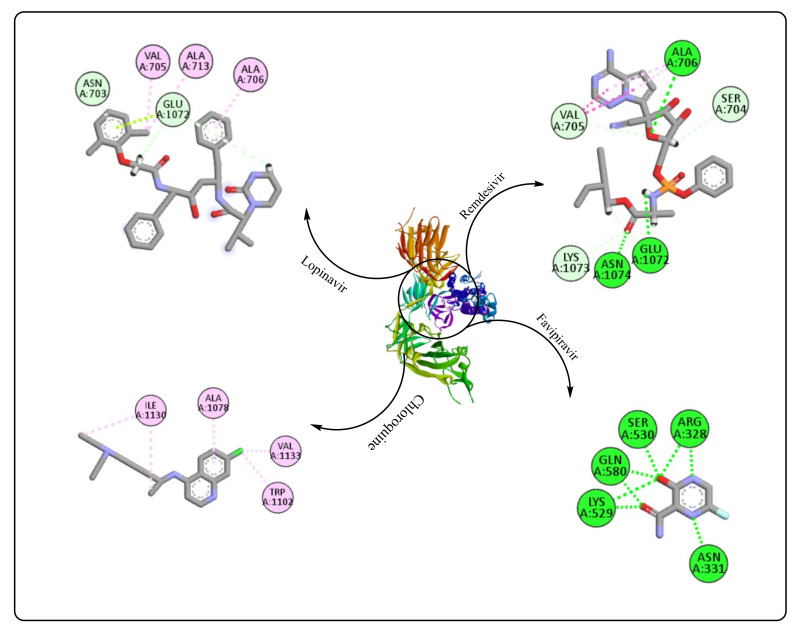
Two-dimensional diagram of standard drugs after binding with the spike glycoprotein.

The spike (S) glycoprotein consists of three monomer units and is known as a transmembrane homotrimer glycoprotein [84]. It aids in the binding of the virion to the host cell, thereby acting as a crucial target for the development of anti-viral drugs [85]. Molecular docking of the synthesized compounds with the spike glycoprotein (PDB ID: 6WPT) was executed using MOE2022.02 software. Induced fit docking was employed to dock the ligands with the protein. Before performing docking with the synthesized ligands, the protein was redocked to its native ligand for calculating the RMSD value (0.6 Å). The binding energy values obtained from docking of the spike glycoprotein with the synthesized series of carbazoles pointed out that most of the compounds i.e., **9b**, **9d**, **9e**, **9f**, **9h**, **9i**, and **9j** were found to exhibit potent binding energies compared even better than the standard drugs (Figure 10, Figure 11, Figure 12, Figure 13, Figure 14, Figure 15, Figure 16 and Figure 17). Table 2 contains the molecular docking, binding score, and interaction analysis of the potent compounds with the spike glycoprotein. The compound **9e** exhibited the highest binding score of −6.69 Kcal/mol (Figure 10). It formed two hydrogen bonds with the binding pocket of the spike glycoprotein; one between the -NH group of the derivatives and the carbonyl group of GLU1072 (dHb = 1.63 Å) and the other between the nitrogen of triazole and the carbonyl of ALA706 (dHb = 2.16 Å). The pi–sigma interactions involved the amino acid residues LYS1073 and VAL705 located at distances of 2.75 and 2.81 Å, respectively. The residues VAL705, GLU1072, LYS1073, and ALA706 were involved in the development of the hydrophobic interactions. Some pi–alkyl interactions were also formed with the LYS1073 residue. Similarly, **9h** also exhibited potent interaction with the spike glycoprotein, with a binding energy of −6.54 Kcal/mol (Figure 11). It formed two similar hydrogen bonds with GLU1072 at a distance of 1.70 Å and ALA706 at a distance of 2.16 Å. The amino acid residue GLU1072 was also noticed for the formation of sulfur–X interaction at a distance of 3.29 Å. The formation of a pi–donor hydrogen bond was also observed with the ASN1074 residue at a distance of 3.28 Å. The pi–sigma interaction was noticed with the GLU1072 and ALA706 residues. The amino acid residue LYS1073 was liable for the formation of alkyl and pi–alkyl mapping. The amino acid residues involved in hydrophobic interactions were GLU1072, LYS1073, ASN1074, VAL705, and ALA706. The interaction analysis of **9i** exhibited the formation of a pi–donor HB with the residues ASN1074 and ASN703, exhibiting a binding affinity of −6.44 Kcal/mol (Figure 12). The amino acid residue LYS1073 formed a pi–sigma interaction while GLU1072, forming pi–lone pair interface. Some pi–alkyl and alkyl mapping were also observed with the LYS1073, ALA713, PRO715, and VAL704 residues. The hydrophobic interactions were developed by the VAL705, ASN703, GLU1072, PRO715, LYS1073, ASN1074, and ALA713 residues. The compound **9j** interacted with the spike glycoprotein to form one hydrogen bond between the -NH group of the derivatives and the carbonyl group of GLU1072 at a distance of 1.62 Å (Figure 13). The residue LYS1073 was responsible for the pi–sigma interaction. Some pi–alkyl interactions with the LYS1073 and ALA706 residues were also observed. The hydrophobic interactions were developed by GLU1072, LYS1073, and ALA706. Besides these, the compounds **9b**, **9d**, and **9f** also displayed binding scores in the range of –6.39 to –6.44 Kcal/mol which were comparable to the standards (Figure 14, Figure 15 and Figure 16). The docking analysis of potent drugs, namely, remdesivir, chloroquine, favipiravir, and lopinavir, indicated that the binding energies of remdesivir and lopinavir were higher, i.e., −6.26 and −6.09 Kcal/mol, than the others. The binding affinities of all carbazoles were greater or comparable to the binding affinities of the standards, implying their potential usage as spike glycoprotein blockers. Figure 17 contains the two-dimensional interactions of the standard drugs with the spike glycoprotein. The docking interaction images of **9a**, **9c**, and **9g** are given in the (Supplementary Information Figures S25–S27).

#### 2.3.3. RNA-Dependent RNA Polymerase in Complex with Cofactors (6M71)

**Figure 18:**
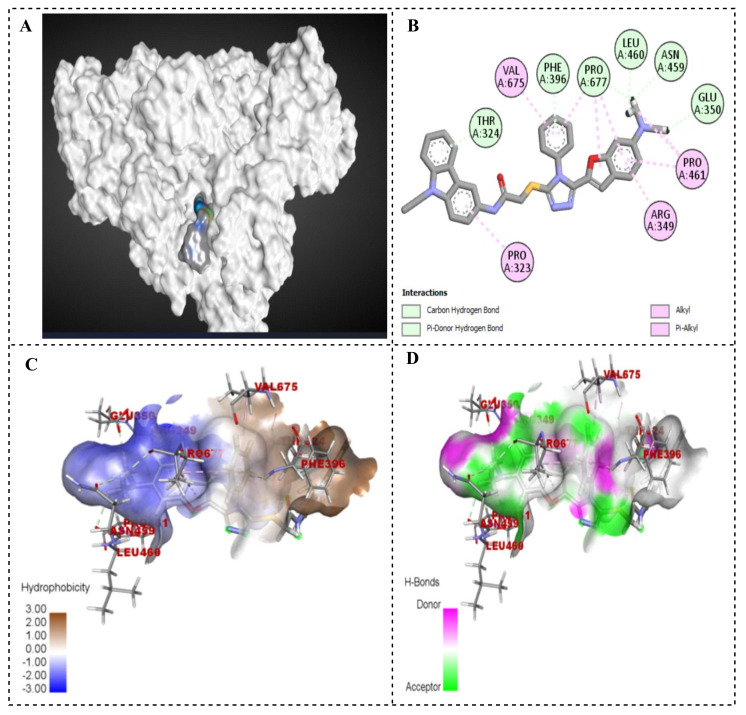
Interaction modes of compound **9h**: (**A**) Calculated surface of docked **9h** with the RNA-dependent RNA-polymerase, (**B**) 2D representation, (**C**) Hydrophobic interface, (**D**) Hydrogen bonding interface.

**Figure 19:**
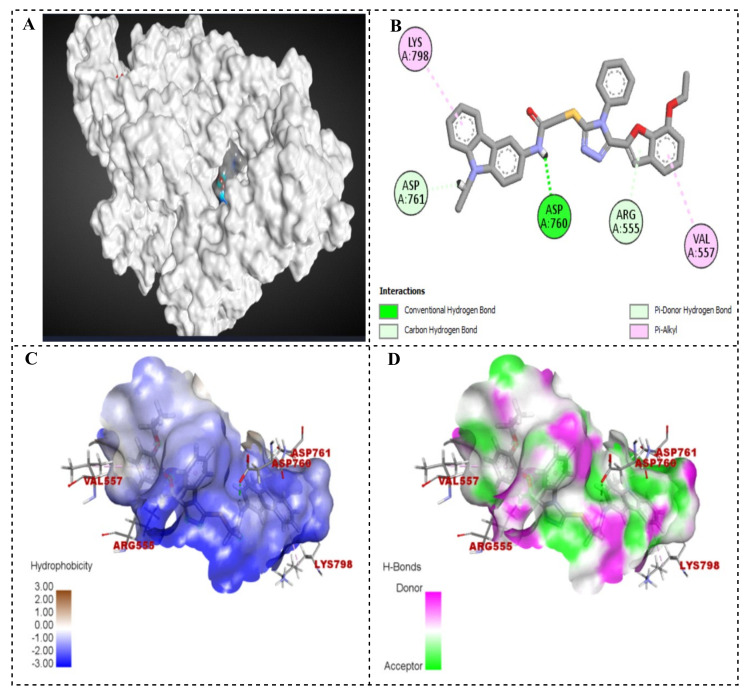
Interaction modes of compound **9i**: (**A**) Calculated surface of docked **9i** with the RNA-dependent RNA-polymerase, (**B**) 2D representation, (**C**) Hydrophobic interface, (**D**) Hydrogen bonding interface.

**Figure 20:**
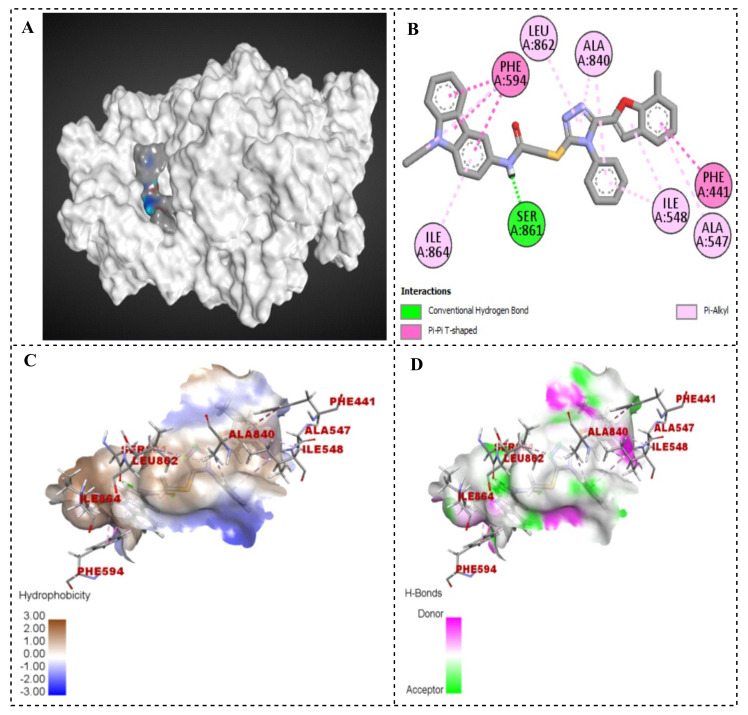
Interaction modes of compound **9g**: (**A**) Calculated surface of docked **9g** with the RNA-dependent RNA-polymerase, (**B**) 2D representation, (**C**) Hydrophobic interface, (**D**) Hydrogen bonding interface.

**Figure 21:**
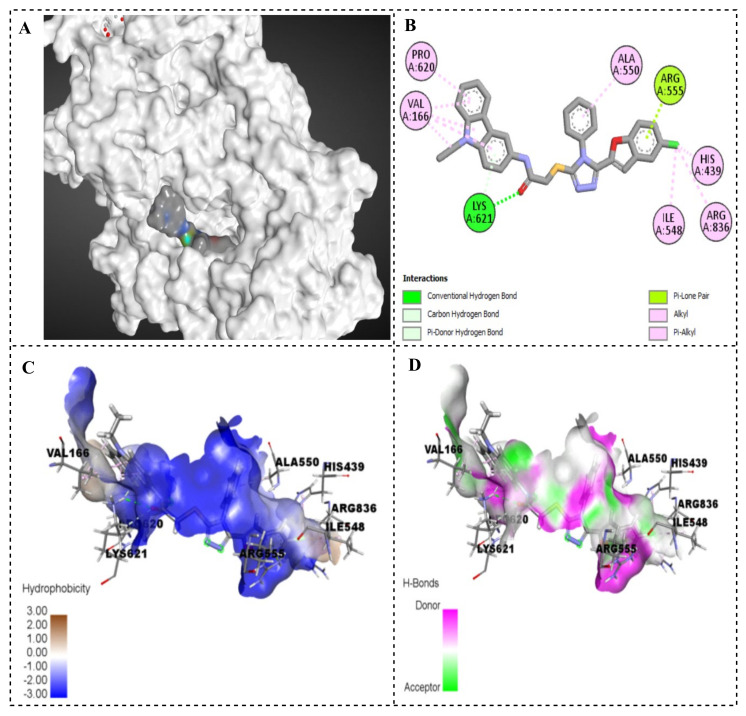
Interaction modes of compound **9e**: (**A**) Calculated surface of docked **9e** with the RNA-dependent RNA-polymerase, (**B**) 2D representation, (**C**) Hydrophobic interface, (**D**) Hydrogen bonding interface.

**Figure 22:**
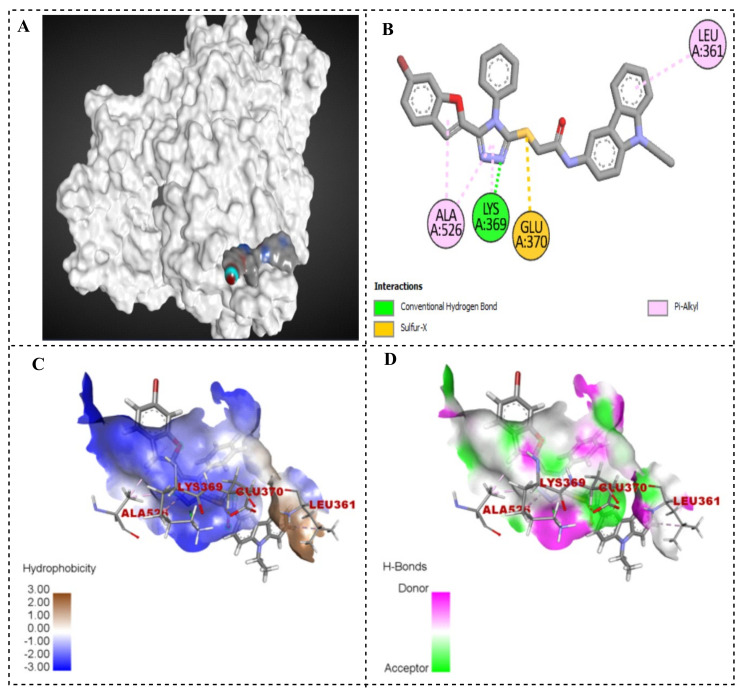
Interaction modes of compound **9j**: (**A**) Calculated surface of docked **9j** with the RNA-dependent RNA-polymerase, (**B**) 2D representation, (**C**) Hydrophobic interface, (**D**) Hydrogen bonding interface.

**Figure 23:**
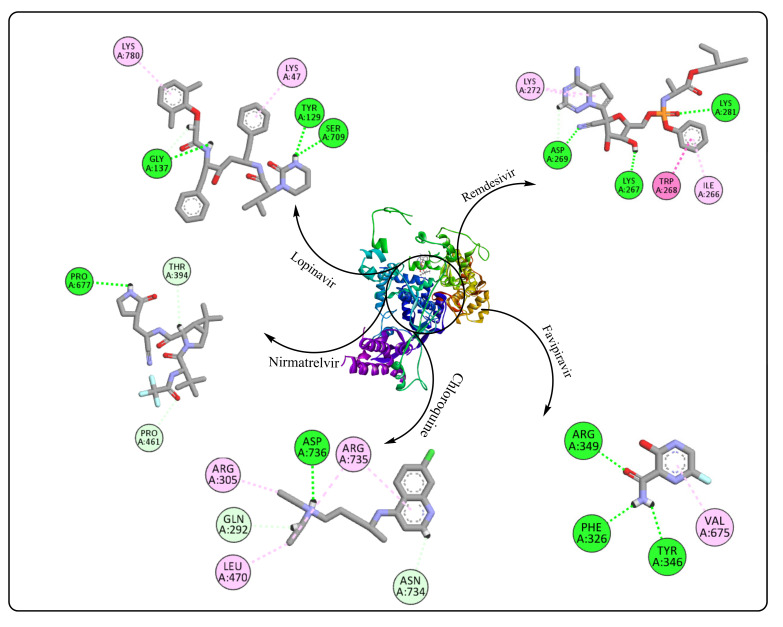
Two-dimensional diagram of standard drugs after binding with the RNA-dependent RNA polymerase.

RdRp accounts for the central machinery for the coronaviral genome’s transcription and replication, making it an appealing target for anti-viral drugs. In order to find potent inhibitors of coronavirus, the series of carbazole derivatives was docked with the RdRp protein (PDB Id: 6M71). Table 3 contains the molecular docking, binding scores, and interaction analysis of the potent compounds with RNA-dependent RNA-polymerase along with the standards compared with five different standards. The derivatives **9e**, **9g**, **9h**, **9i**, and **9j** were found to be potent, as exhibited by their strong binding energies, i.e., −7.61, −7.63, −8.10, −8.01, and −7.54 Kcal/mol, respectively, (Figure 18, Figure 19, Figure 20, Figure 21 and Figure 22). The highest binding score of −8.10 Kcal/mol exhibited by **9h** indicated the formation of six pi–donor and carbon–hydrogen bonds with THR24 (dHB = 2.63 Å), PHE96 (dHB = 2.76 Å), PRO677 (dHB = 5.08 Å), LEU760 (dHB = 2.61 Å), ASN458 (dHB = 2.42 Å), and GLU550 (dHB = 2.55 Å) (Figure 18). Besides these interactions, pi–alkyl interactions were also formed between the pi group of the benzene ring and the residues VAL675, PRO523, PRO461, and ARG549. The ligand interactions of **9i** indicated the development of a hydrogen bond between the -NH of the derivatives and ASP760 (dHB = 2.25 Å) (Figure 19). The formation of carbon–HB took place between the hydrogen of the ethyl group and the carbon of ASP761 at a distance of 2.86 Å. The development of a pi–donor hydrogen bond between the pi electrons of the furan ring and ARG555 was located at 3.15 Å. Besides these, two pi–alkyl interactions were formed between the pi electrons of benzene rings and LYS798 (5.27 Å) and VAL557 (5.43 Å). The interaction analysis of **9g** (−7.63 Kcal/mol) indicated the formation of one conventional hydrogen bond between the -NH group of the derivatives and the carbonyl group of SER861 (dHB = 2.05 Å) (Figure 20). The pi electrons of residue PHE594 furnished three π–π T-shaped interactions with the carbazole ring at distances of 4.97 Å, 4.41 Å, and 4.86 Å. Other pi–alkyl interactions were forged between the π electrons of the benzene ring and the alkyl group of ILE864. The triazole ring developed two pi–alkyl interactions with the residues LEU862 and ALA840. The residue ALA840 also conferred a pi–alkyl interface with the phenyl group at 5.34 Å. The alkyl groups of amino acid residue ILE548 conferred a bifurcated pi–alkyl connection with the pi groups of the phenyl ring at 5.08 Å and furan ring at 5.31 Å. Another pi–alkyl interaction was forged between the alkyl group of ALA547 and the phenyl ring of benzofuran at the longest distance of 5.37 Å. The interaction details of **9e** indicated the formation of a conventional hydrogen bond between the carbonyl group of the derivatives at 2.60 Å and the phenyl ring of carbazole at 3.02 Å by the same amino acid residue LYS621 (Figure 21). A pi–lone pair interaction developed between the pi groups of the derivatives and ARG555 at 2.84 Å. The chloro group of the residue developed three pi–alkyl interactions with ILE548 at 4.46 Å, ARG836 at 4.48 Å, and HIS439 at 4.15 Å, respectively. The residue ALA550 developed a pi–alkyl interaction with the Ph group of the derivatives at 4.27 Å. The residue VAL166 forged a three-way pi–alkyl interface with the two Ph rings of carbazole at 4.64 and 4.88 Å and the furan ring at 4.04 Å. It also developed an alkyl interaction with the alkyl group of carbazole at 4.02 Å. The residue PRO620 carved pi–alkyl coupling with the phenyl ring of the carbazole derivatives at 5.14 Å. Similarly, the binding of **9j** with the RdRp protein indicated the establishment of a hydrogen bond between the -N- of the triazole functionality and LYS369 at 2.03 Å (Figure 22). The same residue developed a pi–alkyl interaction with the pi groups of the triazole ring at 4.99 Å. The residue ALA526 forged a bifurcated interaction with the triazole ring at 4.68 Å and furan ring at 4.80 Å. The residue LEU361 presented pi–alkyl mapping with the pi group of carbazole at 4.96 Å. Similarly, a sulfur–halogen bond developed between the sulfur atom of the derivative and the halogen group of GLU370 at 3.22 Å. Figure 23 contains the two-dimensional images of the standards docked with RdRp. The docking interaction images of **9a**, **9b**, **9c**, **9d**, and **9f** are given in the (Supplementary Information Figures S28–S32).

Additionally, the interaction of selected protein targets was also elaborated by performing molecular docking against some other therapeutic agents, i.e., molnupiravir, nelfinavir, and ivermectin, to evaluate their efficacy against COVID-19 (Table 4). Molnupiravir is an important drug that exhibits significant activity against RNA-dependent RNA-polymerase (RdRp), with a binding score of −5.78 Kcal/mol [86]. The anti-COVID-19 drug, nelfinavir, exhibits crucial cytotoxicity as a protease and spike glycoprotein inhibitor (−7.36 and −6.50 Kcal/mol, respectively) [87]. Similarly, ivermectin, displayed notable binding interactions with all three targets, i.e., −8.61, −8.33, and −6.93 Kcal/mol against Mpro, the spike glycoprotein, and RdRp, respectively [88,89]. The binding scores of nelfinavir and ivermectin against the main protease indicated that the synthesized compounds had greater binding scores than nelfinavir and were comparable with ivermectin. The binding scores of nelfinavir and ivermectin with the spike glycoprotein denoted that the synthesized compounds were more potent than nelfinavir but less than ivermectin. Similarly, the binding interactions of molnupiravir and ivermectin with RdRp suggested that the compounds exhibited better binding affinities.

### 2.4. ADMET Analysis

The ADMET (Adsorption, Distribution, Metabolism, Excretion, and Toxicity) properties are considered key points in the process of the discovery of drugs and their development (Table 5). A good quality drug should possess significant potency against the target and a suitable ADMET profile at a required therapeutic dose. This study selected four potent compounds among the series to perform ADMET analysis to assess their pharmacokinetic and toxicity properties. All of the compounds possessed a suitable range of physiochemical properties involving the number of HB donors and acceptors, with their log S, log D, and TPSA (topological polar surface area) values falling in the optimum range corresponding to the solubility and interaction of the target compounds in the lipid environment. The selected medicinal properties for elucidating the therapeutic potential of the target compounds involved QED (quantitative estimate of drug likeness), which was found to be in the complex region for targeted compounds. All of the compounds revealed no PAINs, BMS, and chelator rule violations in the current scenario. The subjected compounds violated one Lipinski rule while no Pfizer rule was violated, indicating good medicinal characteristics. In oral drug administration, one of the important ADMET features involves the movement of drugs across the intestinal barrier of the human body, which can be determined from the values of HIA (human intestinal absorption) and Caco-2 permeability, as exhibited in Table 5. All of the compounds, except **9h**, exhibited blood–brain barrier permeability close to the optimal value, suggesting good absorption of the target compounds across the human body. Furthermore, the interaction of the selected compounds as substrates or inhibitors to Pgp (P-glycoprotein) was also a critical determinant for the evaluation of pharmacokinetic properties. The target compounds were found to exhibit potential as Pgp inhibitors, as represented in the table. The distribution factor of compounds was assessed via the values of BBB (Blood–brain barrier) and PPB (plasma protein binding). The compound **9e** displayed a better output value of 0.999 to act as BBB+. The four compounds subjected to ADMET analysis inhibited CYP enzyme activity, except **9h,** suggesting that these compounds were suitable choices for drug development. The toxic value profile of the four compounds suggested the DILI (drug-induced liver injury) value to be close to toxic while the acute toxicity rule was 0 for all. These compounds were found to be hERG blockers and AMES mutagenic, exhibiting the potential to be modified into potent compounds. Overall, the ADMET profiles of the selected compounds indicated that, after the addition of a few modifications to the structures of the target compounds, these can be developed as potent compounds.

## 3. Experimental

The chemicals (9-ethyl-9H-carbazol-3-amine, substituted salicylaldehydes, ethyl chloroacetate, bromoacetyl bromide, sodium hydroxide, potassium carbonate, dichloromethane, and dimethyl formamide) involved in the synthetic procedure were obtained from Macklin, Daejung, and/or Alfa Aesar. Additionally, sodium sulfate, ethyl acetate, n-hexane, and ethanol were obtained from local suppliers for carrying out the purification process. All employed chemicals were purified and dried before use. All of the synthesized compounds were characterized by NMR spectroscopy using Bruker 400 and 600 spectrometers in CDCl_3_ or DMSO-d_6_. The chemical shift values are reported in downfield ppm using TMS (tetramethyl silane) as the internal reference. The coupling constants are given in Hz. The melting points of the newly synthesized compounds were determined using the Gallenkamp equipment apparatus.

### 3.1. General Synthetic Protocol for the Derivatives ***9a***–***j***

To obtain carbazole-based benzofuran-1,2,4-triazoles **9a**–**j**, the solution of benzofuran triazole (1 mmol) in dichloromethane (5 mL) was stirred with K_2_CO_3_ (1.5 mmol) and KI (0.18 mmol). Next, 2-bromo-*N*-(9-ethyl-9*H*-carbazol-3-yl)acetamide (1.1 mmol) was added to RBF and the reaction mixture was stirred overnight at room temperature. After monitoring the reaction progress with TLC (thin layer chromatography), n-hexane and distilled water were added to obtain precipitates. The pure product was obtained after silica gel-mediated chromatographic purification.

**2-((5-(benzofuran-2-yl)-4-phenyl-4** ***H*-1,2,4-triazol-3-yl)thio)-*N*-(9-ethyl-9*H*-carbazol-3-yl)acetamide (9a)**

Light grey powder, m.p. = 207 °C, 70%; δ _H_ (400 MHz, CDCl_3_) δ 10.32 (s, 1H), 8.47 (s, 1H), 8.06 (d, *J* = 8 Hz, 1H), 7.63 (d, *J* = 4 Hz, 1H), 7.59 (d, *J* = 8 Hz, 2H), 7.43 (t, *J* = 8 Hz, 3H), 7.36 (d, *J* = 8 Hz, 3H), 7.18 (dt, *J* = 8 Hz, 2H), 6.46 (s, 1H), 4.29 (q, *J* = 8 Hz, 2H), 4.15 (s, 2H), 2.57 (s, 3H), 1.36 (t, *J* = 8 Hz, 3H). δ _C_ NMR (101 MHz, CDCl_3_) δ 165.81, 154.89, 154.65, 148.03, 141.82, 140.35, 137.09, 132.65, 131.24, 130.42, 130.42, 130.42, 130.18, 127.34, 127.34, 127.10, 126.48, 125.68, 123.71, 122.86, 121.85, 120.86, 119.08, 118.62, 112.21, 111.79, 108.41, 108.37, 108.32, 37.53, 36.47, 13.77; MS, (*m*/*z*): [M^+^ + 1] 544.2.

**2-((5-(5-bromobenzofuran-2-yl)-4-phenyl-4** ***H*-1,2,4-triazol-3-yl)thio)-*N*-(9-ethyl-9*H*-carbazol-3-yl)acetamide (9b)**

Cream-colored powder, m.p. = 235 °C, 65%; δ _H_ (400 MHz, CDCl_3_) 10.25 (1 H, s), 8.46 (1 H, s), 8.06 (1 H, d, *J* = 8 Hz), 7.67–7.59 (3 H, m), 7.58 (2 H, d, *J* = 8 Hz), 7.41 (1 H, d, *J* = 8 Hz), 7.39–7.33 (4 H, m), 7.30–7.25 (2 H, m), 7.16 (1 H, t, *J* = 8 Hz), 6.34 (1 H, s), 4.29 (2 H, q, *J* = 8 Hz), 4.12 (2 H, s), 1.36 (3 H, t, *J* = 8 Hz). δ _C_ (101 MHz, CDCl_3_) 165.98, 155.00, 153.69, 147.83, 143.45, 140.50, 137.23, 132.77, 131.41, 130.60, 130.60, 130.32, 129.44, 129.23, 127.42, 127.42, 125.86, 124.52, 123.04, 122.99, 120.98, 119.18, 118.79, 116.91, 113.35, 112.32, 108.57, 108.51, 107.22, 37.68, 36.54, 13.92; MS, (*m*/*z*): [M^+^ + 2] 623.8.

***N*-(9-ethyl-9*H*-carbazol-3-yl)-2-((5-(naphtho[2,1-b]furan-2-yl)-4-phenyl-4*H*-1,2,4-triazol-3-yl)thio)acetamide (9c)**

Light green powder, m.p. = 164 °C, 72%; δ _H_ (400 MHz, CDCl_3_) 10.32 (1 H, s), 8.47 (1 H, d, *J* = 12 Hz), 8.08 (1 H, d, *J* = 8 Hz), 7.87 (2 H, dd, *J* = 8 Hz), 7.73 (1 H, d, *J* = 8 Hz), 7.69–7.59 (4 H, m), 7.50 (3 H, t, *J* = 8 Hz), 7.40 (3 H, t, *J* = 4 Hz), 7.34 (2 H, d, *J* = 8 Hz), 7.27 (1 H, d, *J* = 8 Hz), 6.92 (1 H, s), 4.27 (2 H, p, *J* = 8 Hz), 4.08 (2 H, d, *J* = 8 Hz), 1.36 (3 H, t, *J* = 8 Hz). δ _C_ (101 MHz, CDCl_3_) 166.21, 154.40, 153.04, 141.82, 140.51, 137.22, 133.13, 131.22, 130.60, 130.51, 130.51, 130.51, 130.40, 129.36, 129.04, 127.69, 127.56, 127.56, 127.56, 127.01, 127.01, 125.82, 125.33, 123.24, 121.01, 119.21, 118.77, 118.77, 112.43, 112.35, 108.55, 108.51, 106.91, 37.67, 36.53, 13.91; MS, (*m*/*z*): [M^+^ + 1] 594.2.

***N*-(9-ethyl-9*H*-carbazol-3-yl)-2-((5-(7-methoxybenzofuran-2-yl)-4-phenyl-4*H*-1,2,4-triazol-3-yl)thio)acetamide (9d)**

Grey powder; m.p. = 174 °C, 75%; δ _H_ (400 MHz, CDCl_3_) 10.38 (1 H, s), 8.49 (1 H, s), 8.07 (1 H, d, *J* = 8 Hz), 7.61 (4 H, dq, *J* = 8 Hz), 7.42 (1 H, s), 7.39–7.35 (3 H, m), 7.28 (1 H, d, *J* = 8 Hz), 7.17 (1 H, d, *J* = 8 Hz), 7.10 (1 H, d, *J* = 8 Hz), 7.05 (1 H, d, *J* = 8 Hz), 6.79 (1 H, d, *J* = 8 Hz), 6.65 (1 H, s), 4.30 (2 H, q, *J* = 8 Hz), 4.21 (2 H, s), 3.83 (3 H, s), 1.37 (3 H, t, *J* = 8 Hz). δ _C_ (101 MHz, CDCl_3_) 165.77, 154.79, 145.72, 144.83, 141.70, 140.52, 137.27, 132.70, 131.33, 130.52, 130.52, 130.52, 130.52, 130.34, 129.04, 127.47, 127.47, 127.47, 125.82, 124.67, 123.03, 121.04, 119.31, 118.78, 114.10, 112.42, 109.51, 109.37, 108.54, 56.52, 37.69, 36.65, 13.93; MS, (*m*/*z*): [M^+^] 573.9.

**2-((5-(5-chlorobenzofuran-2-yl)-4-phenyl-4** ***H*-1,2,4-triazol-3-yl)thio)-*N*-(9-ethyl-9*H*-carbazol-3-yl)acetamide (9e)**

Light green powder, m.p. = 190 °C, 69%; δ _H_ (400 MHz, CDCl_3_) 10.27 (1 H, s), 8.46 (1 H, s), 8.05 (1 H, d, *J* = 8 Hz), 7.62 (4 H, dd, *J* = 8 Hz), 7.41 (2 H, s), 7.35 (5 H, t, *J* = 8 Hz), 7.26 (1 H, s), 7.16 (1 H, t, *J* = 8 Hz), 6.38 (1 H, s), 4.29 (2 H, q, *J* = 8 Hz), 4.16 (2 H, s), 1.36 (3 H, t, *J* = 8 Hz).δ _C_ (101 MHz, CDCl_3_) 165.83, 153.35, 147.75, 140.50, 137.24, 132.66, 131.51, 130.63, 130.63, 130.63, 130.63, 130.31, 129.53, 128.56, 127.43, 127.43, 126.88, 125.85, 123.04, 122.99, 121.48, 120.99, 119.20, 118.79, 112.93, 112.34, 108.57, 108.50, 107.70, 37.68, 36.61, 13.92; MS, (*m*/*z*): [M^+^] 577.9.

***N*-(9-ethyl-9*H*-carbazol-3-yl)-2-((5-(6-methoxybenzofuran-2-yl)-4-phenyl-4*H*-1,2,4-triazol-3-yl)thio)acetamide (9f)**

Cream-colored powder, m.p. = 207 °C, 60%; δ _H_ (600 MHz, DMSO-*D*_6_) 10.41 (1 H, s), 8.36 (1 H, s), 8.02 (1 H, d, *J* = 6 Hz), 7.68–7.61 (3 H, m), 7.59–7.49 (5 H, m), 7.44–7.38 (2 H, m), 7.15–7.11 (2 H, m), 6.83 (1 H, dd, *J* = 6 Hz), 6.29 (1 H, s), 4.37 (2 H, q, *J* = 6 Hz), 4.23 (2 H, s), 3.74 (3 H, s), 1.25 (3 H, t, *J* = 6 Hz). δ _C_ (151 MHz, DMSO-*D*_6_) 164.97, 159.02, 155.37, 152.18, 147.36, 141.81, 140.01, 136.34, 133.24, 130.88, 130.76, 130.30, 130.30, 130.30, 127.75, 127.75, 127.75, 125.87, 122.31, 122.01, 121.88, 120.15, 118.92, 118.64, 113.19, 111.32, 109.24, 109.16, 107.06, 95.66, 55.71, 37.02, 13.75; MS, (*m*/*z*): [M^+^-C_2_H_5_] 544.2.

***N*-(9-ethyl-9*H*-carbazol-3-yl)-2-((5-(7-methylbenzofuran-2-yl)-4-phenyl-4*H*-1,2,4-triazol-3-yl)thio)acetamide (9g)**

Light yellow powder, m.p. = 223 °C, 70%; δ _H_ (600 MHz, DMSO-*D*_6_) 10.39 (1 H, s), 8.36 (1 H, s), 8.02 (1 H, d, *J* = 6 Hz), 7.66–7.60 (3 H, m), 7.58 (2 H, d, *J* = 12 Hz), 7.55–7.50 (3 H, m), 7.42–7.38 (2 H, m), 7.13 (1 H, t, *J* = 6 Hz), 7.10 (2 H, d, *J* = 6 Hz), 6.80 (1 H, s), 4.37 (2 H, q, *J* = 6 Hz), 4.25 (2 H, s), 2.16 (3 H, s), 1.25 (3 H, t, *J* = 6 Hz). δ _C_ (151 MHz, DMSO-*D*_6_) 164.96, 153.08, 152.37, 147.36, 142.71, 140.02, 136.36, 133.52, 130.74, 130.61, 129.99, 129.99, 127.82, 127.82, 126.68, 126.55, 125.89, 123.84, 122.02, 121.90, 121.04, 120.26, 119.50, 118.94, 118.65, 111.35, 109.24, 109.16, 107.35, 37.12, 37.02, 14.24, 13.74; MS, (*m*/*z*): [M^+^] 557.0.

**2-((5-(6-(dimethylamino)benzofuran-2-yl)-4-phenyl-4** ***H*-1,2,4-triazol-3-yl)thio)-*N*-(9-ethyl-9*H*-carbazol-3-yl)acetamide (9h)**

Dark gray powder, m.p. = 137 °C, 63%; δ _H_ (600 MHz, DMSO-*D*_6_) 10.36 (1 H, s), 8.35 (1 H, s), 8.02 (1 H, d, *J* = 6 Hz), 7.67–7.60 (3 H, m), 7.53 (5 H, dd, *J* = 12 Hz), 7.40 (1 H, t, *J* = 6 Hz), 7.26 (1 H, d, *J* = 12 Hz), 7.13 (1 H, t, *J* = 6 Hz), 6.67 (1 H, s), 6.62 (1 H, d, *J* = 12 Hz), 6.09 (1 H, s), 4.37 (2 H, q, *J* = 6 Hz), 4.21 (2 H, s), 1.25 (3 H, t, *J* = 6 Hz), 1.03 (6 H, t, *J* = 6 Hz). δ _C_ (151 MHz, DMSO-*D*_6_) 165.02, 156.80, 151.61, 147.76, 147.31, 140.02, 139.61, 136.36, 133.41, 130.81, 130.74, 130.29, 130.29, 127.82, 127.82, 125.89, 122.12, 122.02, 121.89, 120.27, 118.93, 118.65, 115.69, 111.34, 110.14, 109.24, 109.16, 107.18, 92.67, 44.16, 37.06, 37.02, 13.75, 12.34; MS, (*m*/*z*): [M^+^ + 1] 587.2.

**2-((5-(7-ethoxybenzofuran-2-yl)-4-phenyl-4** ***H*-1,2,4-triazol-3-yl)thio)-*N*-(9-ethyl-9*H*-carbazol-3-yl)acetamide (9i)**

Light green powder, m.p. = 205 °C, 70%; δ _H_ (600 MHz, DMSO-*D*_6_) 10.39 (1 H, s), 8.35 (1 H, s), 8.02 (1 H, d, *J* = 6 Hz), 7.62 (3 H, d, *J* = 6 Hz), 7.57 (2 H, d, *J* = 6 Hz), 7.53 (3 H, d, *J* = 12 Hz), 7.40 (1 H, s), 7.11 (3 H, d, *J* = 18 Hz), 6.89 (1 H, s), 6.50 (1 H, s), 4.37 (2 H, d, *J* = 6 Hz), 4.25 (2 H, s), 4.04 (2 H, d, *J* = 6 Hz), 1.26 (6 H, dd, *J* = 6 Hz). δ _C_ (151 MHz, DMSO-*D*_6_) 165.45, 153.02, 147.70, 144.65, 144.02, 143.23, 140.52, 136.86, 133.75, 131.29, 131.22, 130.70, 130.70, 129.29, 128.26, 128.26, 126.38, 125.09, 122.51, 122.39, 120.76, 119.43, 119.14, 114.37, 111.84, 110.24, 109.73, 109.65, 107.77, 64.80, 37.57, 37.51, 15.23, 14.23; MS, (*m*/*z*): [M^+^ + 2] 589.0.

**2-((5-(6-bromobenzofuran-2-yl)-4-phenyl-4** ***H*-1,2,4-triazol-3-yl)thio)-*N*-(9-ethyl-9H-carbazol-3-yl)acetamide (9j)**

Light green powder, m.p. = 202 °C, 89%; δ _H_ (600 MHz, DMSO-*D*_6_) 10.35 (1 H, d, *J* 48.4), 8.35 (2 H, d, *J* = 6 Hz), 8.01 (1 H, d, *J* = 6 Hz), 7.67–7.62 (1 H, m), 7.58 (1 H, s), 7.56–7.51 (4 H, m), 7.44 (1 H, d, *J* = 12 Hz), 7.40 (3 H, t, *J* = 6 Hz), 7.18–7.11 (2 H, m), 6.80 (1 H, t, *J* = 6 Hz), 4.37 (2 H, q, *J* = 6 Hz), 4.26 (1 H, s), 4.02 (1 H, s), 1.25 (3 H, t, *J* = 6 Hz). δ _C_ (151 MHz, DMSO-*D*_6_) 165.78, 165.42, 153.34, 152.63, 147.41, 146.28, 141.93, 140.51, 136.85, 132.79, 131.28, 130.83, 130.43, 129.14, 128.40, 128.20, 126.37, 124.16, 122.51, 122.38, 120.99, 120.75, 119.42, 119.14, 117.40, 115.12, 111.83, 109.74, 109.62, 107.35, 37.51, 14.24; MS (*m*/*z*): [M^+^ + 2] 623.9.

#### 3.1.1. Molecular Docking

The crystal structures of the viral proteins were downloaded from RCSB PDB (Protein Data Bank). The PDB IDs of the proteins used were the main protease (PDB ID: 6LU7) [90], spike glycoprotein (PDB ID: 6WPT) [91], and RNA-dependent RNA-polymerase (PDB ID: 6M71) [92]. The molecular docking of the synthesized compounds was performed usingMOE-2022 software [84]. BIOVIA Discovery Studio visualizer 2024 software was used for the interpretation and generation of 2D images and hydrophobic and hydrogen bond interactions [93]. The selection of these proteins was made according to their role in SARS-CoV-2 treatment.

#### 3.1.2. ADMET Studies

The potent compounds were employed for ADMET analysis. The scores were obtained from the ADMETLAB 3.0. online server and were run for the evaluation of ADMET properties [94]. The qualitative assessment of ADMET (Adsorption, Distribution, Metabolism, Excretion, and Toxicity) properties is summarized in Table 4.

## 4. Conclusions

In conclusion, a facile, efficient, and straightforward methodology was adopted for synthesizing a novel series of functionalized benzofuran-based carbazole derivatives (**9a**–**j**) in good to high yields (65–89%) as potent inhibitors against SARS-CoV-2. The structural analysis of the synthesized compounds was carried out by ^1^H-NMR, ^13^C-NMR, and mass spectrometry (MS). The docking studies of the complete series were conducted against three proteins of SARS-CoV-2, named as the main protease (PDB Id = 6LU7), spike glycoprotein (PDB Id = 6WPT), and RNA-dependent RNA-polymerase (PDB Id = 6M71). Five FDA-approved drugs (remdesivir, chloroquine, favipiravir, lopinavir, and nirmatrelvir) were selected as standards. Among the FDA-approved drugs, lopinavir exhibited a high binding affinity with the main protease and RNA-dependent RNA-polymerase, whereas in the case of the spike glycoprotein, remdesivir exhibited a higher binding score than the other standards employed. Among the series of synthesized derivatives, the best binding scores were exhibited by compounds **9e**, **9h**, **9i**, and **9j**, which were even better than the standards. Additionally, compounds **9b** and **9c** against the main protease, compounds **9b**, **9d**, and **9f** against the spike glycoprotein, and compound **9g** against RdRp exhibited high binding energies as well. The potent compounds among the series were also subjected to ADMET studies to evaluate their pharmacokinetic potential, which suggested that the compounds possessed good ADMET profiles. The obtained results highlight the dynamic multitarget potential of the synthesized compounds as anti-viral agents and provide a useful background for further investigations.

## Figures and Tables

**Table 1:** The docking details, binding scores, and interaction analysis of potent compounds with the main protease.

Sr. No.	Compound Id	Binding Score (Kcal/mol)	Types of Interactions	Amino Acid Residues
1-	**9b**	−8.83	VdW, Conventional HB, Sulfur–X, Pi–Sigma–Donor HB, Amide–Pi Stacked, Alkyl, Pi–Alkyl, Pi–Sulfur, Pi–Sigma	PRO168, LEU167, GLN189, MET169, LEU141, CYS145, ASN142, GLY143, THR25, THR26, HIS41, MET49
2-	**9c**	−8.92	Conventional HB, Sulfur–X, Pi–Donor HB, Pi–Alkyl, Pi–Sulfur	THR26, GLY143, HIS41, CYS145, MET165
3-	**9e**	−8.78	VdW, Conventional HB, Carbon–HB, Sulfur–X, Pi–Sigma, Amide–Pi Stacked, Alkyl, Pi–Sulfur, Pi–Alkyl	SER46, GLY143, ASN142, ASN142, MET165, THR25, HIS41, MET49, CYS145, PRO168
4-	**9h**	−8.76	VdW, Carbon–HB, Pi–Sigma, Amide–Pi Stacked, Pi–Sulfur, Alkyl, Pi–Alkyl, Sulfur–X	ASN142, LEU141, THR190, GLN192, GLU166, PRO168, LEU167, GLN189, MET165, MET49, HIS41, GLY143, THR26
5-	**9i**	−8.87	Conventional HB, Carbon–HB, Pi–Donor HB, Pi–Lone Pair, Pi–Alkyl, Pi–Sigma, π–π Stacked	ASN119, GLY143, HIS41, MET49, MET165, THR190, GLU166, GLN189, THR24
6-	**9j**	−8.85	Conventional HB, Sulfur–X, Pi–Donor HB, Pi–Sigma, Pi–Sulfur, Pi–Alkyl, Alkyl	LEU167, MET165, CYS145, GLY143, THR25, THR26, HIS41
7-	Remdesivir	−8.55	Conventional HB, Carbon–HB, Pi–Donor HB, Pi–Sulfur, π–π T-Shaped, Pi–Alkyl	MET165, GLU166, HIS163, ASN142, GLN189, HIS41, CYS145, MET49
8-	Lopinavir	−8.73	Conventional HB, Carbon–HB, Pi–Donor HB, Pi–Sulfur, π–π T-Shaped, Alkyl, Pi–Alkyl	HIS41, CYS145, MET49, LEU27, THR26, GLY143, GLN189, ASN142, GLU166, PRO168, THR190, MET165
9-	Chloroquine	−6.40	Pi–Sigma, Pi–Lone Pair, Pi–Alkyl, Alkyl	HIS41, MET165, PRO168, GLU166, CYS145, MET49, HIS163, PRO52
10-	Favipiravir	−4.48	Conventional HB, Carbon–HB, Halogen (Fluorine), Pi–Sulfur	ASP187, MET165, GLN189, MET49
11-	Nirmatrelvir	−8.38	Conventional HB, Carbon–HB, Halogen (Fluorine), Pi–Sigma, Pi–Alkyl, Alkyl	HIS41, MET49, GLN189, HIS163, HIS172, CYS145, HIS164, MET165, GLY143, GLU166, PRO168, THR190, ARG188

Abbreviations: HB = Hydrogen bond; VdW = Van der Waals.

**Table 2:** The docking details, binding scores, and interaction analysis of potent compounds with the spike glycoprotein.

Sr. No.	Compound Id	Binding Score (Kcal/mol)	Types of Interaction	Amino Acid Residues
1-	**9b**	−6.44	VdW, Conventional HB, Sulfur–X, Pi–Donor HB, Amide–Pi Stacked, Pi–Sulfur, Alkyl, Pi–Alkyl, Pi–Sigma	PRO168, LEU167, GLN189, MET165, LEU141, CYS145, ASN142, GLY143, THR25, THR26, HIS41, MET49
2-	**9d**	−6.39	Conventional HB, Carbon–HB, Pi–Alkyl, Pi–Sigma	ALA706, VAL705, GLU1072, LYS1073
3-	**9e**	−6.69	Conventional HB, Pi–Alkyl, Pi–Sigma	LYS1073, GLU1072, ALA706, VAL705
4-	**9f**	−6.41	Conventional HB, Carbon–HB, Pi–Alkyl	LYS1073, ALA706, VAL705, ASN1074
5-	**9h**	−6.54	Conventional HB, Sulfur–X, Pi–Donor HB, Pi–Sigma, Alkyl, Pi–Alkyl	LYS1073, ASN1074, GLU1072, VAL705, ALA706
6-	**9i**	−6.44	Pi–Donor HB, Pi–Sigma, Pi–Lone Pair, Alkyl, Pi–Alkyl	LYS1073, ASN1074, GLU1072, ALA713, PRO715, VAL705, ASN703
7-	**9j**	−6.56	Conventional HB, Pi–Alkyl, Pi–Sigma	ALA706, LYS1073, GLU1072
8-	Remdesivir	−6.26	Conventional HB, Carbon–HB, Amide–Pi Stacked, Pi–Alkyl	VAL705, ALA706, SER704, LYS1073, ASN1074, GLU1072
9-	Lopinavir	−6.09	Carbon–HB, Pi–Donor HB, Pi–Lone Pair, Alkyl, Pi–Alkyl	ASN703, VAL705, ALA713, GLU1072, ALA706,
10-	Chloroquine	−5.14	Alkyl, Pi–Alkyl	ILE1130, ALA1078, VAL1133, TRP1102
11-	Favipiravir	−3.92	Conventional HB, Carbon–HB	LYS529, GLN580, SER530, ARG328, ASN331

Abbreviations: HB = Hydrogen Bond; VdW = Van der Waals.

**Table 3:** The docking details, binding scores, and interaction analysis of potent compounds with RNA-dependent RNA-polymerase.

Sr. No.	Compound Id	Binding Score (Kcal/mol)	Types of Interactions	Amino Acids Residues
1-	**9e**	−7.61	Conventional HB, Pi–Alkyl, Pi–Sigma	LYS1073, GLU1072. VAL705, ALA706
2-	**9g**	−7.63	Conventional HB, Carbon–HB, Pi–Alkyl	ALA1070, ASN703, VAL705
3-	**9h**	−8.10	Conventional HB, Sulfur–X, Pi–Donor HB, Pi–Sigma, Alkyl, Pi–Alkyl	LYS1073, ASN1074, GLU1072. VAL705, ALA706
4-	**9i**	−8.01	Pi–Donor HB, Pi–Sigma, Pi–Lone Pair, Alkyl, Pi–Alkyl	LYS1073, ASN1074, GLU1072, VAL705, ALA713, PRO715, ASN703
5-	**9j**	−7.54	Conventional HB, Pi–Sigma, Pi–Alkyl	LYS1073, GLU1072, ALA706
6-	Remdesivir	−6.82	Conventional HB, Alkyl, Pi–Alkyl, Carbon–HB	LYS272, ASP269, LYS267, TRP268, ILE266, LYS281
7-	Lopinavir	−7.48	VdW, Conventional HB, Carbon–HB, Pi–Alkyl	LYS780, GLY137, ASN138, LYS47, TYR129, SER709, TYR32, ALA46, GLY774, THR710, ASP711, LYS714, CYS139, ASP140, GLU136, ASP135, THR141
8-	Chloroquine	−5.70	Conventional HB, Carbon–HB, Alkyl, Pi–Alkyl	ARG305, ASP736, ARG735, GLN292, LEU470, ASN734
9-	Favipiravir	−4.21	Conventional HB, Pi–Alkyl	ARG349, PHE326, TYR346, VAL675
10-	Nirmatrelvir	−6.41	Conventional HB, Carbon–HB	PRO677, THR394, PRO461

Abbreviations: HB = Hydrogen Bond; VdW = Van der Waals.

**Table 4:** Binding scores of molnupiravir, nelfinavir, and ivermectin with Mpro, the spike glycoprotein, and RdRp.

	Mpro (Kcal/mol)	Spike Glycoprotein (Kcal/mol)	RdRp (Kcal/mol)
Molnupiravir	-	-	−5.78
Nelfinavir	−7.36	−6.50	-
Ivermectin	−8.61	−8.33	−6.93

**Table 5:** ADMET scores of screened compounds.

Compound ID		9e	9h	9i	9j
**Physiochemical properties**	nHD	1.0	1.0	1.0	1.0
	nHA	7.0	8.0	8.0	7.0
	TPSA	77.88	81.12	87.11	77.88
	Log S	−7.563	−7.399	−7.274	−7.719
	Log D	4.5	4.558	4.622	4.728
**Medicinal Chemistry**	QED	0.193	0.169	0.173	0.181
	GASA	0.0	0.0	0.0	0.0
	MCE-18	36.0	37.0	36.0	36.0
	NP score	−1.918	−1.685	−1.744	−1.769
	Pfizer rule	0.0	0.0	0.0	0.0
	Lipinski rule	1.0	1.0	1.0	1.0
	PAINS	0 alert	0 alert	0 alert	0 alert
	Chelator rule	0 alert	0 alert	0 alert	0 alert
	BMS	0 alerts	0 alert	0 alert	0 alert
**Absorption**	Caco-2 permeability	−5.14	−5.194	−5.072	−5.151
	HIA	0.0	0.001	0.0	0.0
	PAMPA	0.08	0.135	0.064	0.069
	Pgp-substrate	0.0	0.022	0.001	0.0
	Pgp-inhibitor	0.994	0.954	0.992	0.994
**Distribution**	BBB	0.999	0.458	0.96	0.971
	PPB	98.262	98.294	97.673	97.761
**Metabolism**	CYP2C19 inhibitor	Yes	Yes	Yes	Yes
	CYP1A2 substrate	Yes	Yes	Yes	Yes
	CYP2D6 inhibitor	Yes	No	Yes	Yes
	CYP2C9 substrate	Yes	0.529	0.696	Yes
	HLM stability	0.964	0.961	0.87	0.865
**Excretion**	CL plasma	Low clearance	Low clearance	Low clearance	Low clearance
**Toxicity**	DILI	0.998	0.994	0.997	0.999
	hERG blockers	0.898	0.677	0.855	0.872
	AMES mutagenicity	0.827	0.921	0.887	0.803
	Rat oral acute toxicity rule	0.661	0.454	0.627	0.736
	Eye corrosion	0.0	0.0	0.0	0.0
	Acute toxicity rule	0	0	0	0

(TPSA = Topological Polar Surface Area; QED = Quantitative Estimate of Drug Likeness; MCE = Medicinal Chemistry Evaluation; NP = Natural Product; PAINS = Pan Assay Interference Compounds; HIA = Human Intestinal Absorption; PAMPA = Parallel Artificial Membrane Permeability Assay; BBB = Blood–Brain Barrier; PPB = Plasma Protein Binding; HLM = Human Liver Microsomes; DILI = Drug-Induced Liver Injury; hERG = human Ether-a-go-go-Related Gene).

## Data Availability

All the data are contained within the article and the Supplementary Materials.

## Supplementary Materials

The following supporting information can be downloaded at: https://www.mdpi.com/article/10.3390/ijms252312708/s1.
