# Supplementary material for: Design, Synthesis and Biological Exploration of Novel N-(9-Ethyl-9H-Carbazol-3-yl)Acetamide-Linked Benzofuran-1,2,4-Triazoles as Anti-SARS-CoV-2 Agents: Combined Wet/Dry Approach Targeting Main Protease (Mpro), Spike Glycoprotein and RdRp
Source: Int J Mol Sci. 2024 Nov 26;25(23):12708. doi: 10.3390/ijms252312708 (PMC11641759; doi:10.3390/ijms252312708)
Supplement: Supplementary file 1 [file ijms-25-12708-s001.zip › ijms-3295757-supplementary.pdf]

## Supplementary Information For

# Design, Synthesis and Biological Exploration of Novel *N*-(9-Ethyl-9*H*-Carbazol-3-yl)Acetamide Linked Benzofuran-1,2,4-Triazoles as Anti-SARS-CoV-2 Agents: Combined Wet/Dry Approach Targeting Main Protease ($M^{pro}$), Spike Glycoprotein and RNA Dependent RNA Polymerase (RdRp)

## Table of contents


**1- NMR Spectra of compounds 9a-j**

**Figure S1.** $^{1}$H NMR spectra of **9a** **3**

**Figure S2.** $^{13}$C NMR Spectra of **9a** **4**

**Figure S3.** $^{1}$H NMR spectra of **9b** **5**

**Figure S4.** $^{13}$C NMR Spectra of **9b** **6**

**Figure S5.** $^{1}$H NMR spectra of **9c** **7**

**Figure S6.** $^{13}$C NMR Spectra of **9c** **8**

**Figure S7.** $^{1}$H NMR spectra of **9d** **9**

**Figure S8.** $^{13}$C NMR Spectra of **9d** **10**

**Figure S9.** $^{1}$H NMR spectra of **9e** **11**

**Figure S10.** $^{13}$C NMR Spectra of **9e** **12**

**Figure S11.** $^{1}$H NMR spectra of **9f** **13**

**Figure S12.** $^{13}$C NMR Spectra of **9f** **14**

**Figure S13.** $^{1}$H NMR spectra of **9g** **15**

**Figure S14.** $^{13}$C NMR Spectra of **9g** **16**

**Figure S15.** $^{1}$H NMR spectra of **9h** **17**

**Figure S16.** $^{13}$C NMR Spectra of **9h** **18**

**Figure S17.** $^{1}$H NMR spectra of **9i** **19**

**Figure S18.** $^{13}$C NMR Spectra of **9i** **20**

**Figure S19.** $^{1}$H NMR spectra of **9j** **21**

**Figure S20.** $^{13}$C NMR Spectra of **9j** **22**

**2- Tables** 23

**3- Figures** 25

**Figure S21.** Interaction modes of compound **9a** with main protease **26**

**Figure S22.** Interaction modes of compound **9d** with main protease **27**

**Figure S23.** Interaction modes of compound **9f** with main protease **28**

**Figure S24.** Interaction modes of compound **9g** with main protease **29**

**Figure S25.** Interaction modes of compound **9a** with spike glycoprotein **30**

**Figure S26.** Interaction modes of compound **9c** with spike glycoprotein **31**

**Figure S27.** Interaction modes of compound **9g** with spike glycoprotein **32**

**Figure S28.** Interaction modes of compound **9a** with RdRp **33**

**Figure S29.** Interaction modes of compound **9b** with RdRp **34**

**Figure S30.** Interaction modes of compound **9c** with RdRp **35**

**Figure S31.** Interaction modes of compound **9d** with RdRp **36**

**Figure S32.** Interaction modes of compound **9f** with RdRp **37**

**Figure S33.** Interaction modes of compound **9g** with RdRp **38**

**Figure S34.** Binding interaction of nelfinavir and ivermectin with Mpro **39**

**Figure S35.** Binding interaction of nelfinavir and ivermectin with Spike glycoprotein **39**

**Figure S36.** Binding interaction of molnupiravir and ivermectin with RdRp **40**

## 1- NMR Spectra of compounds 9a-j

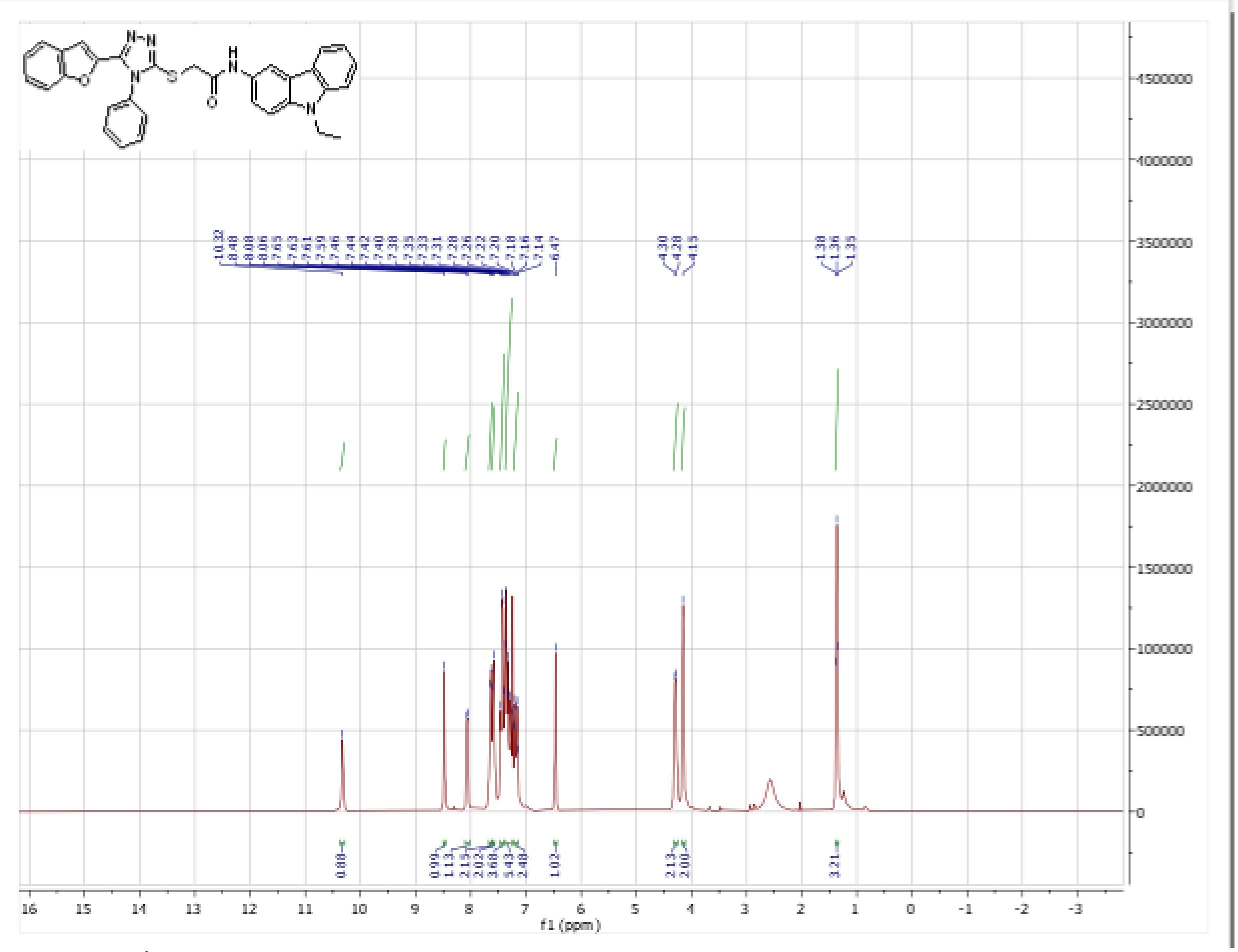


Figure S1: $^{1}$H NMR of compound **9a**.

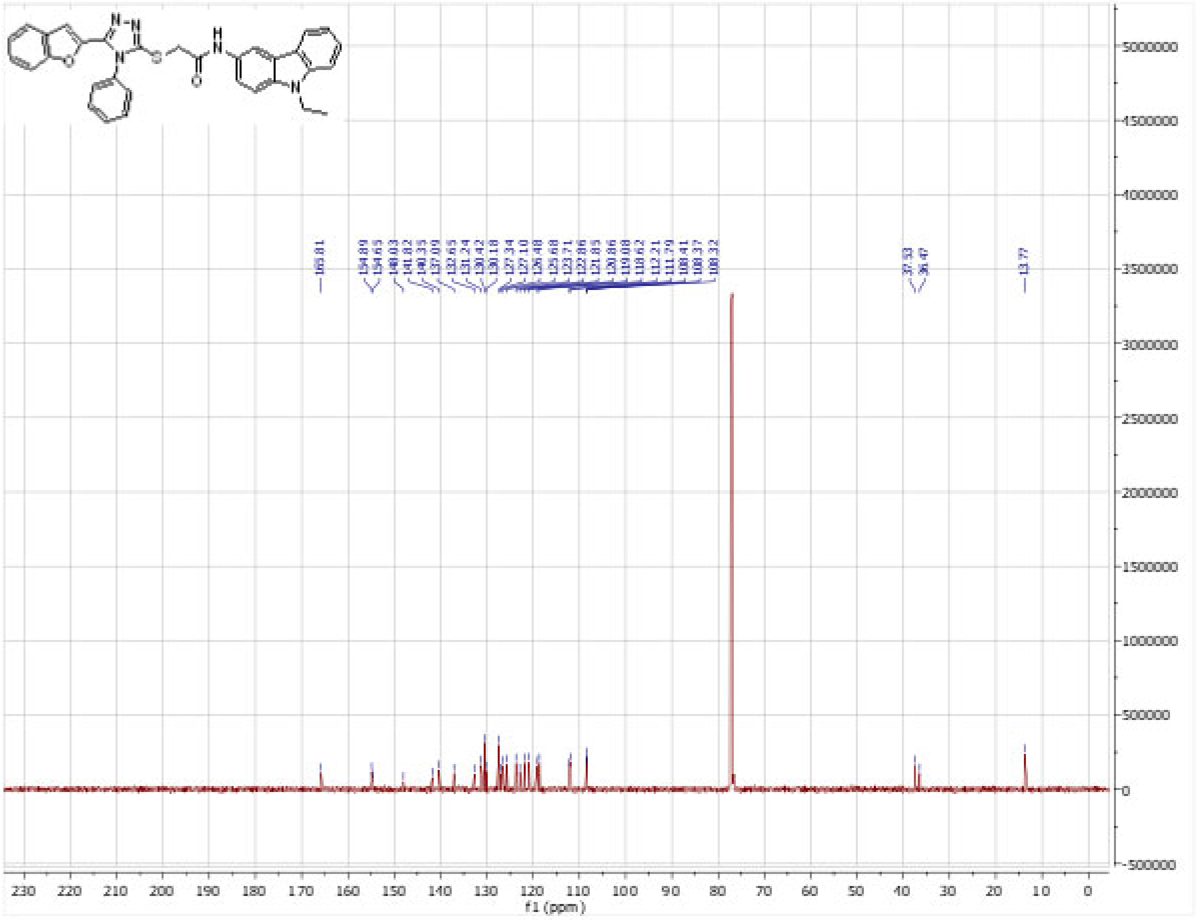


Figure S2: $^{13}$C NMR of compound **9a**.

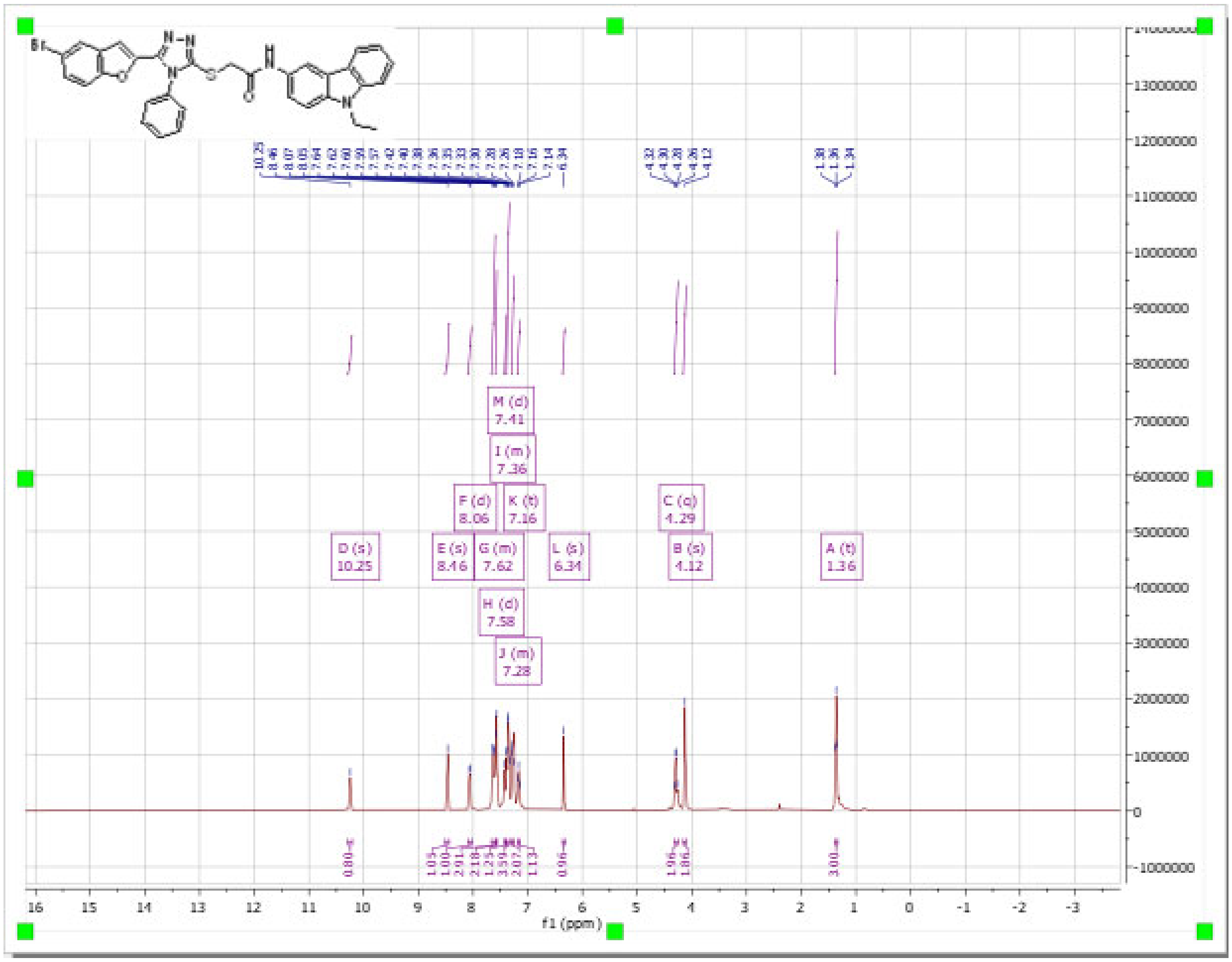

**Figure S3**: $^{1}$H NMR of compound **9b**.

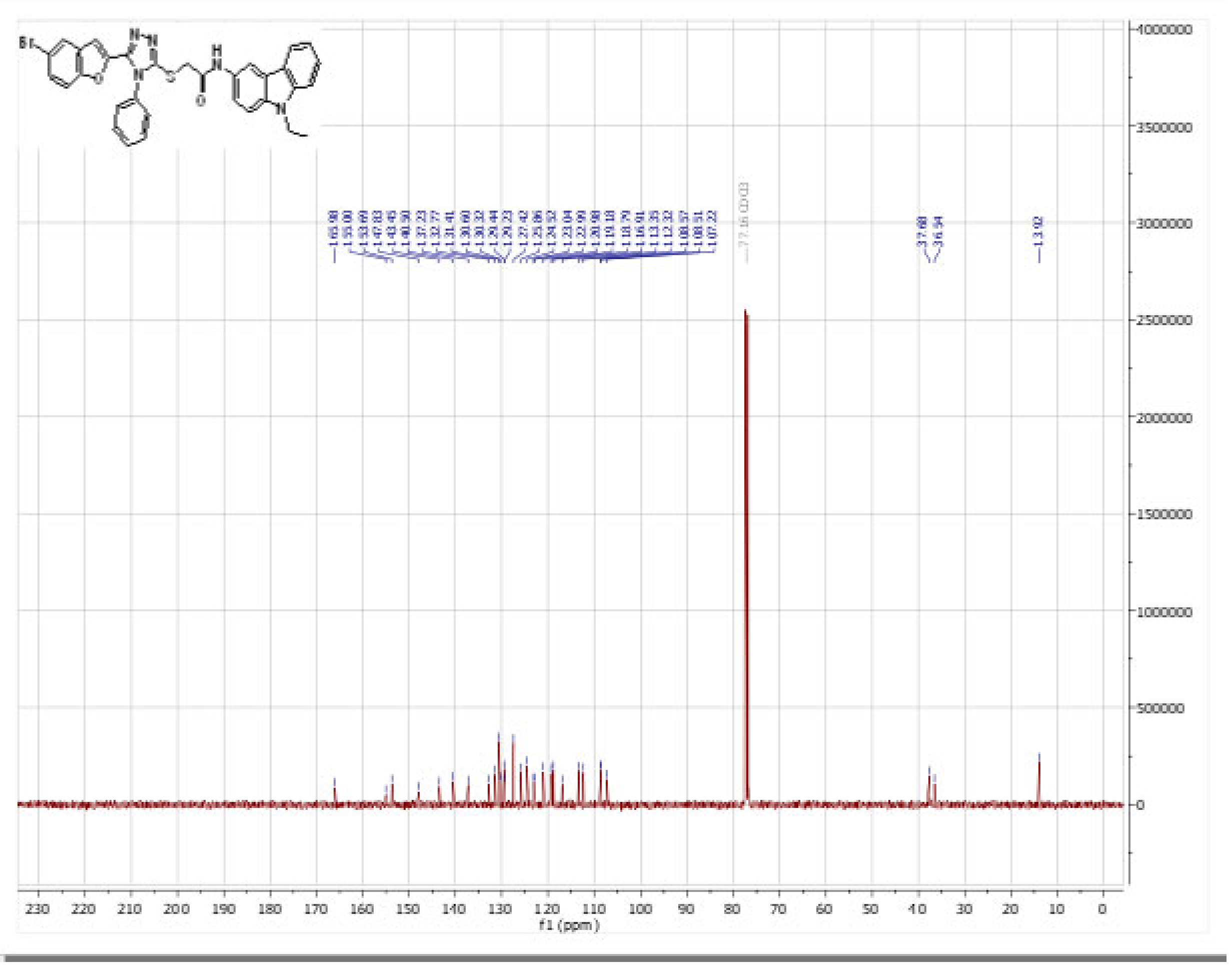


**Figure S4**: $^{13}$C NMR of compound **9b**.

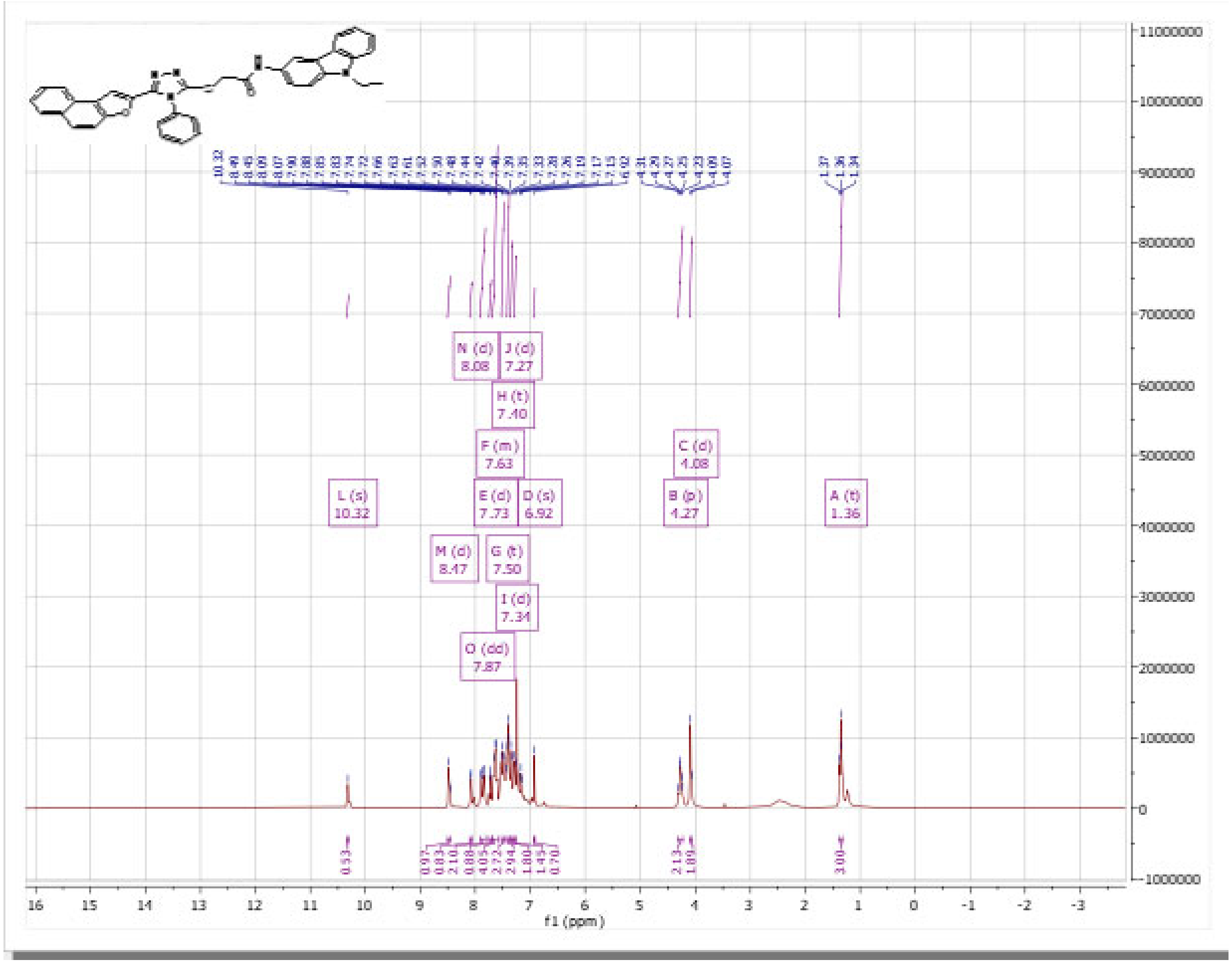


**Figure S5**: $^{1}$H NMR of compound **9c**.

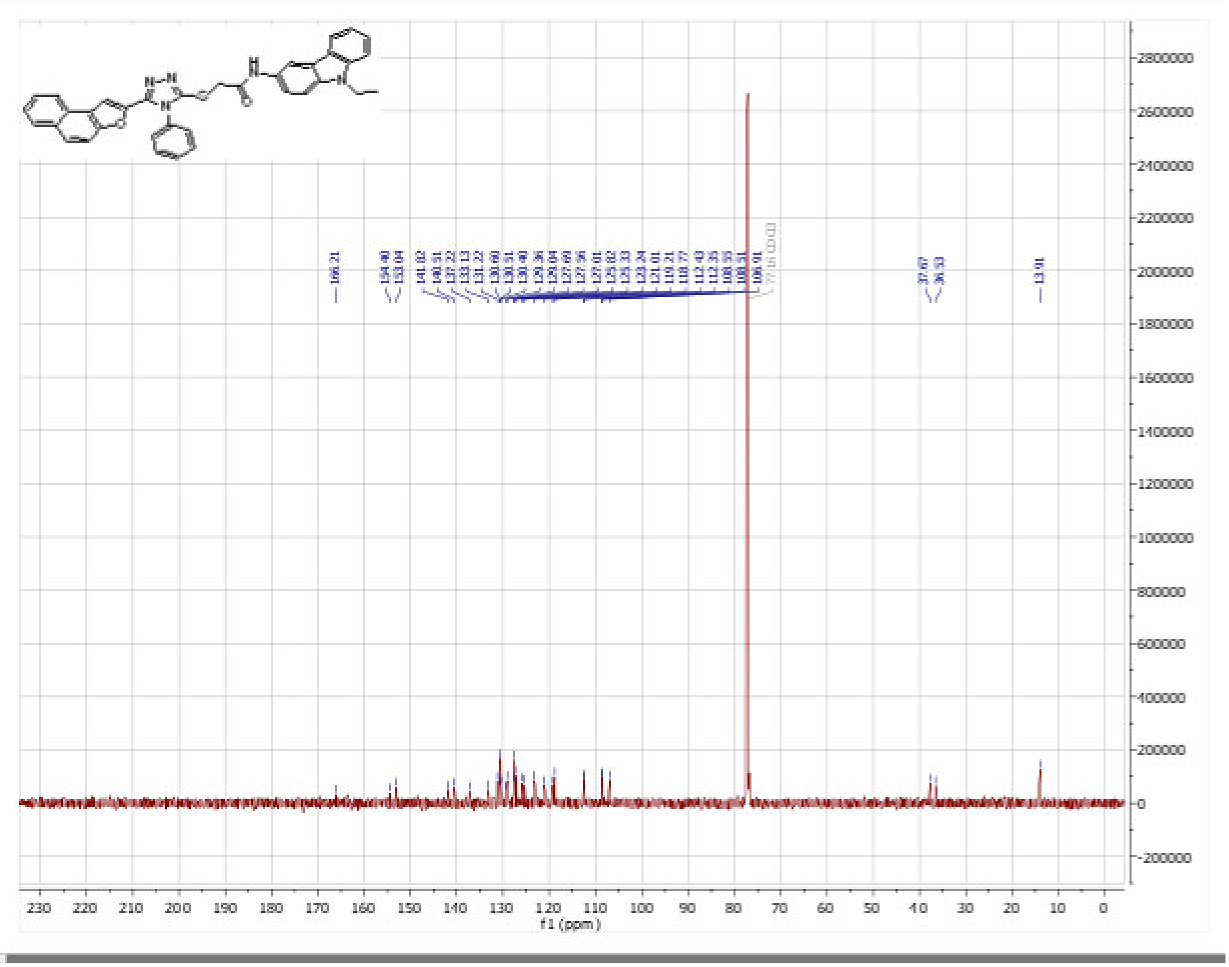

**Figure S6**: $^{13}$C NMR of compound **9c**.

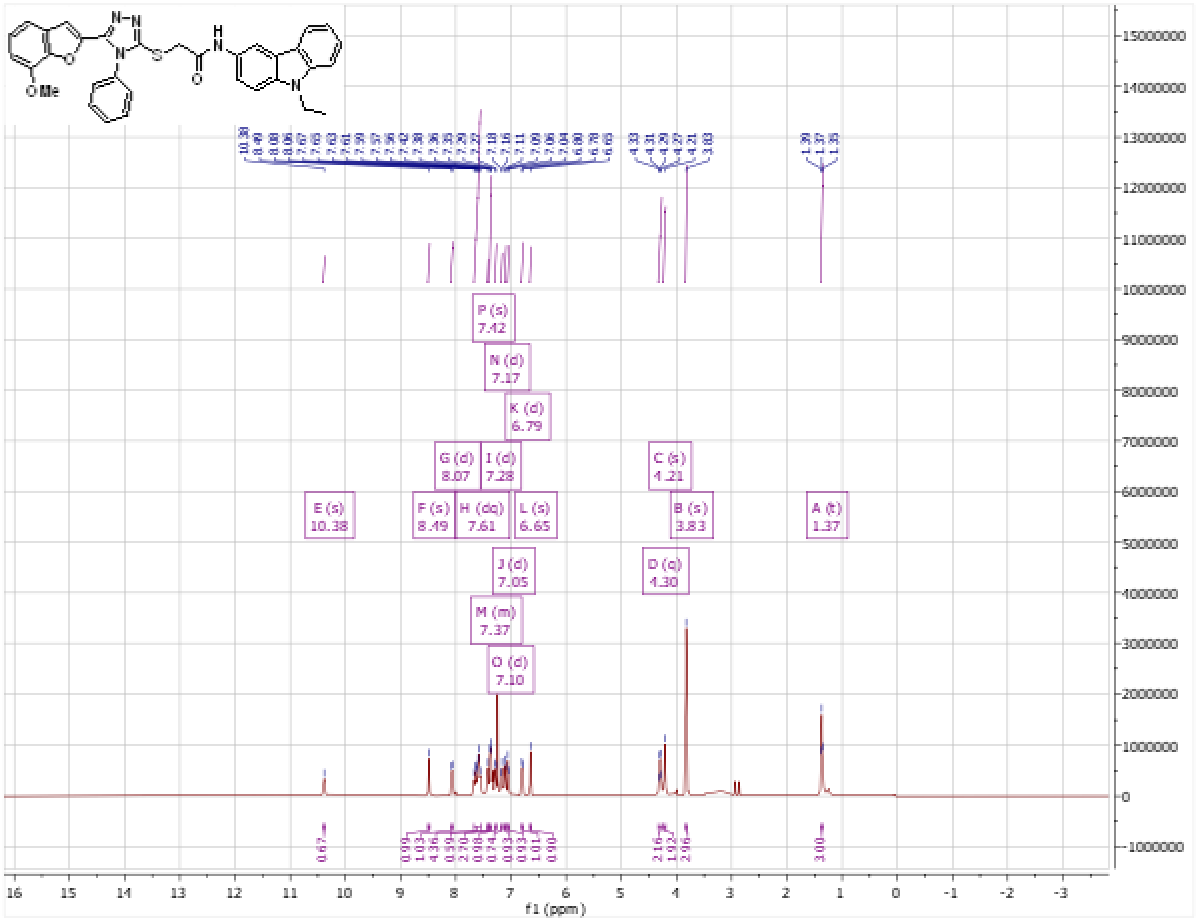

**Figure S7**: $^{1}$H NMR of compound **9d**.

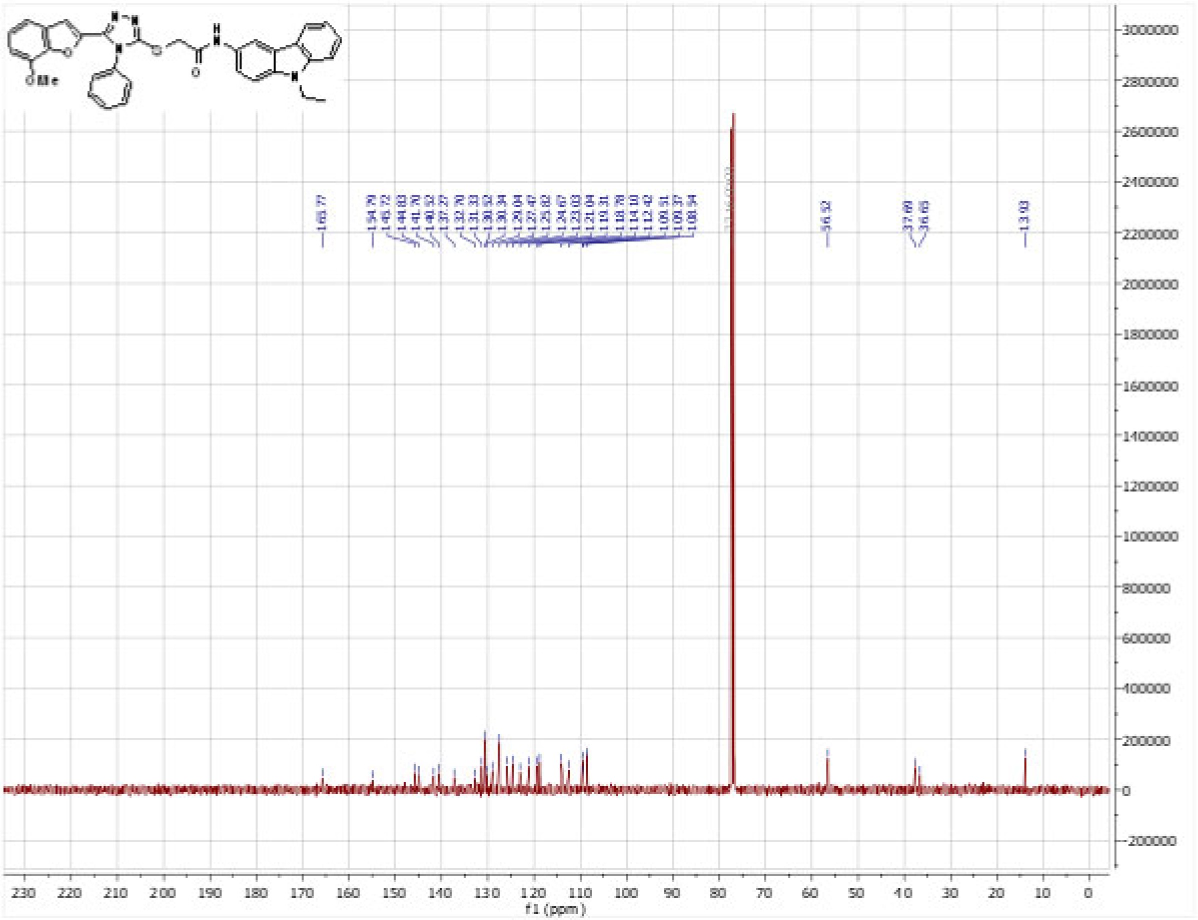


**Figure S8**: $^{13}$C NMR of compound **9d**.

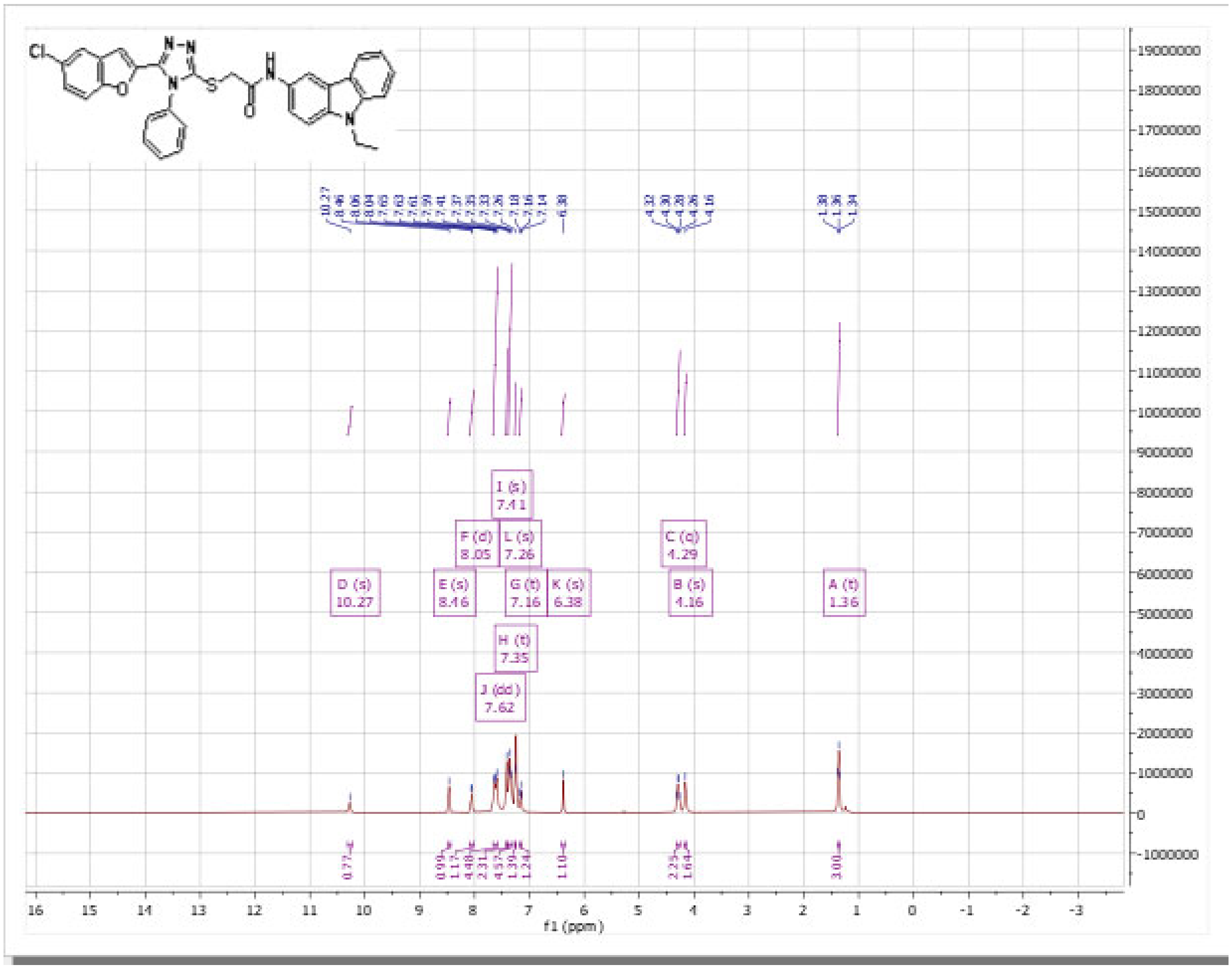

**Figure S9**: $^{1}$H NMR of compound **9e**.

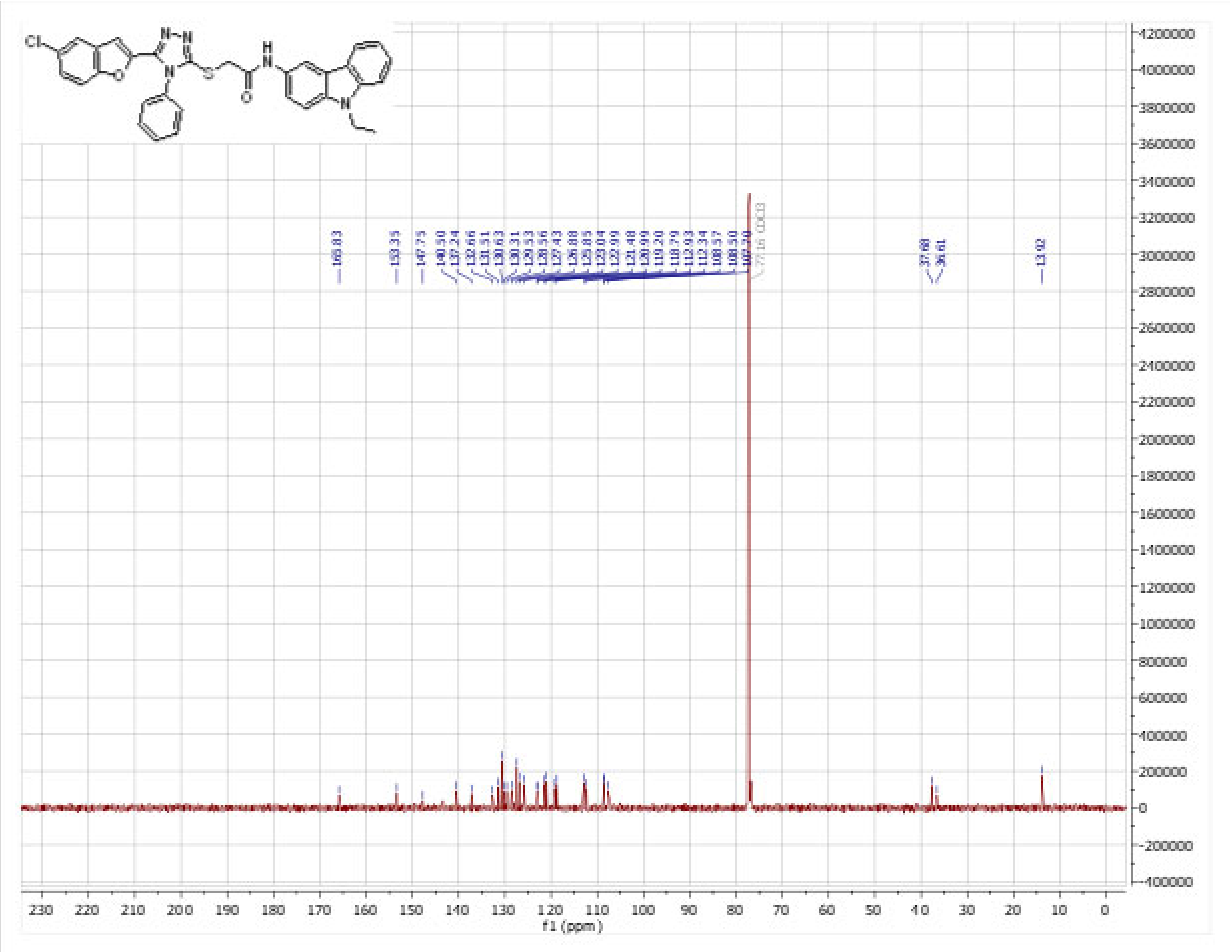


**Figure S10**: $^{13}$C NMR of compound **9e**.

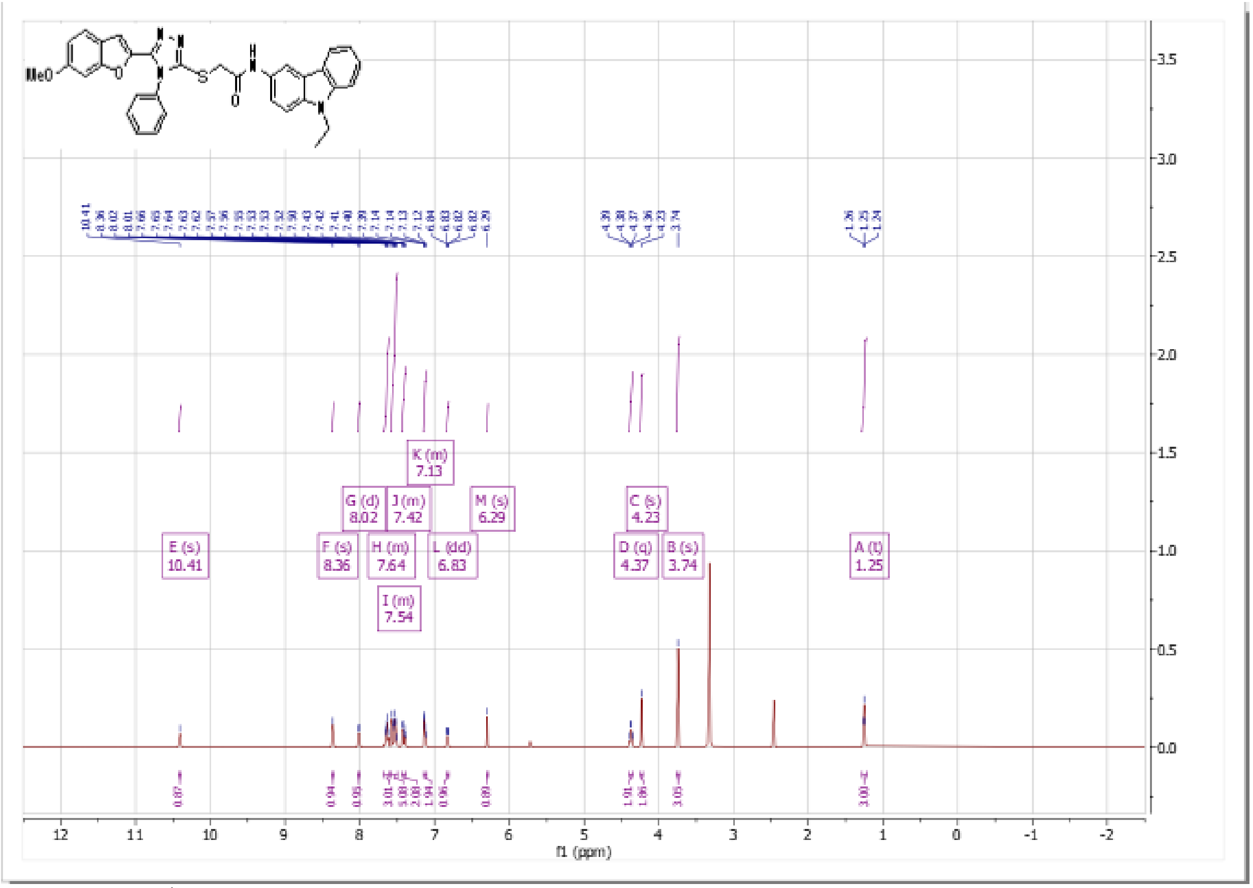


**Figure S11**: $^{1}$H NMR of compound **9f**.

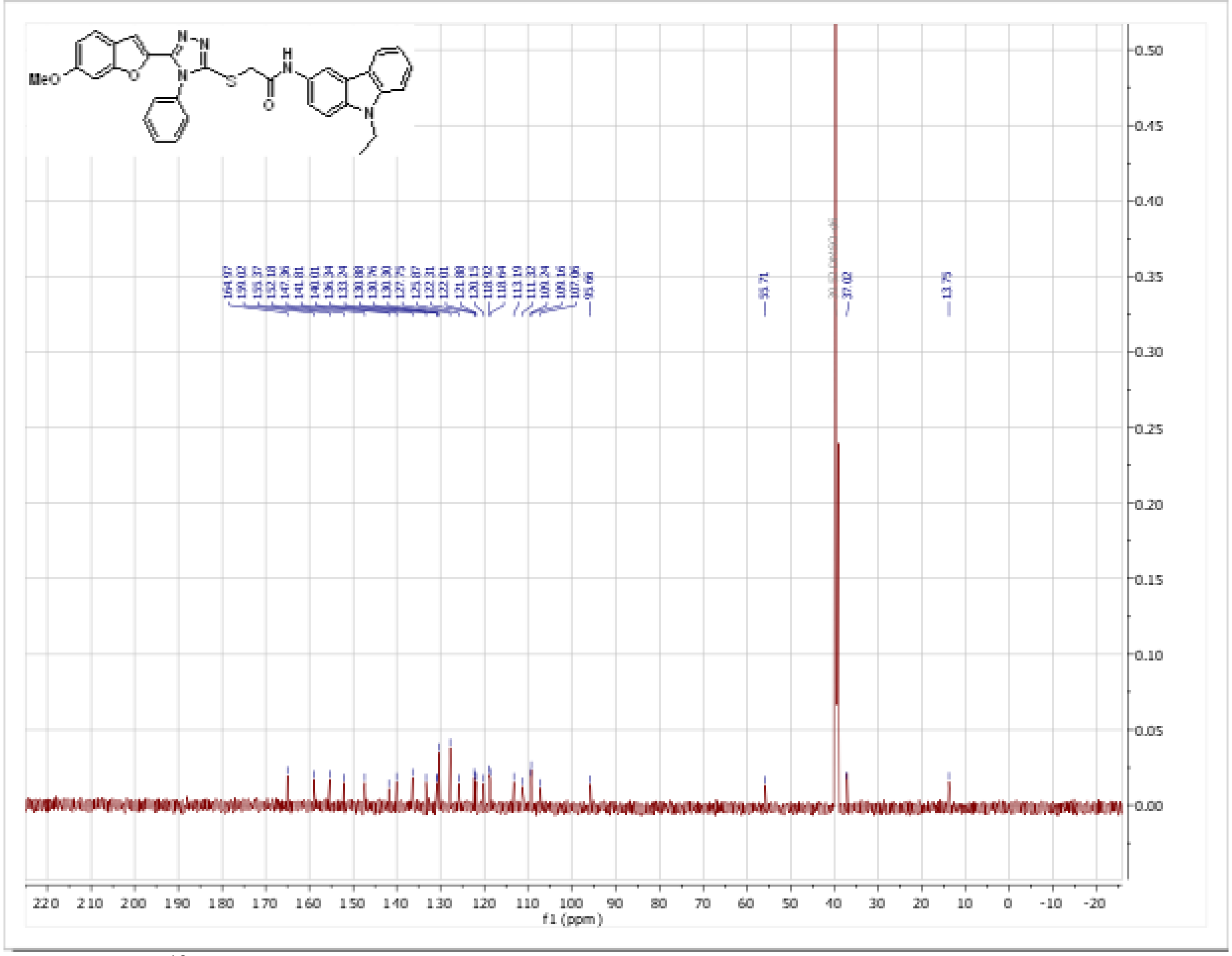

**Figure S12**: $^{13}$C NMR of compound **9f**.

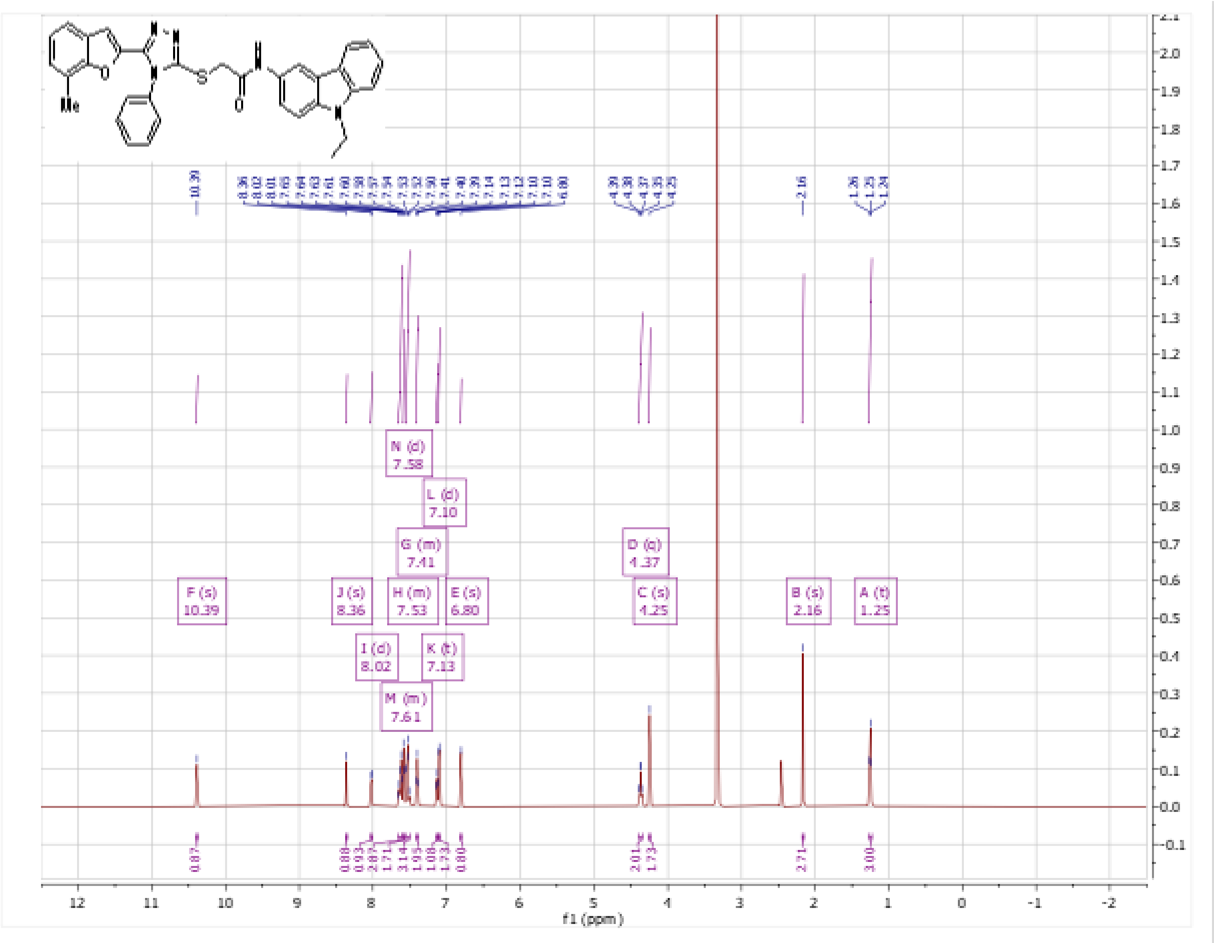


**Figure S13**: $^{1}$H NMR of compound **9g**.

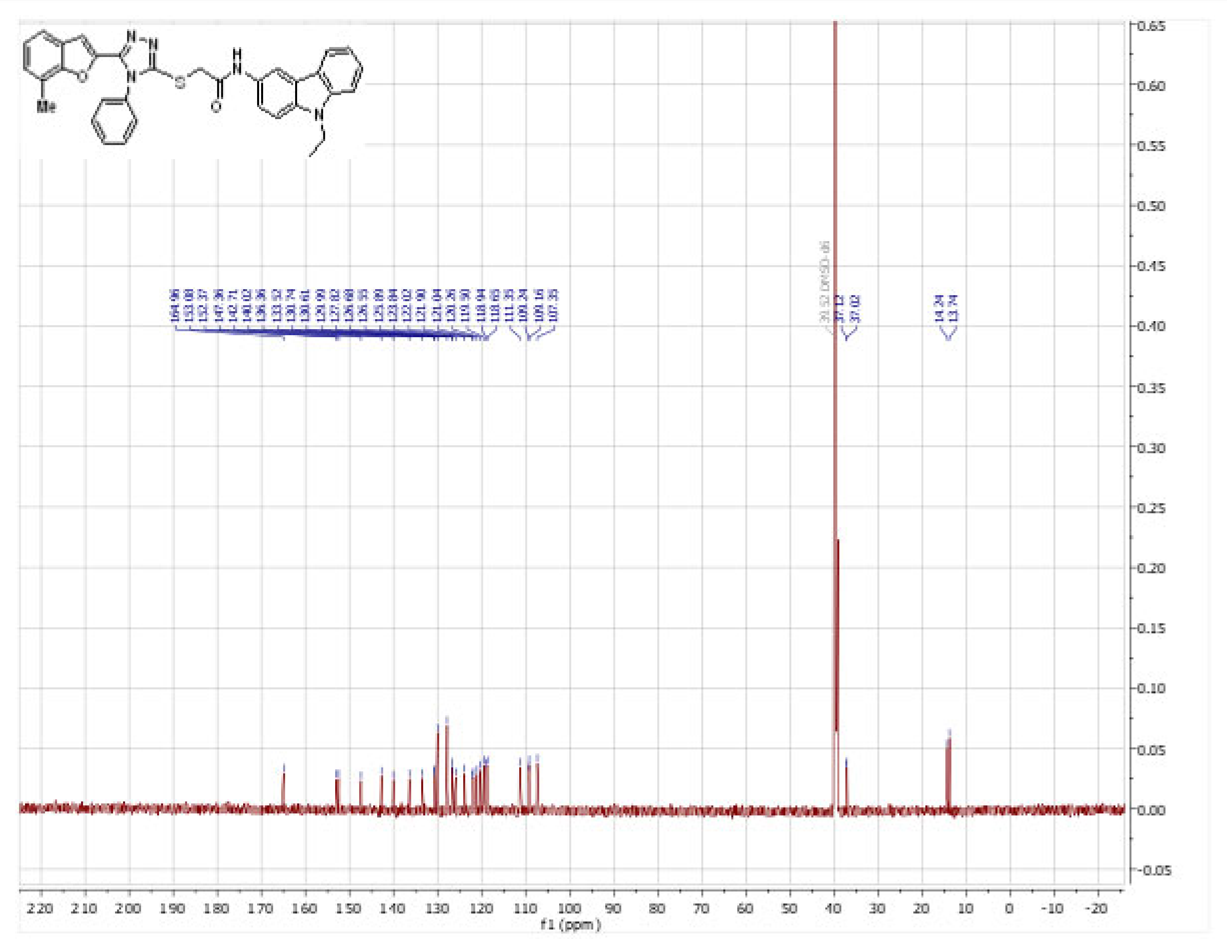

**Figure S14**: $^{13}$C NMR of compound **9g**.

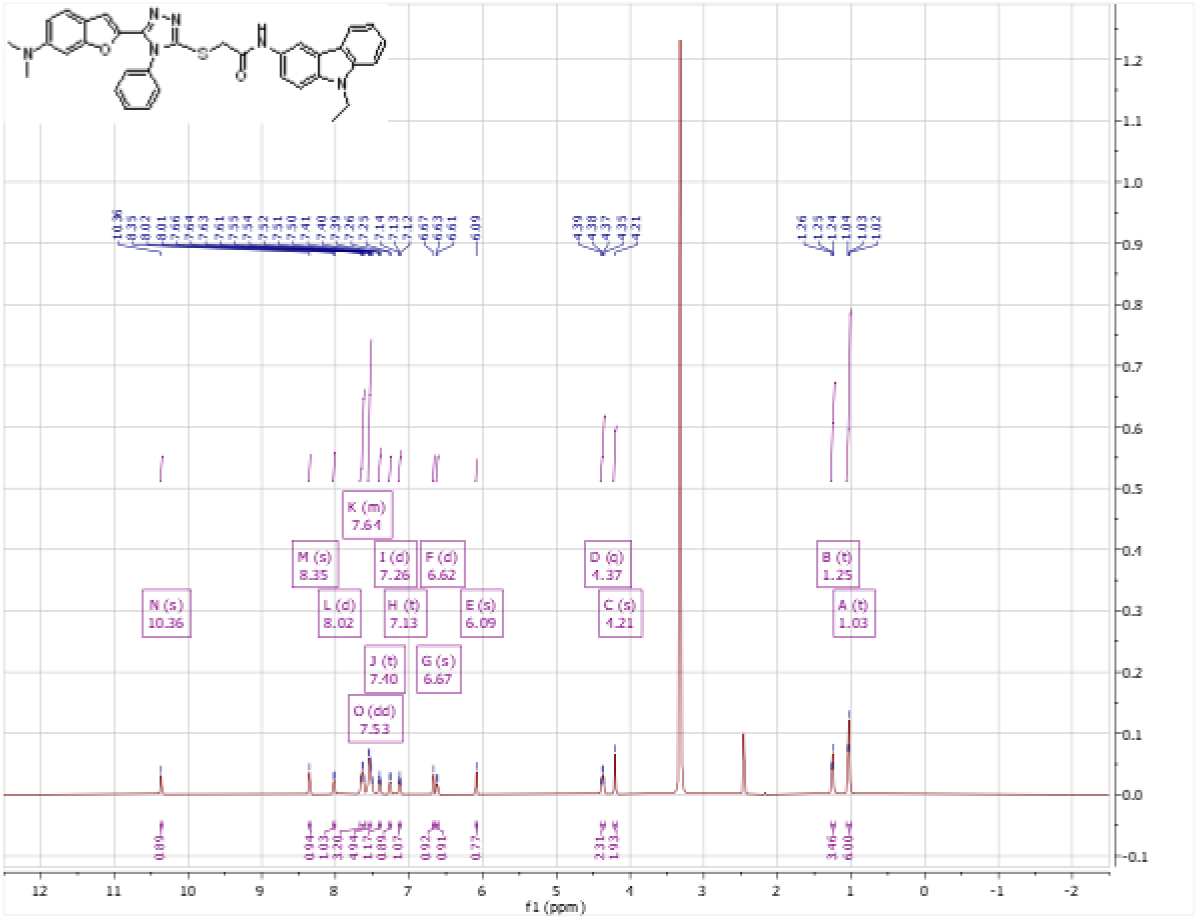


**Figure S15**: $^{1}$H NMR of compound **9h**.

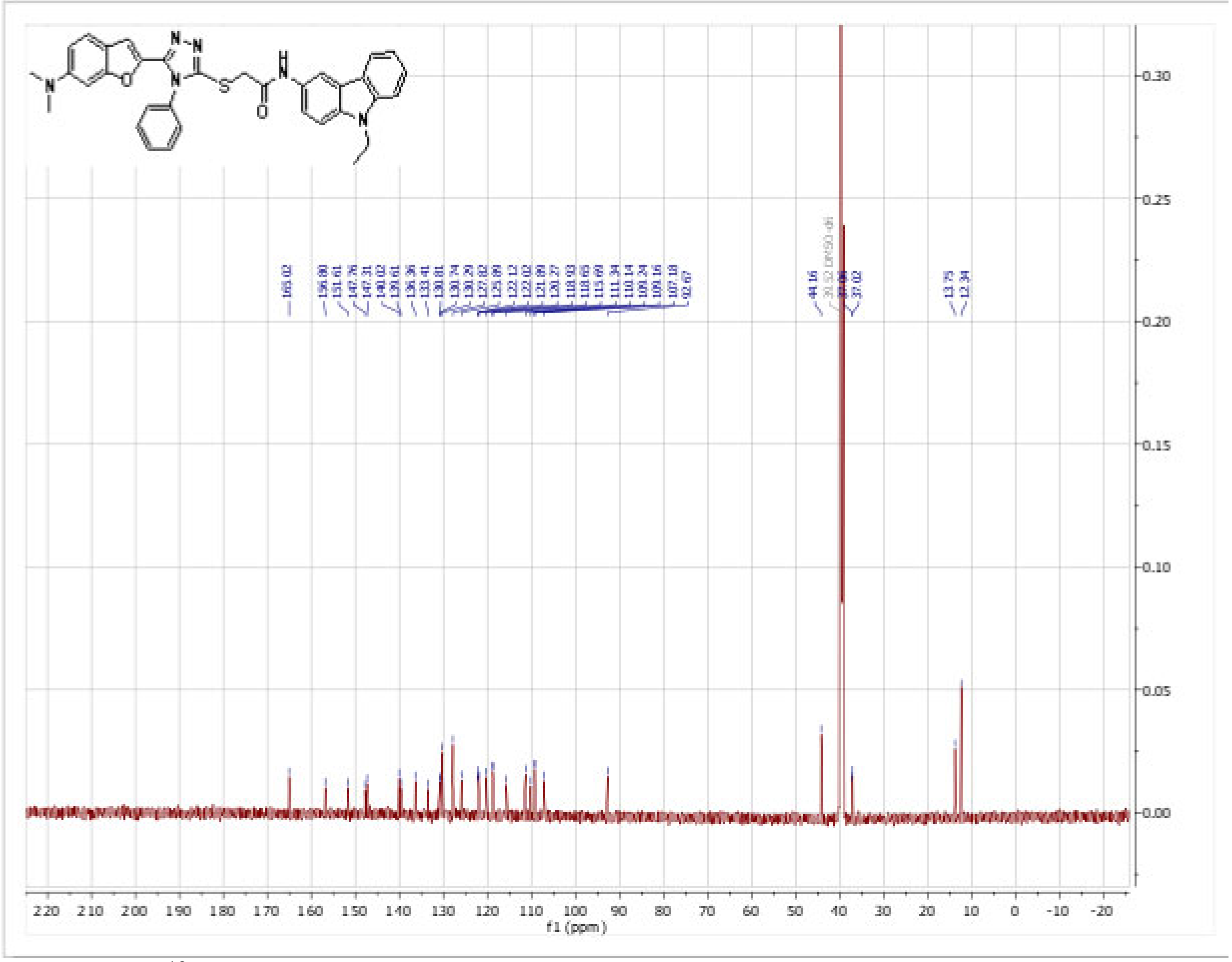


**Figure S16**: $^{13}$C NMR of compound **9h**.

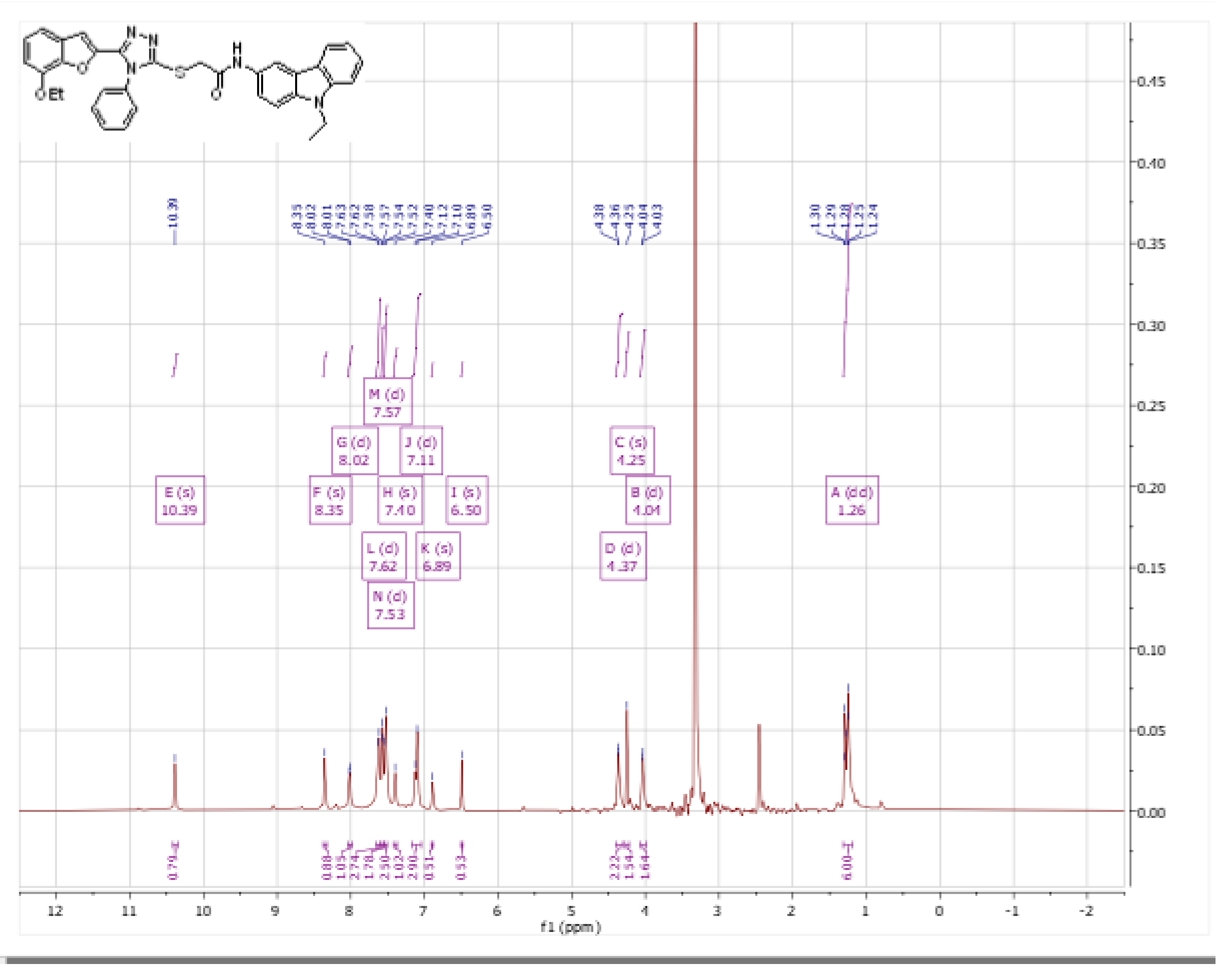


**Figure S17**: $^{1}$H NMR of compound **9i**.

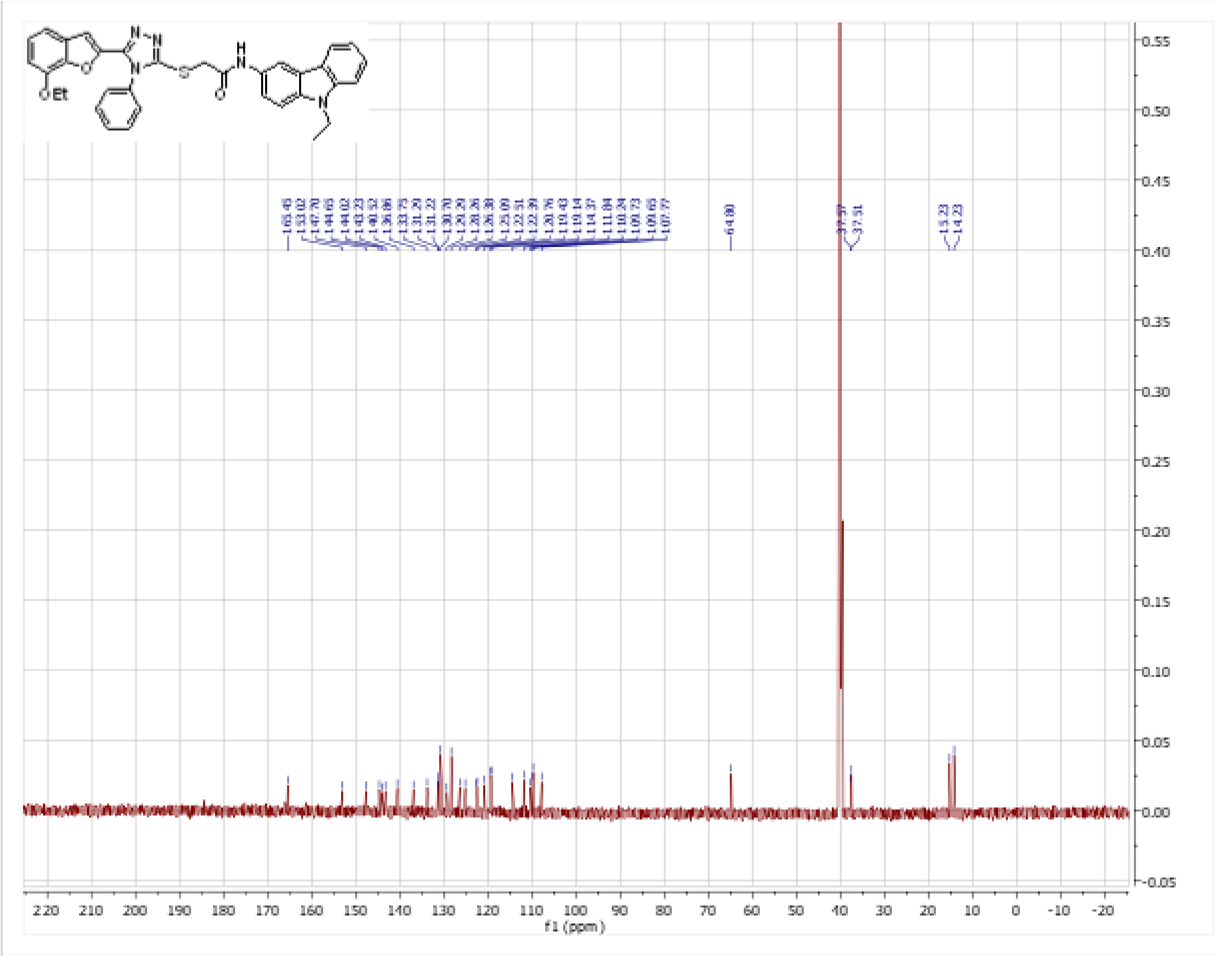


**Figure S18**: $^{13}C$ NMR of compound **9i**.

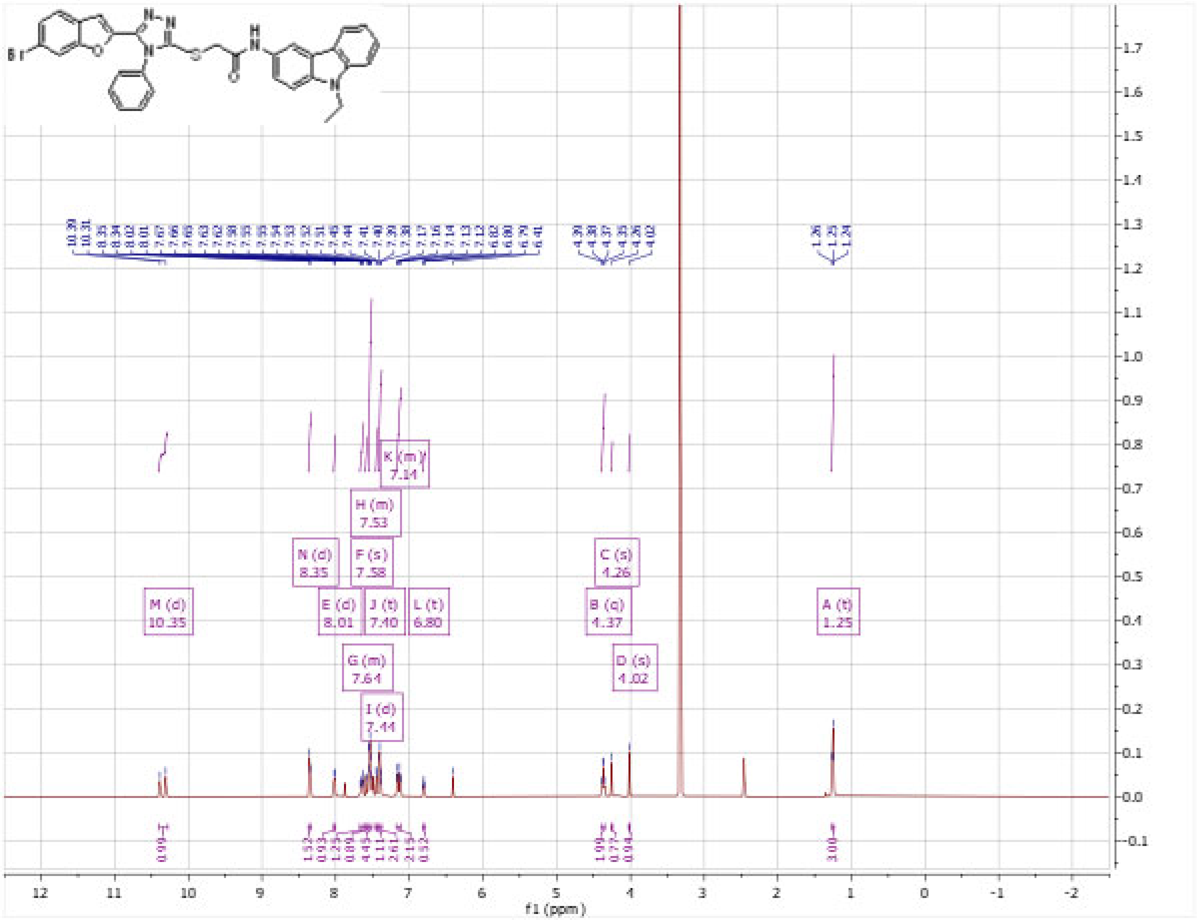

**Figure S19**: $^{1}$H NMR of compound **9j**.

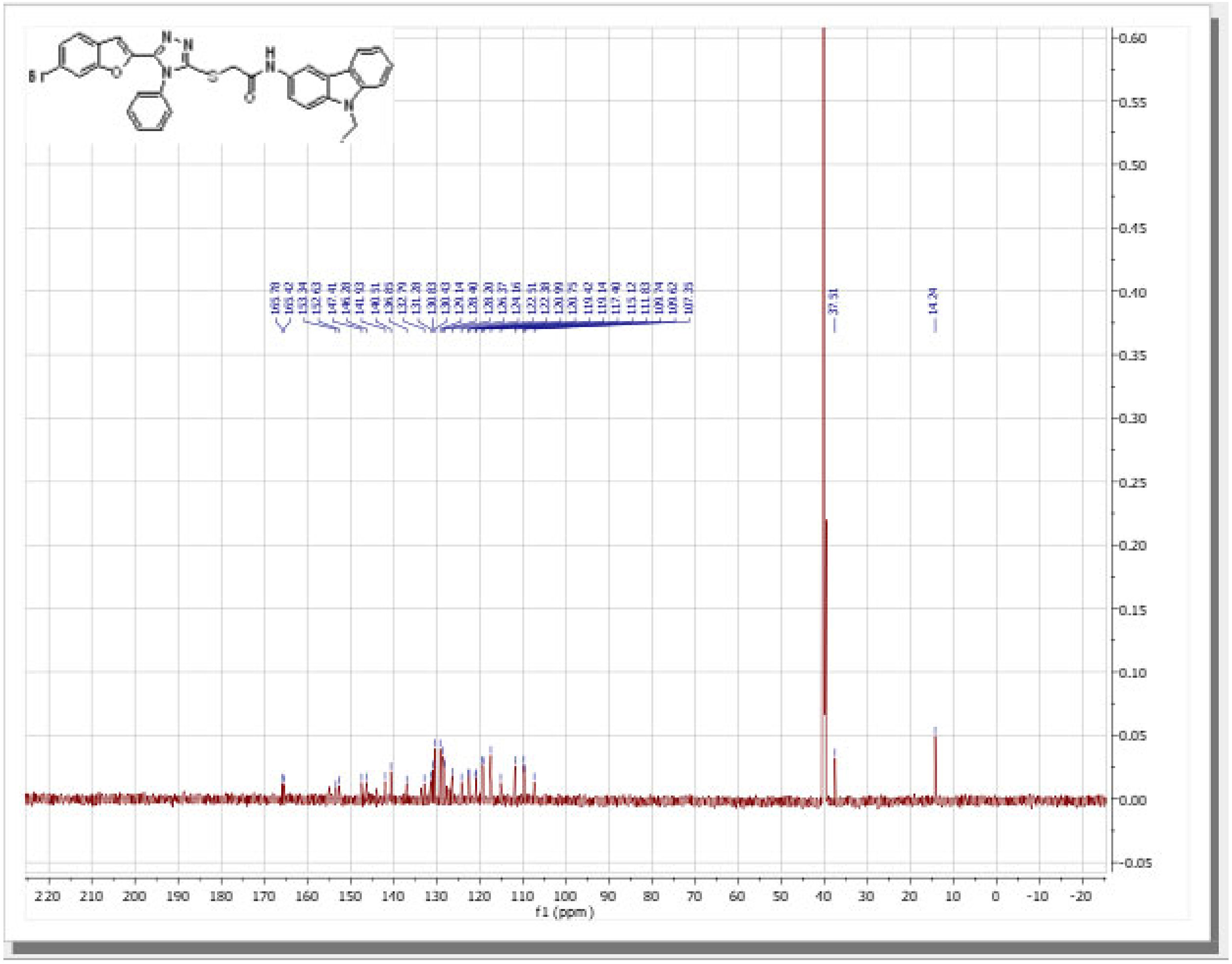


**Figure S20**: $^{13}$C NMR of compound **9j**.

**Table 1:** Calculated binding energies of the synthesized compounds and FDA-approved drugs against selected proteins.

| Sr. No. | Compound Code | Structure | 6lu7 | 6wpt | 6m71 |
|---|---|---|---|---|---|
| 1- | **9a** | 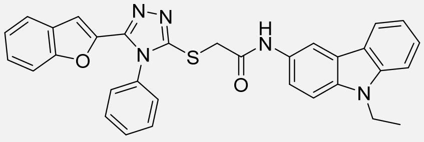 | -8.5249 | -6.146 | -6.5752 |
| 2- | **9b** | 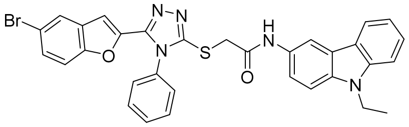 | -8.8316 | -6.4390 | -6.6509 |
| 3- | **9c** | 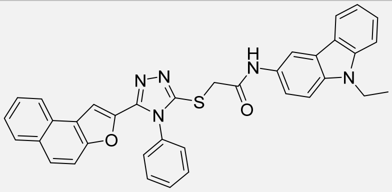 | -8.9203 | -5.9347 | -6.9193 |
| 4- | **9d** | 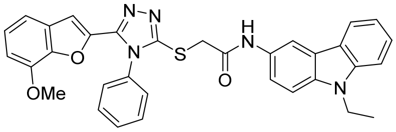 | -8.4681 | -6.3866 | -6.5356 |
| 5- | **9e** | 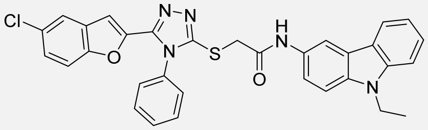 | -8.7770 | -6.6934 | -7.6139 |
| 6- | **9f** | 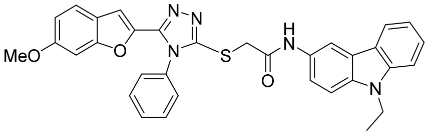 | -8.5097 | -6.4124 | -7.2549 |
| 7- | **9g** | 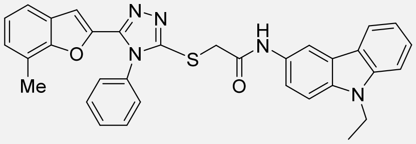 | -8.4871 | -5.6443 | -7.6298 |

| | | | | | |
|---|---|---|---|---|---|
| **8-** | **9h** | 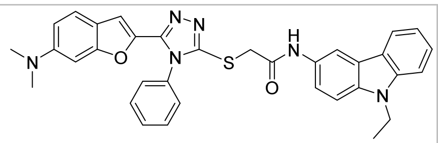 | -8.7630 | -6.5387 | -8.1019 |
| **9-** | **9i** | 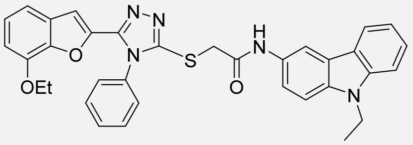 | -8.8703 | -6.4397 | -8.0065 |
| **10-** | **9j** | 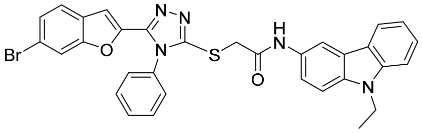 | -8.8492 | -6.5619 | -7.5397 |
| **11-** | **Remdesivir** | 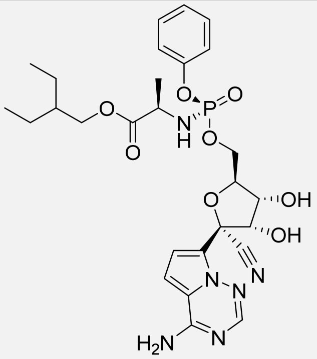 | -8.5523 | -6.2612 | -6.8211 |
| **12-** | **Lopinavir** | 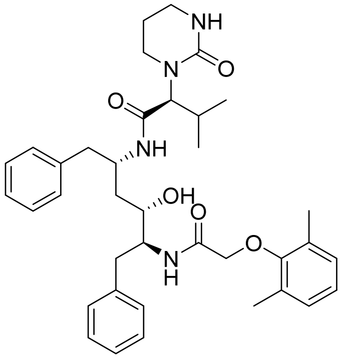 | -8.7372 | -6.099 | -7.4823 |
| **13-** | **Chloroquine** | 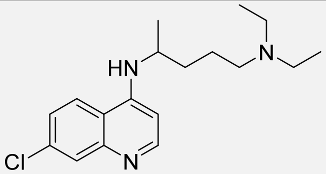 | -6.4026 | -5.1362 | -5.7037 |
| **14-** | **Favipiravir** | 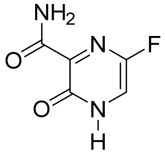 | -4.4833 | -3.9224 | -4.2102 |

| | | | | | |
|---|---|---|---|---|---|
| **15-** | **Nirmatrelvir** | 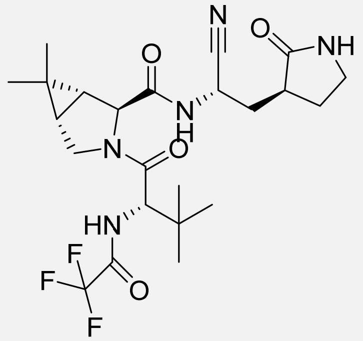 | -8.3860 | -4.8757 | -6.4117 |

**Molecular docking interactions of synthesized compounds with SARS-CoV-2 proteins:**

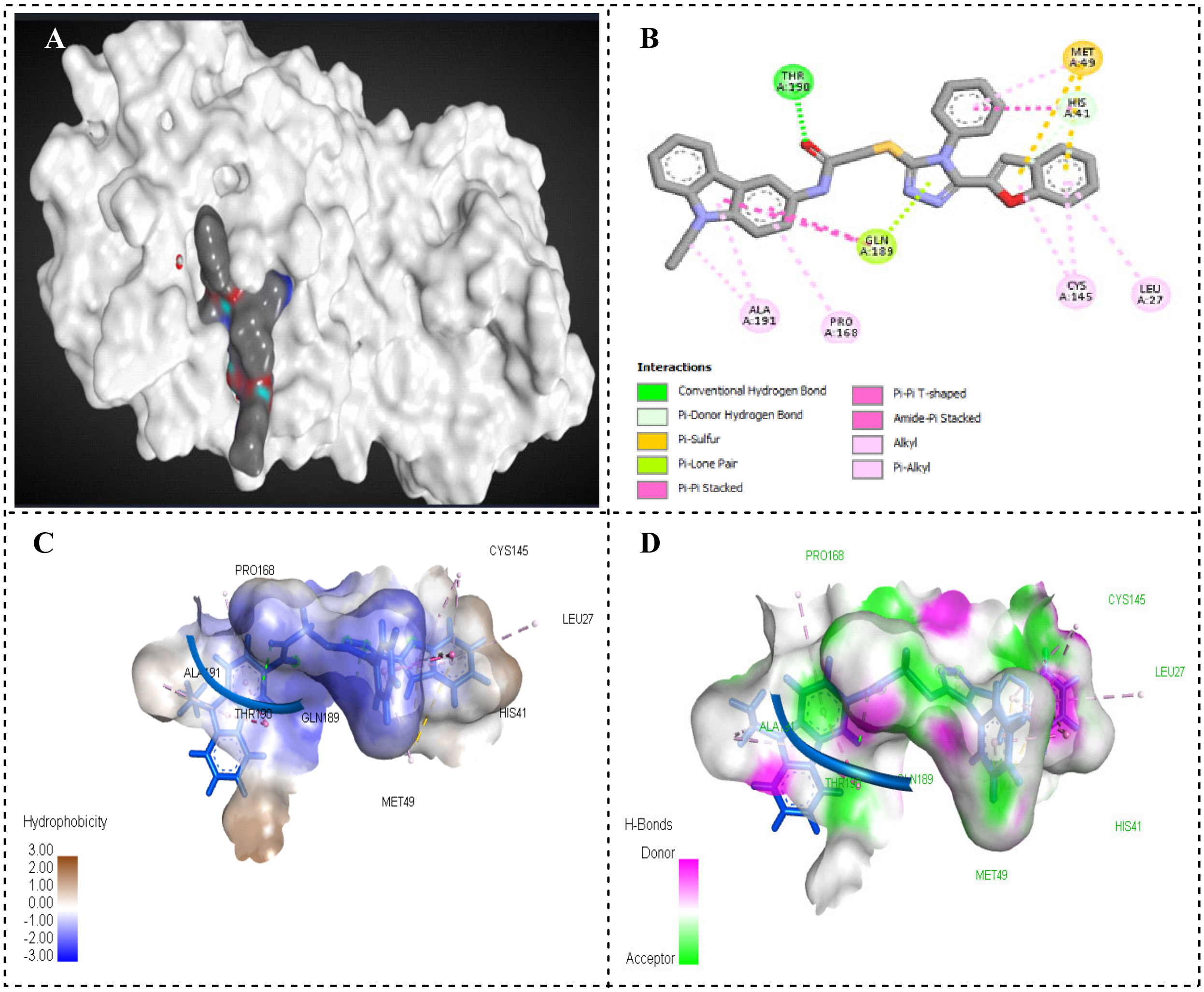


**Figure S21**: Interaction modes of compound **9a**; **A**: Calculated surface of docked **9a** with main protease, **B**: 2D interactions, **C**: Hydrophobic interactions, **D**: Hydrogen bonding interactions.

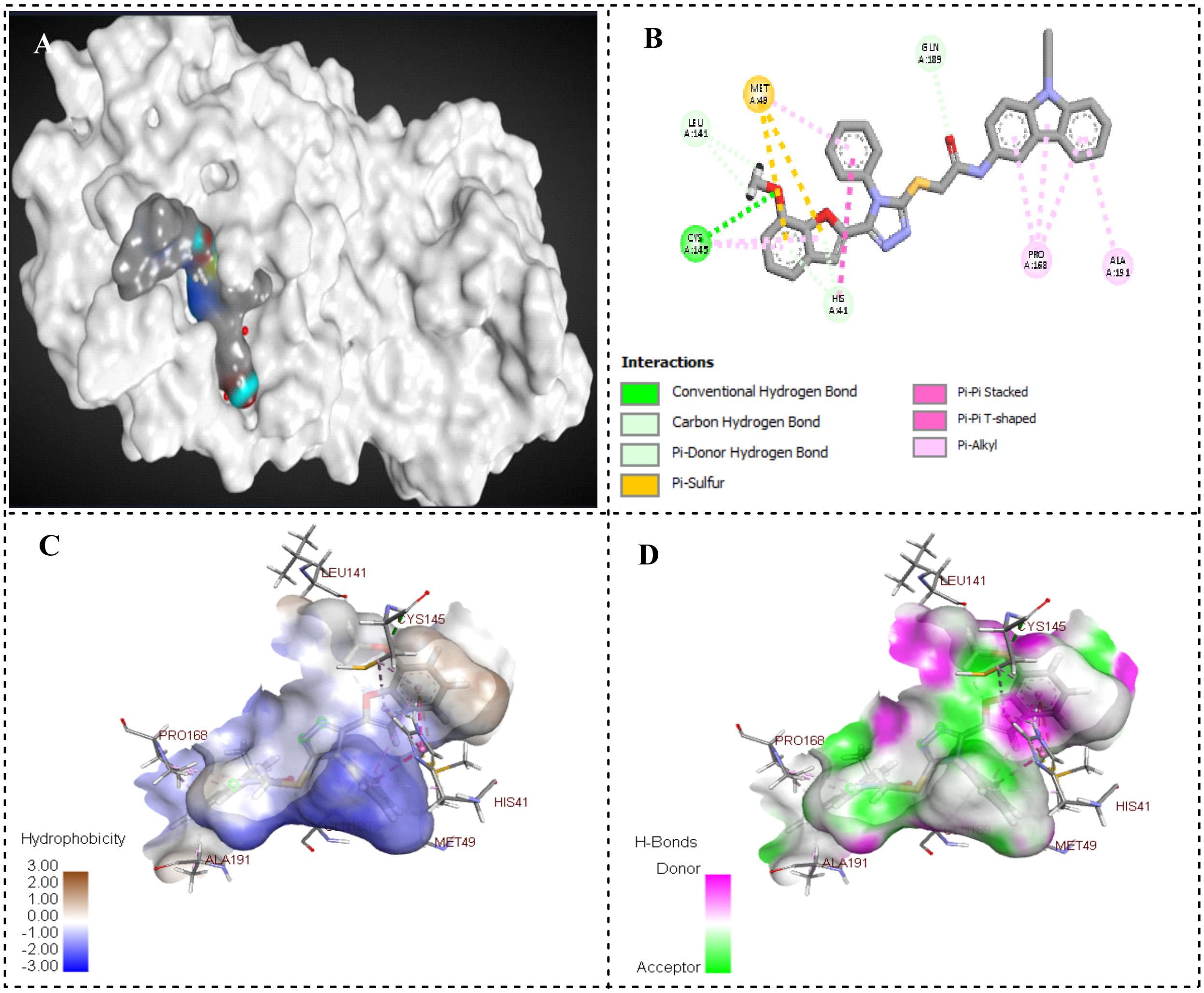


**Figure S22**: Interaction modes of compound **9d**; **A**: Calculated surface of docked **9d** with main protease, **B**: 2D interactions, **C**: Hydrophobic interactions, **D**: Hydrogen bonding interactions.

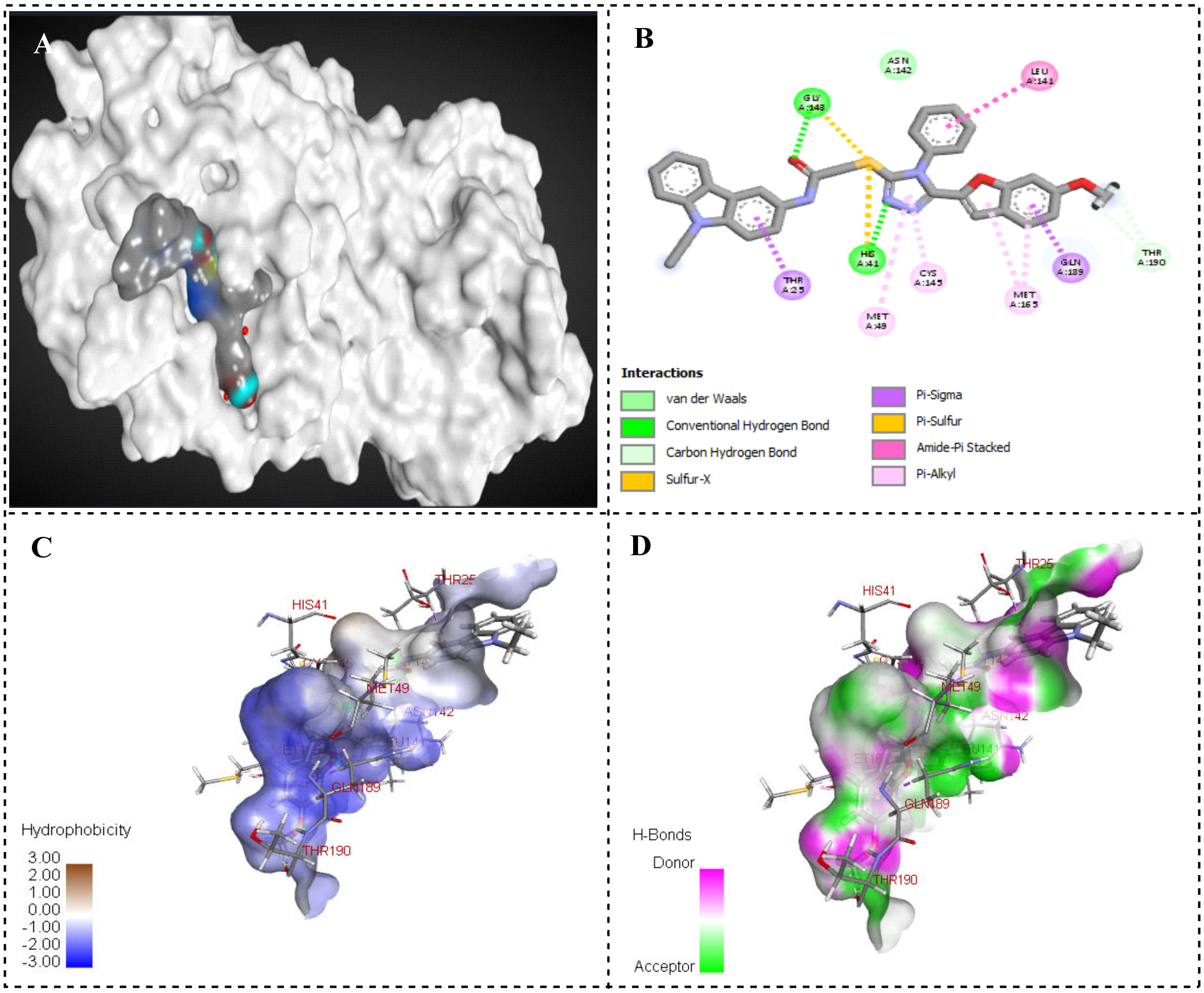


**Figure S23**: Interaction modes of compound **9f**; **A**: Calculated surface of docked **9f** with main protease, **B**: 2D interactions, **C**: Hydrophobic interactions, **D**: Hydrogen bonding interactions.

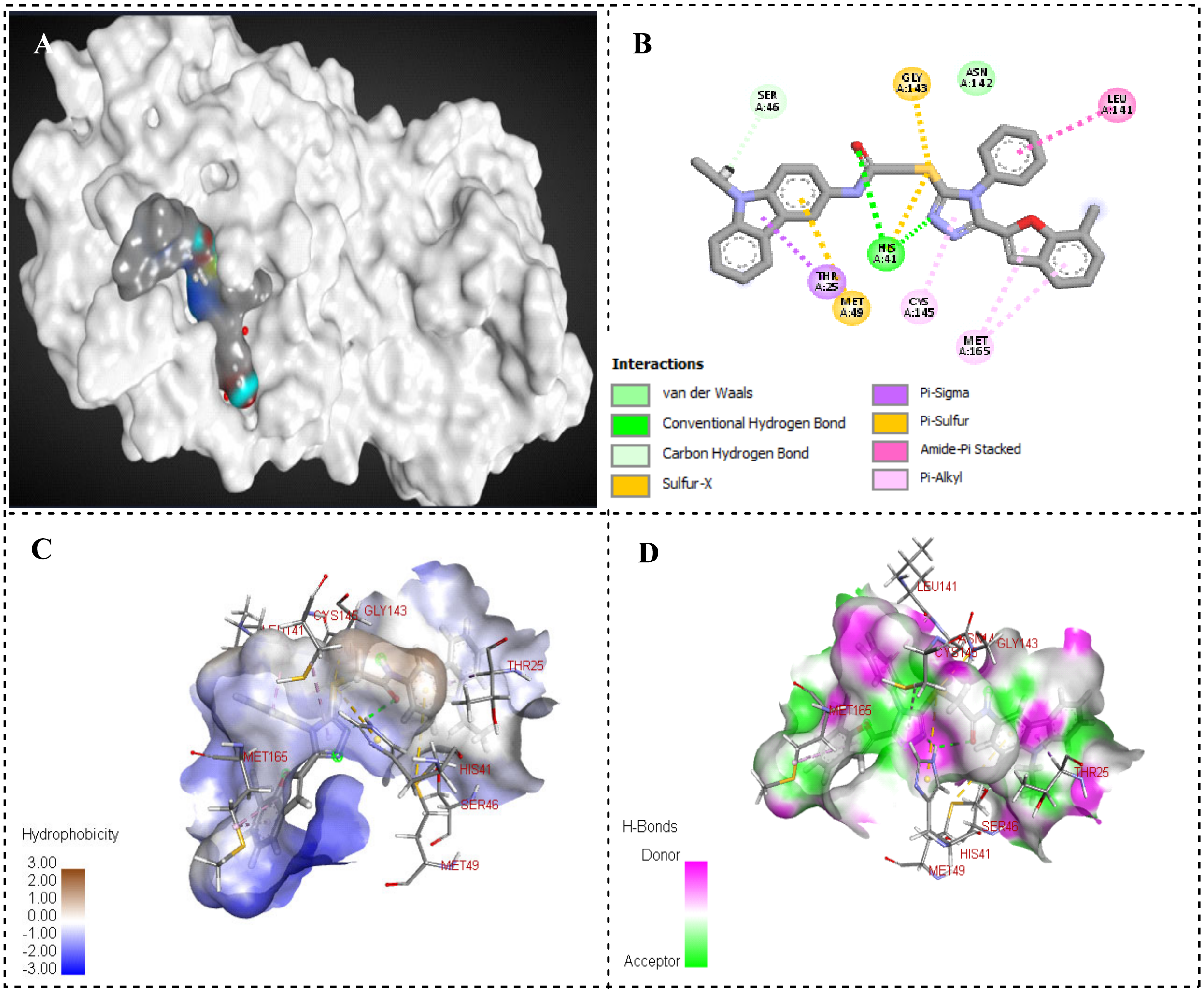


**Figure S24**: Interaction modes of compound **9g**; **A**: Calculated surface of docked **9g** with main protease, **B**: 2D interactions, **C**: Hydrophobic interactions, **D**: Hydrogen bonding interactions.

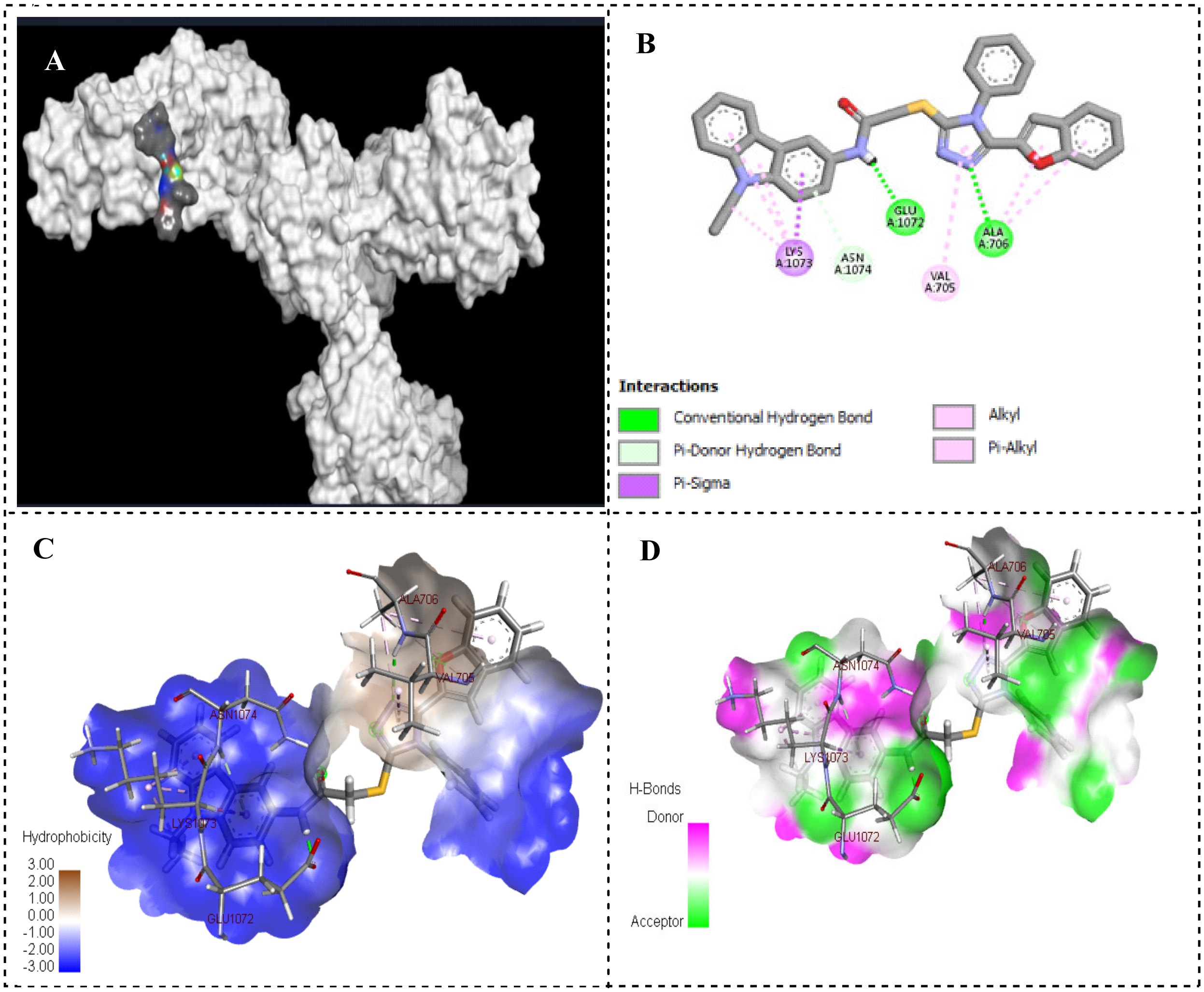


**Figure S25**: Interaction modes of compound **9a**; **A**: Calculated surface of docked **9a** with spike glycoprotein, **B**: 2D interactions, **C**: Hydrophobic interactions, **D**: Hydrogen bonding interactions.

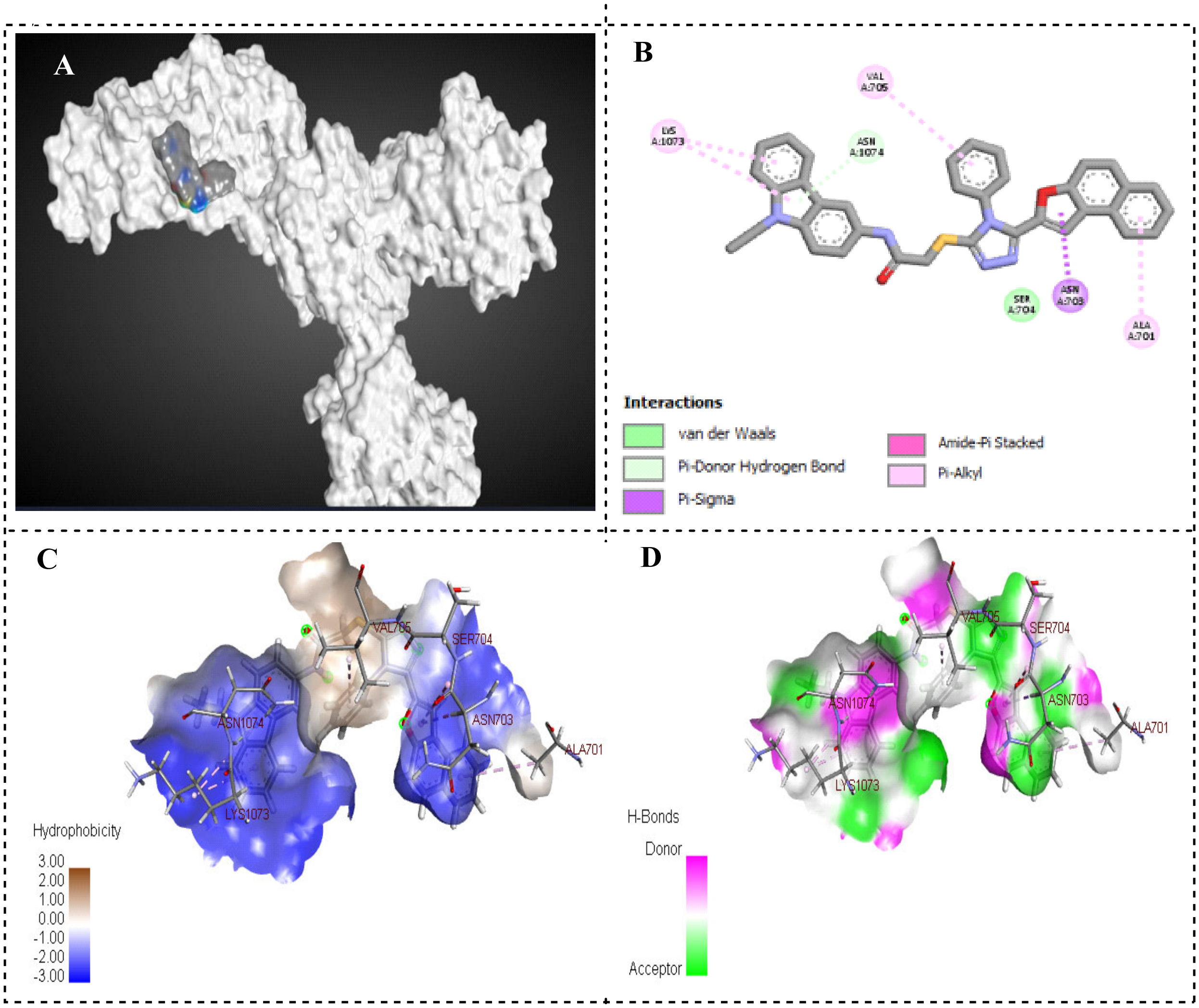


**Figure S26**: Interaction modes of compound **9c**; **A**: Calculated surface of docked **9c** with spike glycoprotein, **B**: 2D interactions, **C**: Hydrophobic interactions, **D**: Hydrogen bonding interactions.

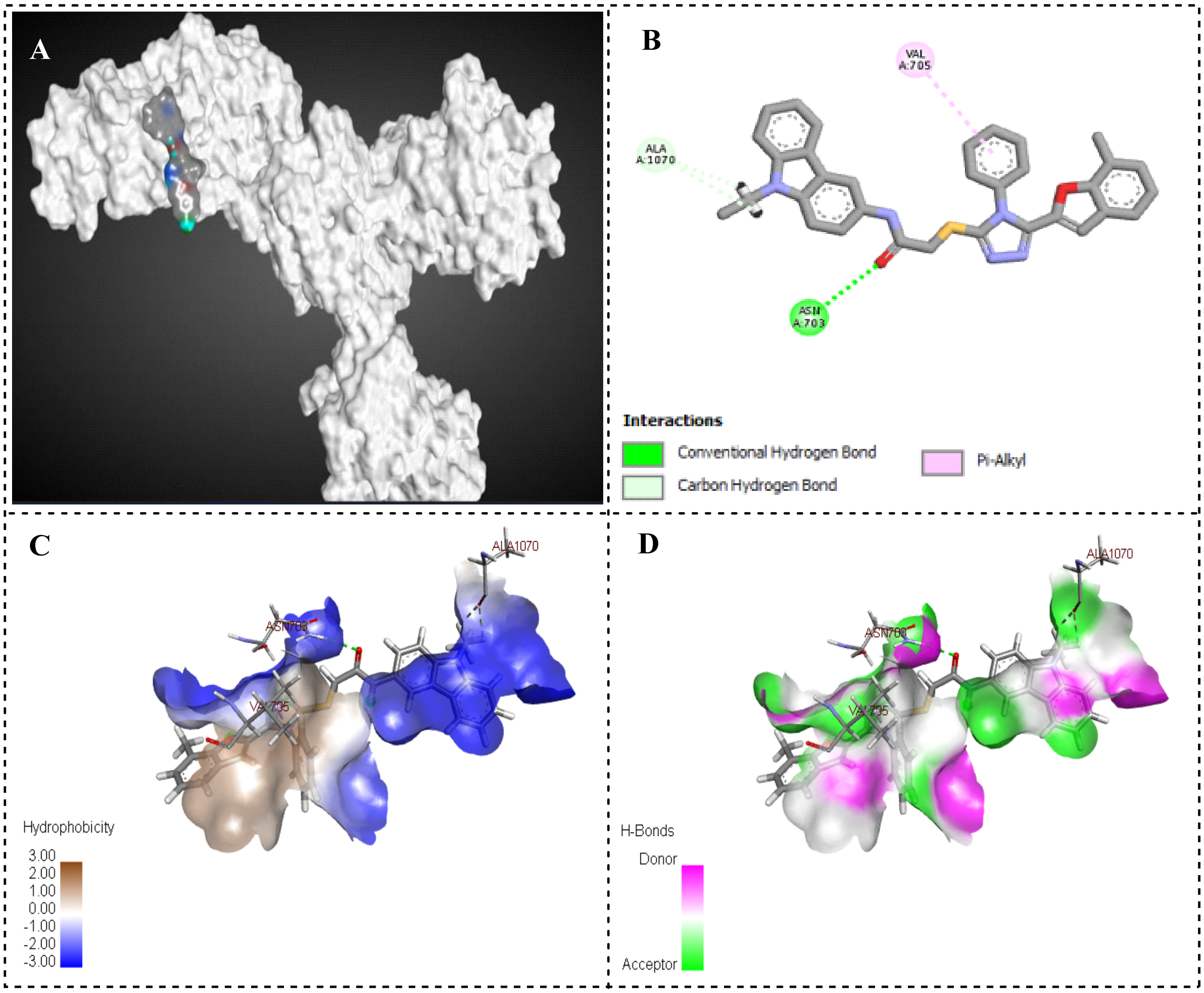


**Figure S27**: Interaction modes of compound **9g**; **A**: Calculated surface of docked **9g** with spike glycoprotein, **B**: 2D interactions, **C**: Hydrophobic interactions, **D**: Hydrogen bonding interactions.

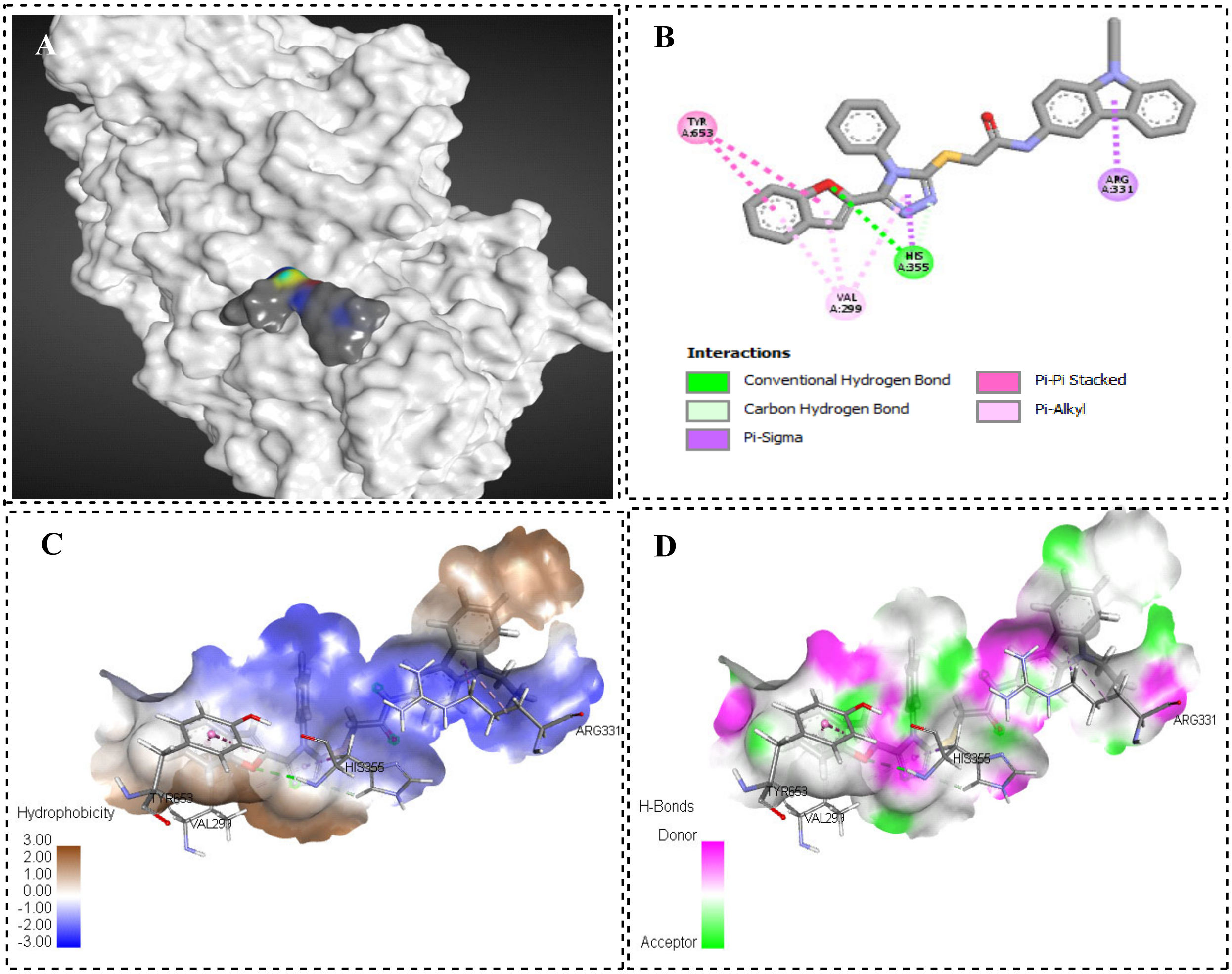


**Figure S28**: Interaction modes of compound **9a**; **A**: Calculated surface of docked **9a** with RdRp, **B**: 2D interactions, **C**: Hydrophobic interactions, **D**: Hydrogen bonding interactions.

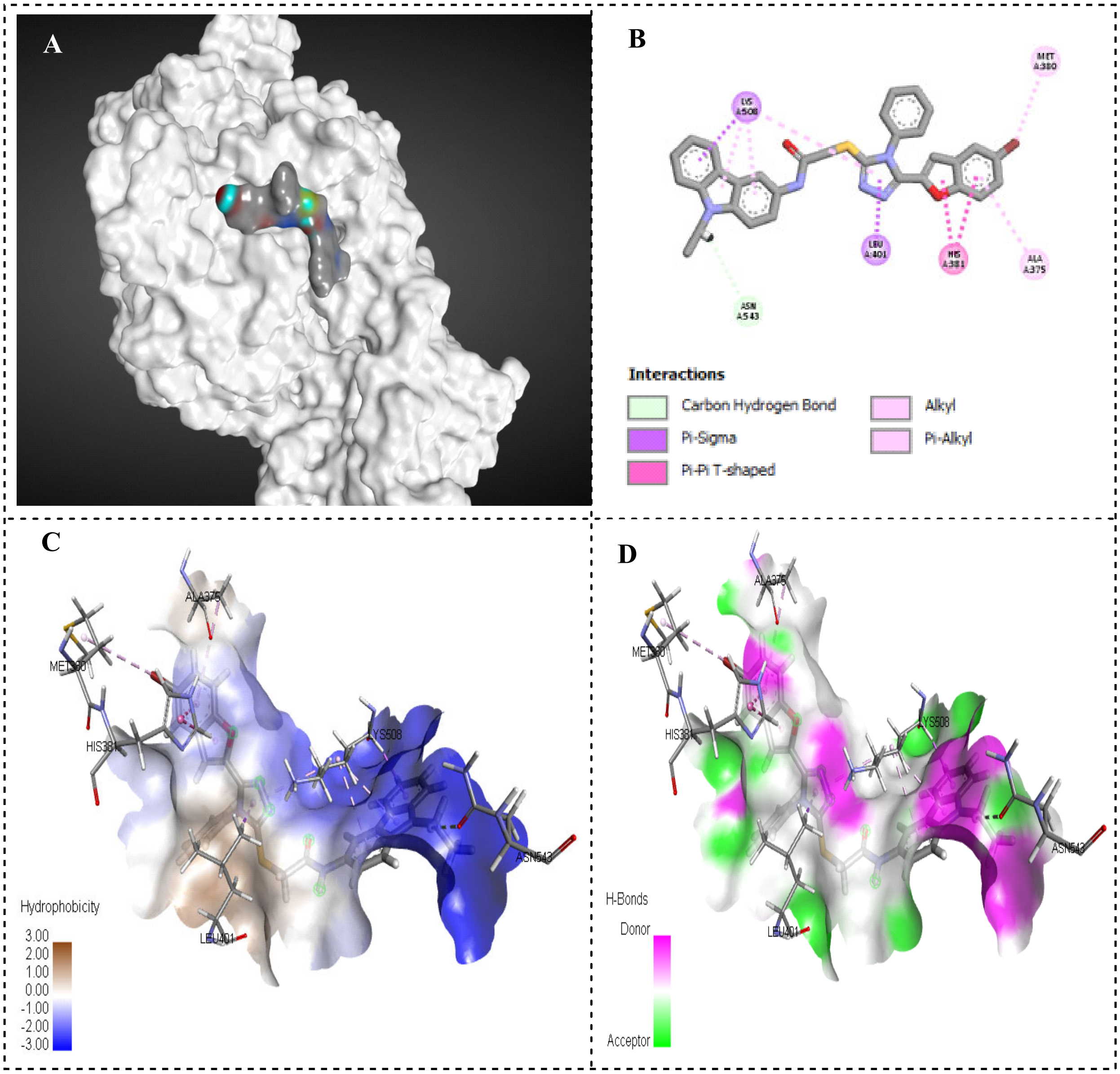


**Figure S29**: Interaction modes of compound **9b**; **A**: Calculated surface of docked **9b** with RdRp, **B**: 2D interactions, **C**: Hydrophobic interactions, **D**: Hydrogen bonding interactions.

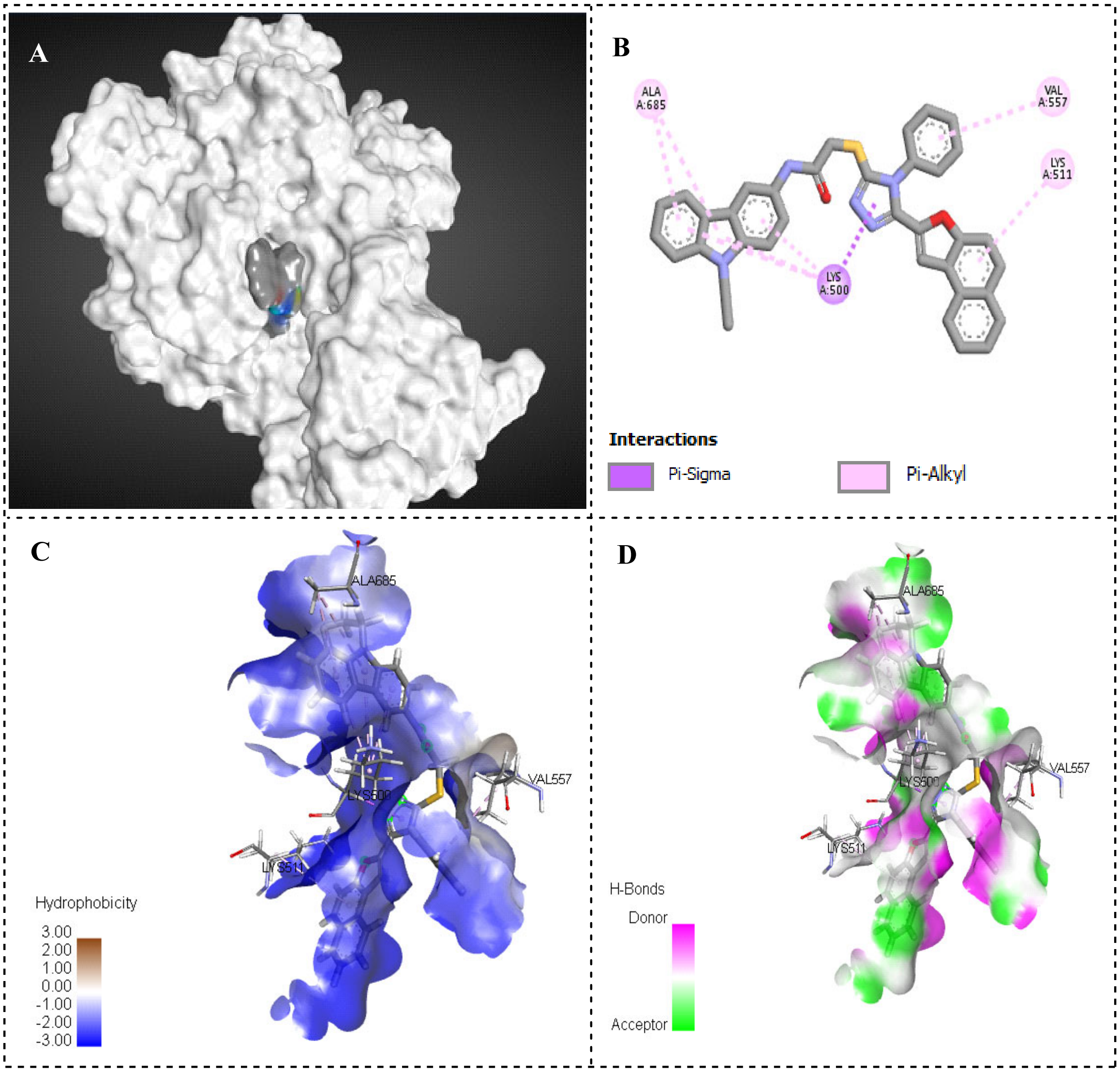


**Figure S30**: Interaction modes of compound **9c**; **A**: Calculated surface of docked **9c** with RdRp, **B**: 2D interactions, **C**: Hydrophobic interactions, **D**: Hydrogen bonding interactions.

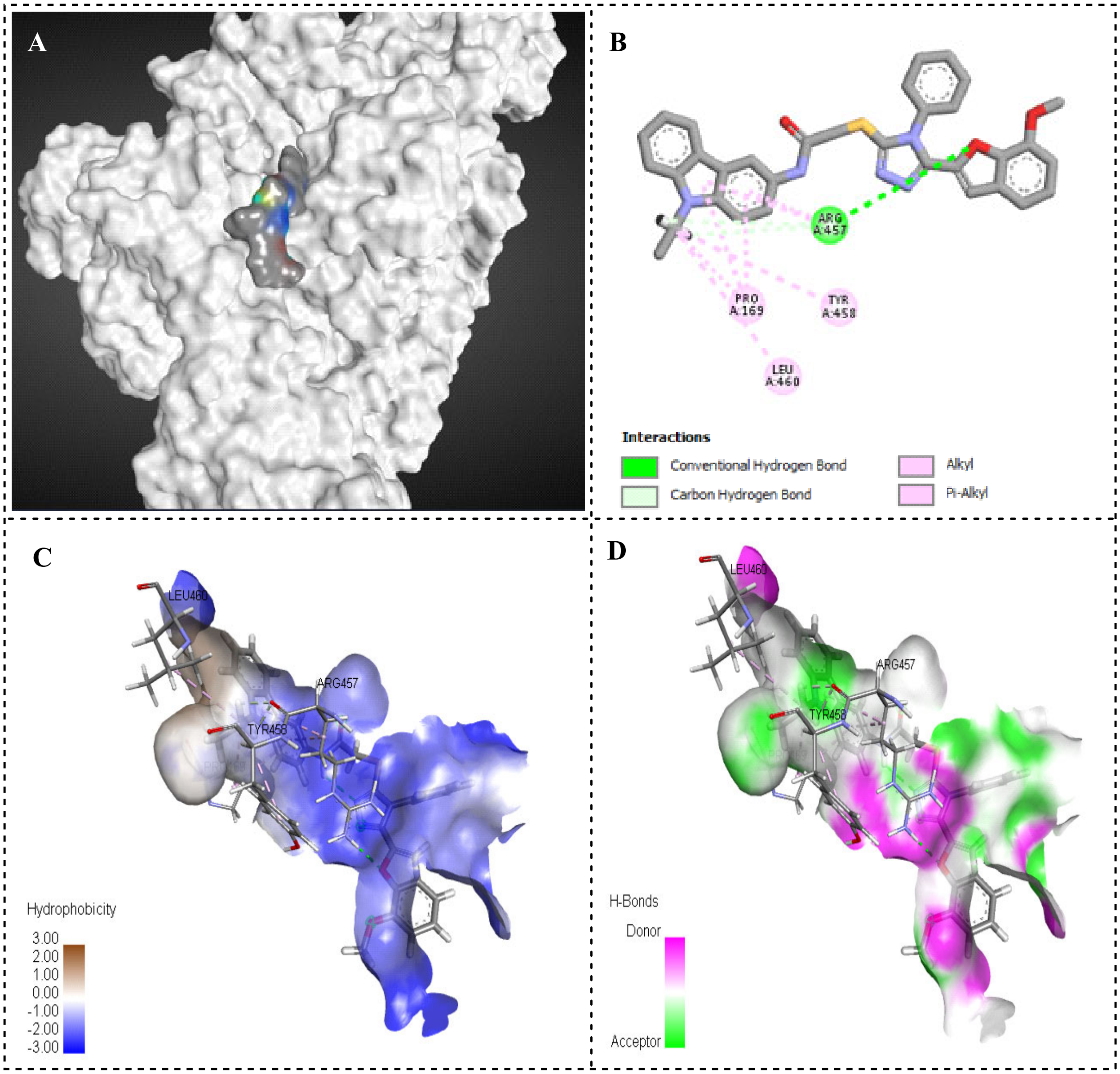


**Figure S31**: Interaction modes of compound **9d**; **A**: The calculated surface of docked **9d** with RdRp, **B**: 2D interactions, **C**: Hydrophobic interactions, **D**: Hydrogen bonding interactions.

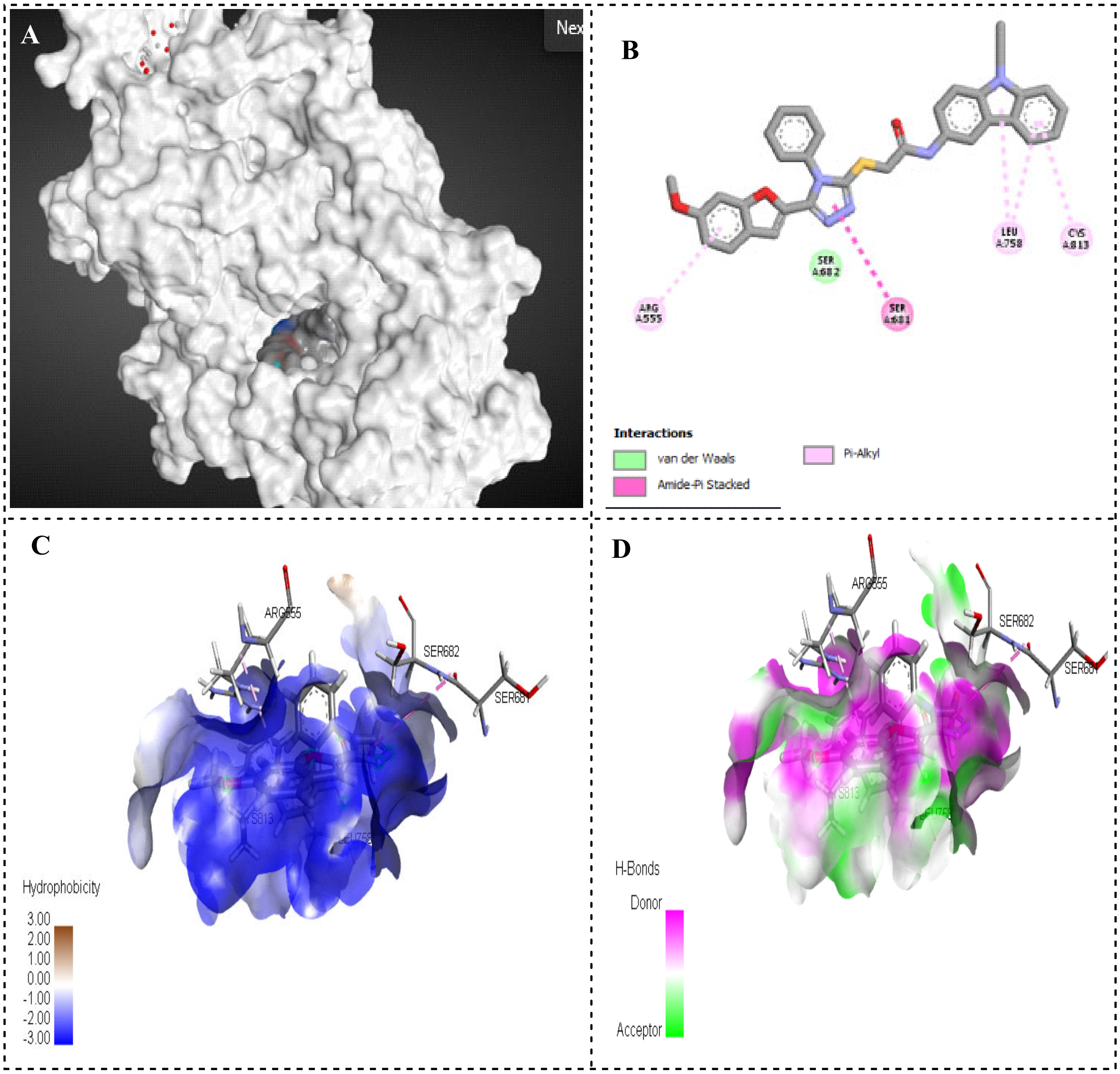


**Figure S32**: Interaction modes of compound **9f**; **A**: The calculated surface of docked **9f** with RdRp, **B**: 2D interactions, **C**: Hydrophobic interactions, **D**: Hydrogen bonding interactions.

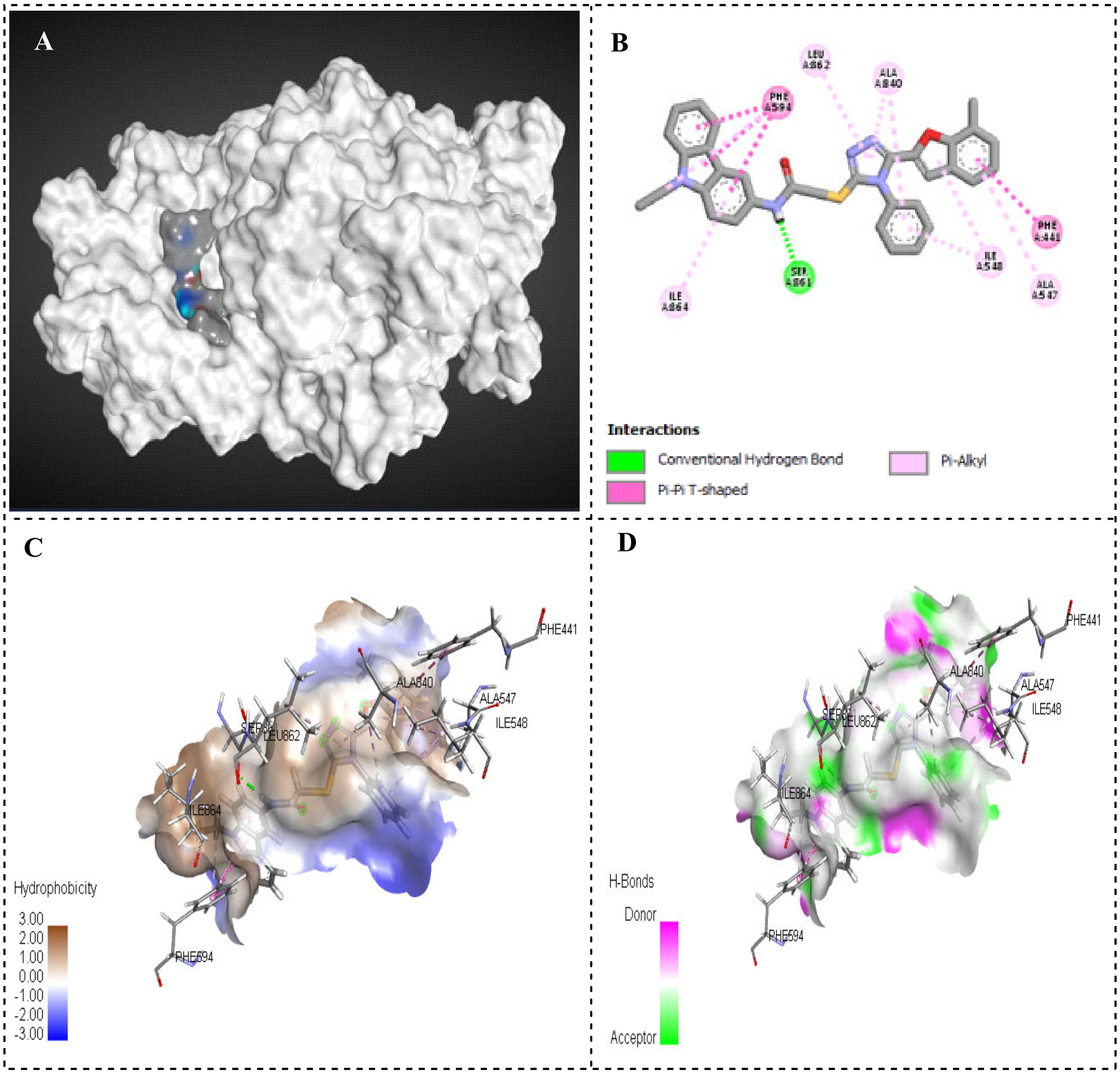


**Figure S33**: Interaction modes of compound **9g**; **A**: Calculated surface of docked **9g** with RdRp, **B**: 2D interactions, **C**: Hydrophobic interactions, **D**: Hydrogen bonding interactions.

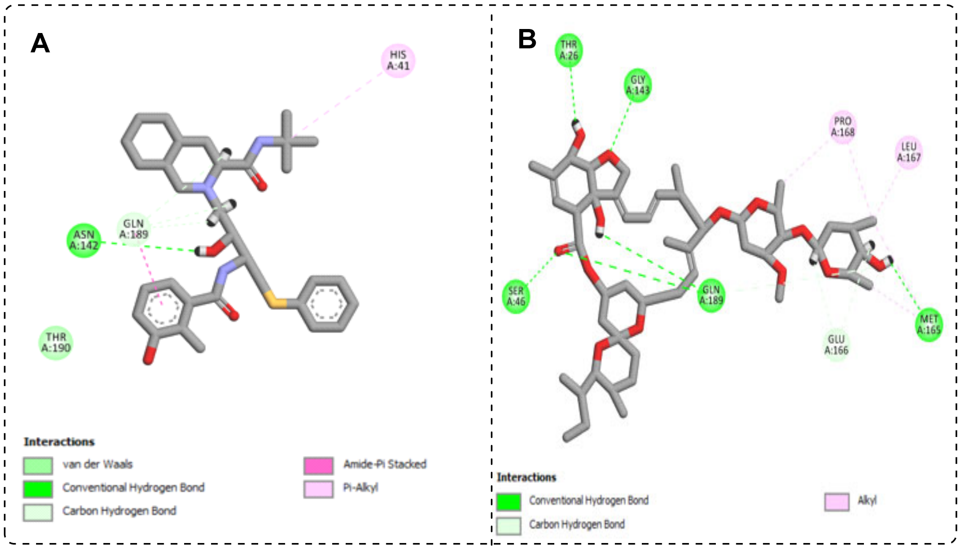


**Figure S34.** Binding interaction of **A**:Nelfinavir and **B**: Ivermectin with Mpro**.**

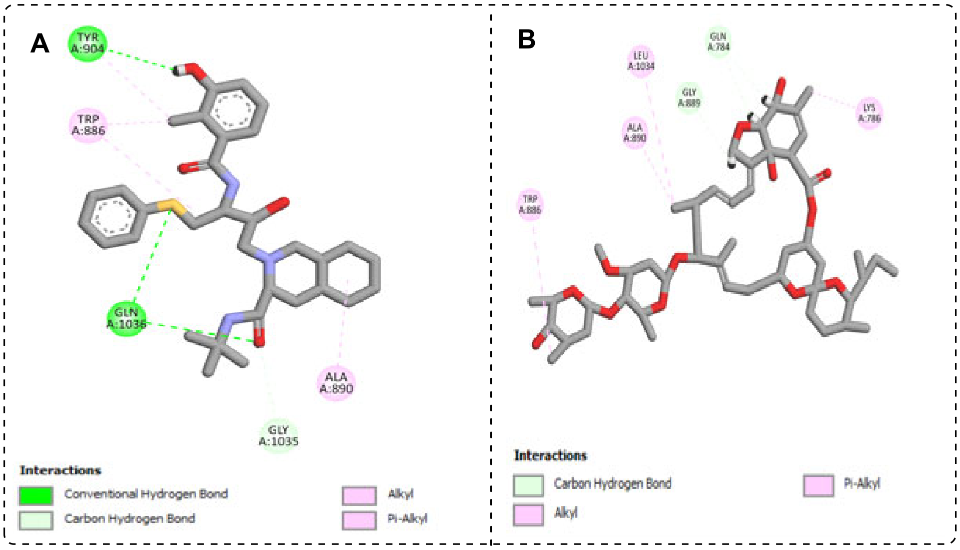


**Figure S35.** Binding interaction of **A**:Nelfinavir and **B**: Ivermectin with spike glycoprotein**.**

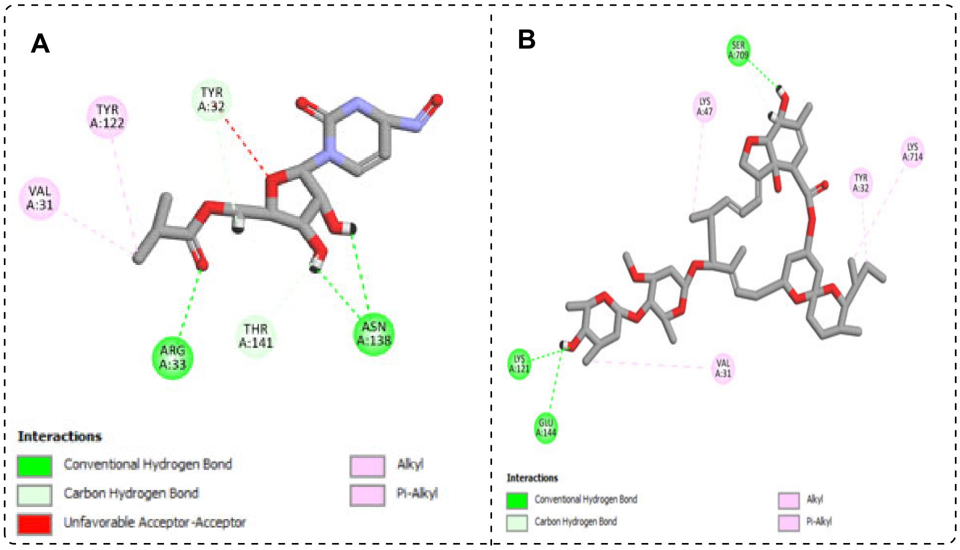


**Figure S36.** Binding interaction of **A**: Molnupiravir and **B**: Ivermectin with RdRp**.**
